# Homeostasis-Inspired, Quality-Adaptive Preprocessing for Multi-Label ECG Diagnosis: A Pre-Registered Multi-Objective Evolutionary Study with Negative Findings and Falsifiable Design Principles

**DOI:** 10.3390/biomimetics11100744

**Published:** 2026-10-15

**Authors:** Hüseyin Yanık

**Affiliations:** Department of Information Systems and Technologies, Mersin University, 33343 Mersin, Türkiye; huseyinyanik@mersin.edu.tr

**Keywords:** electrocardiography, PTB-XL, homeostasis, bio-inspired control, signal quality index, multi-objective optimization, NSGA-II, calibration, robustness, multi-label classification

## Abstract

Deep-learning electrocardiogram (ECG) classifiers usually apply one fixed preprocessing filter to every recording, although signal quality varies. Inspired by physiological homeostasis, ECG preprocessing was formulated as a sensor–controller–effector loop: a weakly supervised quality sensor assigns each 12-lead record to a quality tier, and a tier-gated policy, shaped by Non-dominated Sorting Genetic Algorithm II (NSGA-II) on the validation fold, selects a filtering operator and a calibration temperature for a frozen compact Convolutional Neural Networks (CNN). In a pre-registered evaluation on PTB-XL v1.0.3 (21,388 records), the adaptive policy did not outperform the best fixed band-pass filter in discrimination (test macro area under the receiver operating characteristic curve (AUROC 0.9225 vs. 0.9231), worst-tier performance, or calibration, on native data or on any of 15 synthetic and real (MIT-BIH Noise Stress Test) noise benches. Like the fixed filter, it clearly outperformed unfiltered processing under baseline wander (ΔAUROC +0.0047, Holm-adjusted *p* < 0.001). Post hoc analyses explain these negative results: the operator bank offered no tier-dependent trade-off, the worst-tier objective was rarely binding, the sensor saturated because its tier boundaries were fitted on clean data, the sensor cost exceeded the filtering it saved, and the selected operators were unstable across resamples, while the qualitative rule “filter only the disturbed tier” was stable. Of four predictions derived from homeostasis, one was supported, two were supported only partially, and one was refuted. This study distills six falsifiable design principles for bio-inspired adaptive signal processing.

## 1. Introduction

Automated interpretation of the 12-lead electrocardiogram (ECG) has become a benchmark problem for biomedical deep learning. Public, cardiologist-annotated datasets such as PTB-XL [1,2] and standardized evaluation protocols [3] allow convolutional and recurrent architectures to be compared on a common footing, and macro-averaged areas under the receiver operating characteristic curve (AUROC) above 0.92 are routinely reported for the five diagnostic super-classes [3]. Much less attention has been paid to what happens before the network: almost all pipelines apply one fixed preprocessing step—typically a band-pass filter, or no filtering at all—to every record regardless of its quality. Yet clinical ECG noise is heterogeneous: low-frequency baseline wander, electromyographic (EMG) activity, electrode-contact problems, and burst artifacts co-exist with essentially clean recordings [1,4]. A filter tuned for the noisy minority may remove diagnostically relevant morphology from the clean majority [5], while a light pipeline chosen for clean signals leaves the noisy minority under-treated. Deep classifiers are sensitive to such perturbations [6,7], and preprocessing choices measurably change their behavior [8].

Two requirements of trustworthy clinical machine learning sharpen this problem. First, aggregate discrimination hides stratification: a model can achieve a high average AUROC while failing on an identifiable subgroup [9], and signal quality is one of the most obvious strata. Second, decision support needs calibrated probabilities, not only correct rankings; modern neural networks are systematically over-confident and require post-hoc calibration [10,11], and confidence is least trustworthy for degraded or shifted ECG input [12]. Preprocessing, subgroup robustness, calibration, and computational cost are therefore coupled design variables, but they are seldom optimized jointly.

Physiological regulation offers a template for coupling them. In homeostasis, a sensor measures a regulated variable, a controller compares it with a set point, and an effector acts only as much as the measured deviation requires; this negative-feedback organization is regarded as the central organizing principle of physiology [13]. Homeostasis also makes empirical commitments: effector recruitment should be graded with the size of the disturbance, correction should be applied where the deviation is, the regulated variable should be held more stable than it would be without regulation, and the effort spent on regulation should be economical. These commitments can be translated into a preprocessing pipeline—a signal-quality sensor, a tier-gated controller, a bank of filtering operators and calibration gains as effectors—and, importantly, they can be tested. Bio-inspired adaptive encoding has recently been proposed for energy-efficient cardiac sensing in this journal [14], but, to the best of our knowledge, the preprocessing stage of a diagnostic 12-lead ECG pipeline has not been formulated as a regulatory loop whose biological predictions are explicitly checked.

Automatic ECG quality assessment is a mature field. Feature-based signal-quality indices (SQIs) capturing baseline power, high-frequency power, kurtosis, flat-line ratio, inter-lead consistency [15,16], fuzzy fusion [17], and learned estimators [18,19] are widely used, and in this journal, Kim et al. addressed class imbalance in an ECG quality classifier [20]. Systematic reviews stress that most SQI methods are validated on small, single-lead, curated sets [21], and PTB-XL’s own artifact annotations are incomplete [1]. SQIs have mostly been used to reject or flag records; using them to route records to different processing regimes is less common, although SQI-driven adaptive filtering with genetic-algorithm tuning has been reported for ambulatory ECG [22] and a recent test-time adaptation method gates self-training updates with an SQI [23]. Robustness studies have shown systematic degradation of CNN classifiers under physiological noise from the MIT-BIH Noise Stress Test Database [6,24,25], and learned denoisers such as score-based diffusion models have been proposed for baseline wander and muscle-noise removal [26]. These studies motivate robustness objectives but treat preprocessing as either fixed or a heavy learned model.

Temperature scaling [10] is the standard single-parameter calibrator; class-wise expected calibration error (ECE) [27] is the usual metric, with the caveat that binning choices influence conclusions [28]. Group distributionally robust optimization [29] and minimax-Pareto fairness [30] formalize worst-group objectives, and subgroup failures have been documented in ECG and other medical AI [9,31,32]. The worst-group idea is adopted at the level of technical quality rather than demographics, as one Pareto objective among four. Because the proliferation of metaphor-driven “novel” metaheuristics has drawn justified criticism [33], an established algorithm is deliberately used—NSGA-II [34] in its reference implementation [35]—and the bio-inspiration is located in the architecture of the regulated system, not in the optimizer; multi-objective selection of a single operating point follows current practice in machine-learning model selection [36].

The pre-registered research question (Supplementary S1) was whether, on PTB-XL v1.0.3, a homeostasis-inspired policy that selects a preprocessing operator and a calibration temperature per signal-quality tier can preserve diagnostic discrimination relative to fixed preprocessing of the same classifier while improving worst-tier performance and probability calibration on low-quality signals. The pre-registered answer is largely negative: the adaptive policy did not outperform the best fixed band-pass filter on discrimination, worst-tier performance, or calibration. This article is therefore framed as a rigorous negative study. Its contributions are:

1. An operational, testable formulation of homeostatic preprocessing. The sensor–controller–effector–set-point structure is mapped onto measurable components (Table 1), and four predictions that follow from the biological analogy are stated and tested (Section 3.14).
2. A leakage-controlled evaluation protocol for adaptive preprocessing. Patient-wise folds, validation-only selection, a pre-specified knee rule, ten optimizer seeds, an equal-budget random-search control, patient-clustered bootstrap with Holm correction, knee-stability analyses (bootstrap and patient-disjoint cross-fitting), and real recorded noise from the MIT-BIH Noise Stress Test Database.
3. Mechanistic explanations of the negative results. The post hoc analyses show why the adaptive policy ties the fixed filter (absence of a tier-dependent trade-off in the operator bank), why the worst-tier objective was not binding, why the quality sensor saturated, why equal-budget random search failed, and what the quality sensor costs end-to-end.
4. Falsifiable design principles for bio-inspired adaptive preprocessing that follow from these findings (Section 4.6), each stated with the observation that would refute it.

## 2. Materials and Methods

### 2.1. Study Design and Pre-Registration

The study followed a written protocol fixed before any test-fold evaluation (Supplementary S1). The protocol specified the dataset version, task, patient-wise split, classifier, quality-sensor features, operator bank, policy space, four objectives, optimizer budget, knee-selection rule, baselines and ablations, corruption scenarios, endpoints and statistical analysis, and an honesty rule: the test fold (fold 10) may be read only after every selection step is complete, no neutral or negative result may be suppressed, and any forced methodological change must be appended to the protocol with a date. One such deviation occurred before test access (Section 2.5).

The analyses that were not part of the protocol (Section 2.12, Section 2.13, Section 2.14, Section 2.15, Section 2.16 and Section 2.17) are labeled post hoc throughout. They reuse the locked artifacts of the pre-registered pipeline, never use fold 10 for any selection step, and are reported irrespective of outcome. To test reproducibility, the complete pipeline was executed a second time from the raw PTB-XL files with the released code and unchanged seeds (reproducibility run), and all reported numbers are taken from this run, so that every pre-registered and post hoc analysis uses the same networks. CPU non-determinism in network training produced small differences relative to the primary run (e.g., best epochs; test macro-AUROC of the baseline policies within 0.0013), but the knee selected by the search differed from the one locked in the primary run (Section 3.4). The policy locked in the primary run is evaluated exactly as locked.

### 2.2. Dataset and Task

PTB-XL v1.0.3 from PhysioNet [2,37] was used, with the 100 Hz waveforms and the official metadata files. A data audit confirmed 21,799 unique ECG records from 18,869 unique patients, a header and waveform file for every record (21,799 pairs), and a uniform 1000 × 12 signal structure. These counts correspond to version 1.0.3 and differ from the original data descriptor (21,837 records, 18,885 patients) because later releases removed duplicates [1,2]. Diagnostic labels were mapped to the five super-classes NORM, MI, STTC, CD, and HYP through scp_statements.csv; records with at least one diagnostic super-class label formed the analysis set of 21,388 records, formulated as multi-label classification with five sigmoid outputs.

### 2.3. Patient-Wise Split and Leakage Control

The recommended PTB-XL stratified folds were retained: folds 1 to 8 for training (17,084 records), fold 9 for validation (2146 records), and fold 10 as the locked test fold (2158 records) [2,3]. No patient appears in more than one partition. Per-lead standardization statistics were estimated from the training folds only. Class-wise decision thresholds, calibration temperatures, the Pareto knee, and every model-selection decision were fixed on fold 9 before fold 10 was read.

### 2.4. Classifier Backbone

The diagnostic model is a compact one-dimensional residual CNN receiving 12 × 1000 standardized samples. A stem convolution (24 channels, kernel 15, stride 4) is followed by four residual blocks with 24, 48, 96 and 128 channels, kernel sizes 9, 9, 7 and 5, strides 1, 2, 2 and 2, batch normalization and dropout increasing from 0.05 to 0.20; global average pooling and a dropout-regularized linear layer produce five logits (306,389 parameters). Training used class-weighted binary cross-entropy (positive weights clipped to [1,8]), AdamW (learning rate 10^−3^, weight decay 10^−3^), gradient-norm clipping at 5, batch size 128, ReduceLROnPlateau on validation macro-AUROC (factor 0.5, patience 2) and early stopping after six epochs without improvement (maximum 25 epochs), on CPU, with mild stochastic corruption augmentation (Gaussian noise with σ ≤ 0.08 in standardized units, *p* = 0.6; sinusoidal baseline oscillation of 0.15–0.5 Hz, *p* = 0.45; random lead masking, *p* = 0.2). The network was then frozen, so that all fixed and adaptive policies are evaluated on identical weights (Section 2.16 relaxes this). Three model seeds (20260901–20260903) were trained; policy search and evaluation used seed-averaged logits.

### 2.5. Quality Sensor: Weakly Supervised Technical Signal-Quality Estimator

For every record, 88 technical features are extracted: seven per-lead descriptors for each of the 12 leads (relative baseline power in 0.1–0.7 Hz, relative high-frequency power in 35–50 Hz, robust first-difference ratio, flat-line fraction, robust-outlier fraction, spectral entropy and maximum normalized jump) and four cross-lead descriptors (Einthoven and Goldberger consistency errors, median lead scale and coefficient of variation of lead scales), robustly standardized with statistics from folds 1 to 7.

Protocol deviation (1 September 2026, before test access). The protocol originally specified a fully unsupervised composite SQI. On folds 1 to 9, this score separated the PTB-XL artifact_any indicator (union of baseline_drift, static_noise, burst_noise and electrodes_problems) with a ROC-AUC of only 0.516, and its tiers differed markedly in pathology prevalence (Section 3.2). The estimator was therefore revised to an ExtraTrees classifier (500 trees, depth 14, minimum leaf size 5, balanced class weights) predicting artifact_any from the 88 features, fitted on folds 1 to 7. Tier boundaries were the tertiles of the predicted score on fold 8 (high, mid, low quality). artifact_any is a noisy, partial annotation [1]; tier membership is an estimate of technical-artifact probability, not of clinical quality. Under corruption, the sensor is re-applied to the corrupted waveform, so tier assignment responds to the disturbance.

### 2.6. Effector: Deterministic Operator Bank and Cost Proxy

Six deterministic operators span the range from no intervention to aggressive smoothing: identity (P0); robust linear detrend (P1); zero-phase third-order Butterworth band-pass filters at 0.5–40 Hz (P2), 0.5–35 Hz (P3) and 1–40 Hz (P4), implemented as forward–backward second-order sections; and Savitzky–Golay trend removal (window 101, order 2) followed by light smoothing (window 7, order 2) (P5). Computational cost was defined as CPU preprocessing latency per record, measured on a single machine. Across the three seed runs, the median latencies for P0–P5 were 0.045, 0.465, 0.309, 0.305, 0.293, and 0.750 ms/record, respectively; these values were min–max normalized for optimization. The computational costs of the quality sensor and CNN inference are reported separately in Section 2.17. As pre-registered, the optimization objective J_4_ includes the operator cost only.

The second component of the effector is a tier-specific temperature T_q_ applied to the logits, z/T_q_, before the sigmoid [10]. Temperatures below one sharpen and above one soften predicted probabilities; because confidence is least trustworthy on degraded input [12], a separate temperature per tier lets calibration differ between quality strata.

### 2.7. Controller: Homeostatic Policy Formulation

Let q(x) ∈ {high, mid, low} denote the tier assigned to record x by the sensor. A policy is the vector

θ = [*p_high_*, *p_mid_*, *p_low_*, *T_high_*, *T_mid_*, *T_low_*], *p_q_* ∈ {0,…,5}, T_q_ ∈ [0.5, 3.0] (1)

that assigns one operator and one temperature to each tier. The regulated output for a record is

(2) $$\hat{y}\left(x;\;\theta \right)=\sigma \left(\frac{f\left(\;P_{\left\{p_{\left\{q\left(x\right)\right\}}\right\}}\left(x\right)\;\right)}{T_{\left\{q\left(x\right)\right\}}}\right)$$

where f is the frozen classifier, and P_k_ is the k-th operator. Table 1 makes the correspondence with the biological loop explicit. The final column identifies the empirical commitment implied by each element of the analogy and is evaluated in Section 3.14.

**Figure 1:**
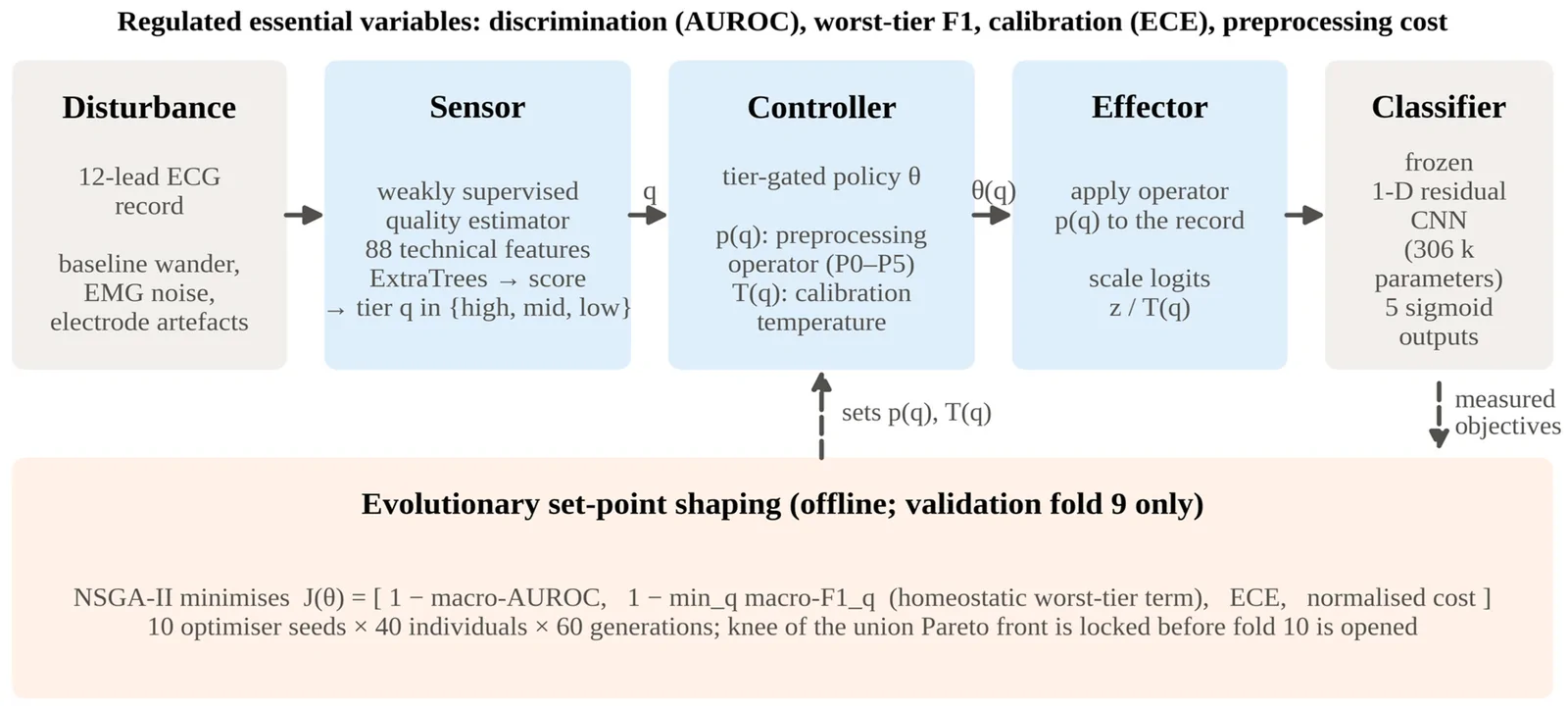
The proposed pipeline as a homeostatic control loop.

Figure 1 shows the proposed pipeline as a homeostatic control loop.

Class-wise decision thresholds were selected on fold 9 by maximizing per-class F1 for identity-operator predictions [38] and were held constant across all policies and scenarios. The resulting thresholds were 0.49 for NORM, 0.68 for MI, 0.59 for STTC, 0.56 for CD, and 0.78 for HYP. The optimizer minimizes four objectives on fold 9:

*J*_1_(*θ*) = 1 − *MacroAUROC*(*θ*), *J*_2_(*θ*) = 1 − *min_q_MacroF*1*_q_*(*θ*), *J*_3_(*θ*) = *ECE*(*θ*), *J*_4_(*θ*) = *Σ_q_π_q_* · *c*(*p_q_*) (3)

where π_q_ is the tier prevalence and c(·) is the normalized operator cost. J_2_ is the homeostatic set-point term, a minimax objective over quality tiers in the spirit of group distributionally robust optimization [29] and minimax-Pareto fairness [30].

### 2.8. Evolutionary Search and Policy Locking

NSGA-II [34] was run with a population of 40 for 60 generations for ten optimizer seeds (20260911–20260920), i.e., 24,000 policy evaluations, using pymoo [35] with duplicate elimination; integer operator indices were obtained by rounding. Logits for every operator were pre-computed once for fold 9 (an operator bank), so evaluating a policy amounts to selecting rows and applying temperatures. The non-dominated sets of the ten runs were pooled, and the knee—the point with minimum Euclidean distance to the ideal point after min–max normalization of each objective over the pooled set—was selected and written to a locked file before test access.

### 2.9. Baselines and Ablations

Policies evaluated on identical frozen weights: (B0) identity without calibration (T = 1); (B1) identity with a global temperature minimizing fold-9 ECE; (B2) the best fixed operator by fold-9 macro-AUROC with its own global temperature—the strongest conventional pipeline and the main comparator; (B3) the knee of an equal-budget random search (24,000 evaluations, same rule); (A1) NSGA-II without J_2_; (A2) the pre-registered routing with all temperatures set to 1. Three variants of the proposed policy are evaluated to distinguish the pre-registered configuration from calibration and optimizer-selected alternatives. *P*-locked is the policy locked before test access and designated as the pre-registered primary policy, with routing P0/P0/P2 and tier temperatures T = 0.848/0.905/0.832. *P*-refit retains this routing but estimates tier-specific temperatures on fold 9 by minimizing ECE, following the procedure used for B1/B2. *P*-rerun is the knee solution selected by the multi-objective search on fold 9.

### 2.10. Controlled Corruption Scenarios

Two deterministic synthetic corruption families were applied to fold 10. Baseline wander added, per lead, a sinusoid of 0.15–0.45 Hz, random phase and amplitude proportional to the lead’s robust scale (severities 0.5, 1.0, 1.5); EMG-like noise added 20–45 Hz band-limited Gaussian noise at SNRs of 20, 10, and 5 dB. Random parameters were seeded by record identifier, so every policy saw identical corrupted samples. Severities of 1.5 and 5 dB lie outside the training augmentation range. Real recorded noise was added in Section 2.15.

### 2.11. Endpoints and Statistical Analysis

Primary endpoints were test macro-AUROC, worst-tier macro-F1, and class-wise ECE, calculated using 15 equal-width bins and averaged across classes [27]. Secondary endpoints were macro-F1, per-class metrics, the Brier score, and preprocessing latency. Differences between policies were estimated using a patient-clustered paired bootstrap with 1000 patient resamples, with both policies evaluated on the same resamples. The mean difference, the 2.5th–97.5th percentile interval, and a two-sided bootstrap *p*-value are reported. Holm’s step-down correction was applied separately to two comparison families: the pre-registered family, comprising *P*-locked versus B1, B2, B3, and A1 across the three pre-registered scenarios and three metrics (36 tests), and the post hoc family, comprising all remaining comparisons. Differences were considered statistically significant only when the Holm-adjusted *p*-value was <0.05; intervals are reported throughout.

### 2.12. Post Hoc Analysis 1: Tier Composition and Morphological Fidelity

To test whether quality tiers are confounded with pathology, we reported the prevalence of every diagnostic super-class, the annotated artifact rate, age, and sex by tier on folds 9 and 10, with the strength of association measured by Cramér’s V (χ^2^ test). The same table is reported for the abandoned unsupervised SQI. To test whether macro-AUROC hides localized morphological damage, we measured that, on every native test record, three clinically used amplitudes on the lead-wise median beat (R peaks detected on lead II) before and after each operator: the ST level (mean amplitude 80–100 ms after the R peak minus the PR baseline 120–200 ms before it), the Q-wave depth (minimum 10–60 ms before the R peak relative to baseline) and the R amplitude. It is reported that the median and 95th percentile of the absolute change, along with the proportion of lead–record pairs with a change of ≥50 µV in ST level or Q depth, were calculated; 50 µV corresponds to half the 0.1 mV criterion used for ST deviation [5]. Discrimination was additionally reported at the diagnostic sub-class level (e.g., anterior, inferior and lateral MI; ischemic ST-T change) by computing the AUROC of the parent super-class score for records with the sub-class against records without the parent super-class, and as sensitivity at 90% specificity per super-class.

### 2.13. Post Hoc Analysis 2: Expanded Effector Bank and Disturbance-Rich Validation

The pre-registered bank (P0–P5) contained no operator whose benefit on noisy records is paid for by damage to clean ones. Three operators were therefore added: P6, the 0.5–40 Hz band-pass followed by Daubechies-4 wavelet soft-threshold denoising of the two finest detail levels (universal threshold); P7, an aggressive 0.5–20 Hz zero-phase band-pass that strongly suppresses EMG at the price of QRS attenuation; and P8, two-stage median-filter baseline removal (windows of 21 and 61 samples). Because native fold 9 contains little disturbance, the policy search was repeated in two validation regimes: native (fold 9, as pre-registered) and disturbed (fold 9 plus copies of the same records with baseline wander of severity 0.75 and EMG-like noise at 15 dB; these levels are not used in any test bench, and the sensor is re-applied to the disturbed copies). In each regime, NSGA-II (five seeds, population 40, 60 generations) was run with and without the worst-tier objective J_2_. J_2_ was considered binding if removing it changed the operators of the selected knee; the conflict between average and worst-tier performance along the front was summarized by Spearman’s ρ between J_1_ and J_2_. The disturbed-regime knee was compared on all test benches against the calibrated identity pipeline, the best fixed operator of each regime, and the locked pre-registered policy.

### 2.14. Post Hoc Analysis 3: Re-Calibrated and Lightweight Quality Sensors

The pre-registered sensor saturated under EMG-like noise because no training record contained broadband noise of that magnitude. A re-calibrated sensor was trained with the same 88 features and ExtraTrees settings on folds 1 to 7, augmented with one corrupted copy of every training record (baseline wander of severity U(0.2, 2.0) or EMG-like noise at SNR U(0, 35) dB, chosen at random); a corrupted copy was labeled as artifact if its severity was ≥1.0 or its SNR ≤ 12 dB, otherwise it inherited the record’s PTB-XL artifact label. To address the cost of the sensor itself, a lightweight sensor was trained on 36 features (per lead: log baseline-band power ratio, log high-band power ratio, and flat-line fraction) with an L2-regularized logistic regression. Tier edges for both sensors were the fold-8 tertiles, as pre-registered. Routing behavior and the locked policy’s performance were evaluated with each sensor on every test bench.

### 2.15. Post Hoc Analysis 4: Real Recorded Noise and the Full Severity Gradient

In addition to the synthetic noise benches (Section 2.10), the test fold was evaluated under recorded-noise conditions using the MIT-BIH Noise Stress Test Database (NSTDB) [24,37]. Baseline-wander (bw), muscle-artifact (ma), and electrode-motion (em) recordings were resampled from 360 Hz to 100 Hz. For each lead, a noise channel and a 10 s window were selected at random using a random-number generator seeded by the record identifier, then scaled to achieve per-lead SNRs of 12, 6, or 0 dB. Together with the native test fold and the six synthetic benches, these conditions yielded 16 test scenarios, all reported in Section 3.6; all metrics are provided in Supplementary Table S1. No independent ECG dataset was evaluated. Thus, the NSTDB experiments assess robustness to externally recorded noise added to PTB-XL test records, but do not constitute cross-dataset validation.

### 2.16. Post Hoc Analysis 5: Classifier Adapted to the Policy

The pre-registered design froze the classifier so that policies could be compared on identical weights. To test whether the adaptive policy would help a network that has learned under it, each of the three seed models was fine-tuned for up to five epochs (AdamW, learning rate 10^−4^, the same class weights and augmentation) on folds 1 to 8 under three input conditions: the locked adaptive routing (low-tier records filtered with P2, others unfiltered; FT-policy), P2 for every record (FT-P2) and unfiltered input (FT-identity, which controls for the additional training epochs). The epoch with the best validation macro-AUROC under the same condition was kept; temperatures (per tier for FT-policy) and class thresholds were refitted on fold 9 for every pipeline, including the frozen-model pipelines used as comparators in this analysis.

### 2.17. Post Hoc Analysis 6: Search Diagnostics, Knee Stability and End-to-End Cost

The number of distinct operator triples visited among the 216 possible triples during the 24,000 random evaluations was reported, together with the objective ranges observed by the knee-selection rule; the knee obtained when the rule was applied to the non-dominated subset of the random sample rather than to the full sample; the hypervolume values for both searches [39], calculated using a common normalization and a reference point of 1.1 for each objective; and the anytime hypervolume curves.

Because selection based on a single validation fold may be unstable [40], stability was assessed in three ways: (i) by examining the knee selected for each of the ten NSGA-II seeds; (ii) by calculating how frequently each operator triple was selected across 200 bootstrap resamples of fold-9 patients, with the front rescored and the knee re-selected for each resample; and (iii) by patient-disjoint split-half cross-fitting, in which the knee selected by NSGA-II on one-half was scored on the other half, and vice versa. Full nested cross-validation across folds 1 to 9 was not performed because it would have required retraining the classifier within each outer fold and was not feasible. Split-half cross-fitting was therefore used as the closest feasible approximation.

Per-record latency was measured on the same CPU for each pipeline component—sensor feature extraction, sensor-model inference, operator execution, and CNN inference—in batched (1024 records) and streaming (single-record) modes. Total pipeline latency was reported for the identity, fixed-P2, and adaptive pipelines with each sensor. Energy was not measured (Section 4.7).

### 2.18. Software, Code Availability and Use of Generative AI

Experiments used Python 3.11 with NumPy 2.4, SciPy 1.17, scikit-learn 1.8, PyTorch 2.14 (CPU), pymoo 0.6.2, PyWavelets 1.8, wfdb 4.3 and pandas 3.0; figures were produced with Matplotlib 3.10. All random seeds (model training, optimization, random search, corruption, NSTDB window selection, and bootstrap) are fixed in the released code (Code S1). Generative-AI tools were used for code drafting, literature retrieval assistance, and language editing under the author’s supervision; all analyses, numerical results, and interpretations were verified by the author, who assumes full responsibility for the content.

## 3. Results

### 3.1. Data Audit and Baseline Classifier

The dataset audit confirmed the official PTB-XL v1.0.3 counts (21,799 records, 18,869 patients, and 21,388 diagnostic records), with no patient shared across partitions. For the three classifier seeds, validation macro-AUROC values of 0.9178, 0.9190, and 0.9175 were obtained at epochs 9, 8, and 12, respectively (mean 0.9181 ± 0.0008). Per-class AUROC ranged from 0.896 for HYP to 0.940 for NORM (Table 2). For context, the PTB-XL benchmark reports super-class macro-AUROC values of 0.921–0.930 for considerably larger networks [3]. The backbone was deliberately kept compact (0.31 M parameters) and trained on a CPU so that the analysis could focus on the preprocessing policy.

### 3.2. Construct Validity of the Quality Sensor and Tier Composition

The weakly supervised estimator separated annotated artifact records with ROC-AUC 0.760 on fold 9 and 0.762 on fold 10 (average precision 0.515; artifact prevalence 24.6% and 23.8%). Tier sizes were 663/725/758 (validation) and 699/737/722 (test). Table 3 reports the diagnostic composition of every tier. Annotated artifact prevalence rises steeply from the high- to the low-quality tier (test: 7.3%, 19.0%, 44.6%; Cramér’s V = 0.36, *p* < 10^−60^), whereas the association with diagnostic class is weak: V ≤ 0.11 for every super-class, and non-significant for MI, STTC and HYP on both folds. A residual gradient remains—NORM prevalence is lower and CD prevalence higher outside the high-quality tier (test NORM 49.8% vs. 42.2%; V = 0.07), and median age rises from 61 to 65 years—consistent with older and conduction-disturbed patients producing noisier recordings rather than with the sensor detecting pathology. The abandoned unsupervised SQI shows the opposite pattern: no association with artifacts (V = 0.02, *p* = 0.55) but associations with every diagnostic class (V = 0.08–0.20, all *p* < 0.001), which is the quantitative basis of the pre-registered protocol deviation.

### 3.3. Effect of Individual Operators on Discrimination and on Waveform Morphology

Table 4 reports, for the seed-averaged classifier, the validation macro-AUROC of each operator applied to all records, its cost, and its effect on clinically used amplitudes of the native test records. Mild band-pass filtering was marginally better than no filtering (P2 0.9274 vs. P0 0.9263) and essentially indistinguishable from P3 (0.9273); the 1 Hz high-pass (P4) and Savitzky–Golay smoothing (P5) were harmful. The morphology analysis explains why P2 costs so little on clean records: it changed the ST level by ≥50 µV in only 1.9% of lead-records (2.4% in MI and STTC records) and the Q-wave depth by ≥50 µV in 3.2%, with a median R-amplitude change of 3.4%. The 1 Hz high-pass P4 shifted the ST level by ≥50 µV in 14.3% of lead-records, as expected from its cut-off above the 0.67 Hz limit recommended for ST fidelity [5], and the new aggressive P7 (0.5–20 Hz) altered Q depth by ≥50 µV in 34.6% of lead-records and attenuated R amplitude by a median 27.6%. Thus, the pre-registered bank contained no operator that is clearly beneficial on noisy records yet clearly damaging on clean ones; P7 was added to provide one (Section 3.9).

### 3.4. The Locked Homeostatic Policy and Its Reproducibility

**Figure 2:**
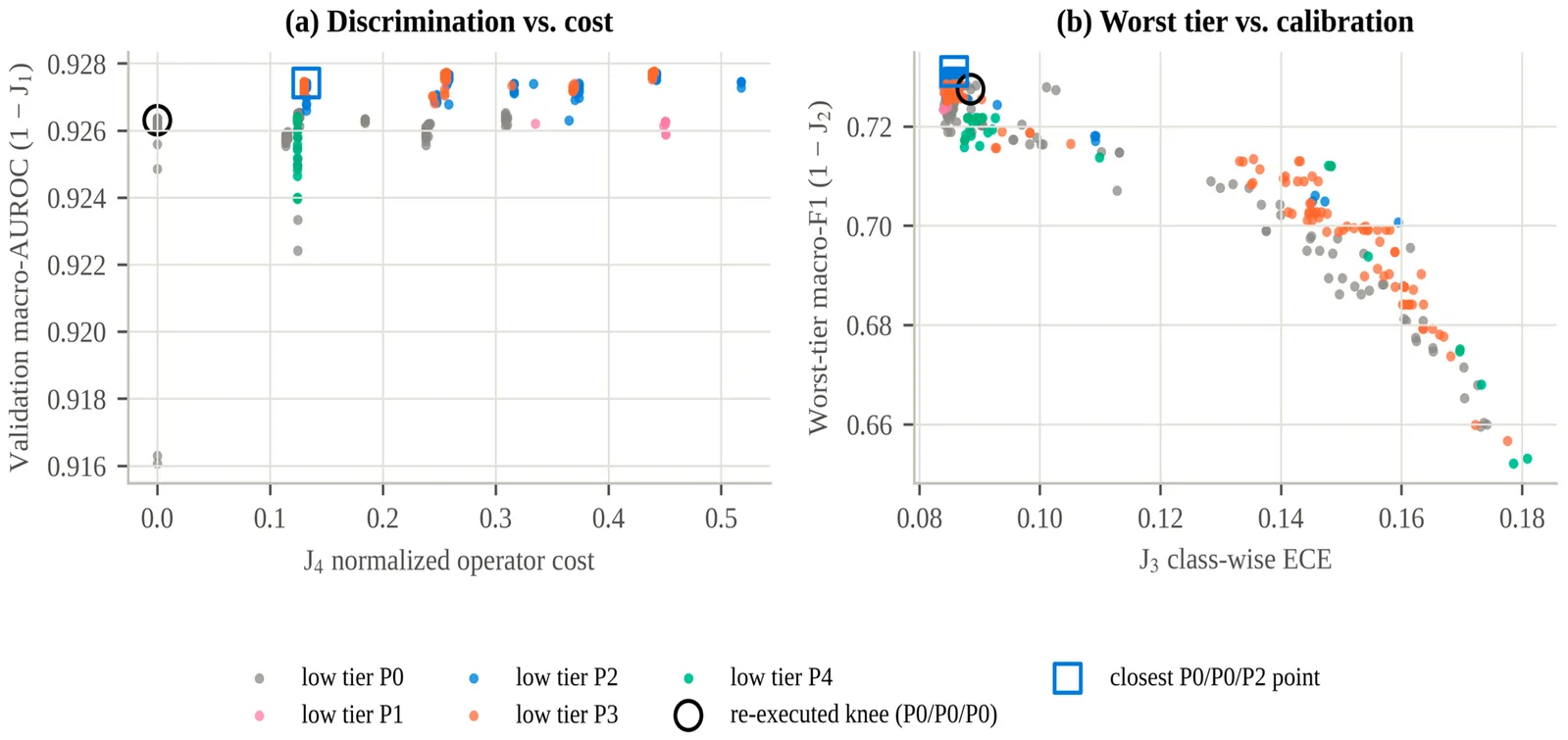
Union Pareto front of the ten NSGA-II runs on the validation fold (382 policies), colored by the operator assigned to the low-quality tier. (**a**) Validation macro-AUROC against normalized operator cost; (**b**) worst-tier macro-F1 against class-wise ECE. (Circle: knee selected by the pre-registered rule; square: closest policy with the pre-registered routing P0/P0/P2.).

The policy locked before test access in the primary run assigned identity to the high- and mid-quality tiers and P2 to the low-quality tier (temperatures 0.848, 0.905, 0.832). In the reproducibility run, the pooled front of the ten NSGA-II runs contained 382 non-dominated policies (Figure 2), and the knee rule selected identity processing for all three tiers (P0/P0/P0; temperatures 1.004, 1.056, 1.014). The pre-registered routing P0/P0/P2 was still on the front and was the best policy on worst-tier F1 (validation 0.731) and close to best on macro-AUROC (0.9274), but it lay farther from the normalized ideal point (distance 0.257 vs. 0.140) because its cost (J_4_ = 0.13) is large relative to the cost range (0.52), whereas its gains in J_1_ and J_2_ are small relative to their ranges (0.012 and 0.079). The selected operators therefore depend on minute differences in discrimination that are of the same order as between-seed variation of the classifier. Section 3.10 quantifies this instability.

### 3.5. Native Test Performance

Table 5 summarizes performance on the locked test fold. B2 had the highest macro-AUROC of all policies (0.9231). P-locked achieved a macro-AUROC of 0.9225 (bootstrap difference vs. B2: −0.0007; 95% CI, −0.0015 to 0.0002), a worst-tier macro-F1 of 0.7254 versus 0.7298, and a class-wise ECE of 0.0839 versus 0.0837, with 57% lower operator cost (0.133 vs. 0.309 ms/record). None of the 12 native-fold differences between P-locked and its four comparators was significant after Holm adjustment (Table 6). The ECE results did not establish a distinct advantage for the adaptive policy: the observed differences were small relative to their confidence intervals and to the variation across the individual model seeds comprising the ensemble (single-seed native ECE, 0.073–0.112). P-search selected the same operators as identity processing; both had the lowest worst-tier macro-F1.

Per-class results (Supplementary Table S2) show that *P-locked* and B2 are within 0.002 AUROC for every class. The largest class-wise ECE was observed for HYP and MI under every policy; filtering reduced ECE for STTC (B1 0.090, B2 0.076, *P-locked* 0.083) but increased it for MI (0.109, 0.134, 0.118).

### 3.6. Full Severity Gradient: Synthetic and Real Recorded Noise

**Figure 3:**
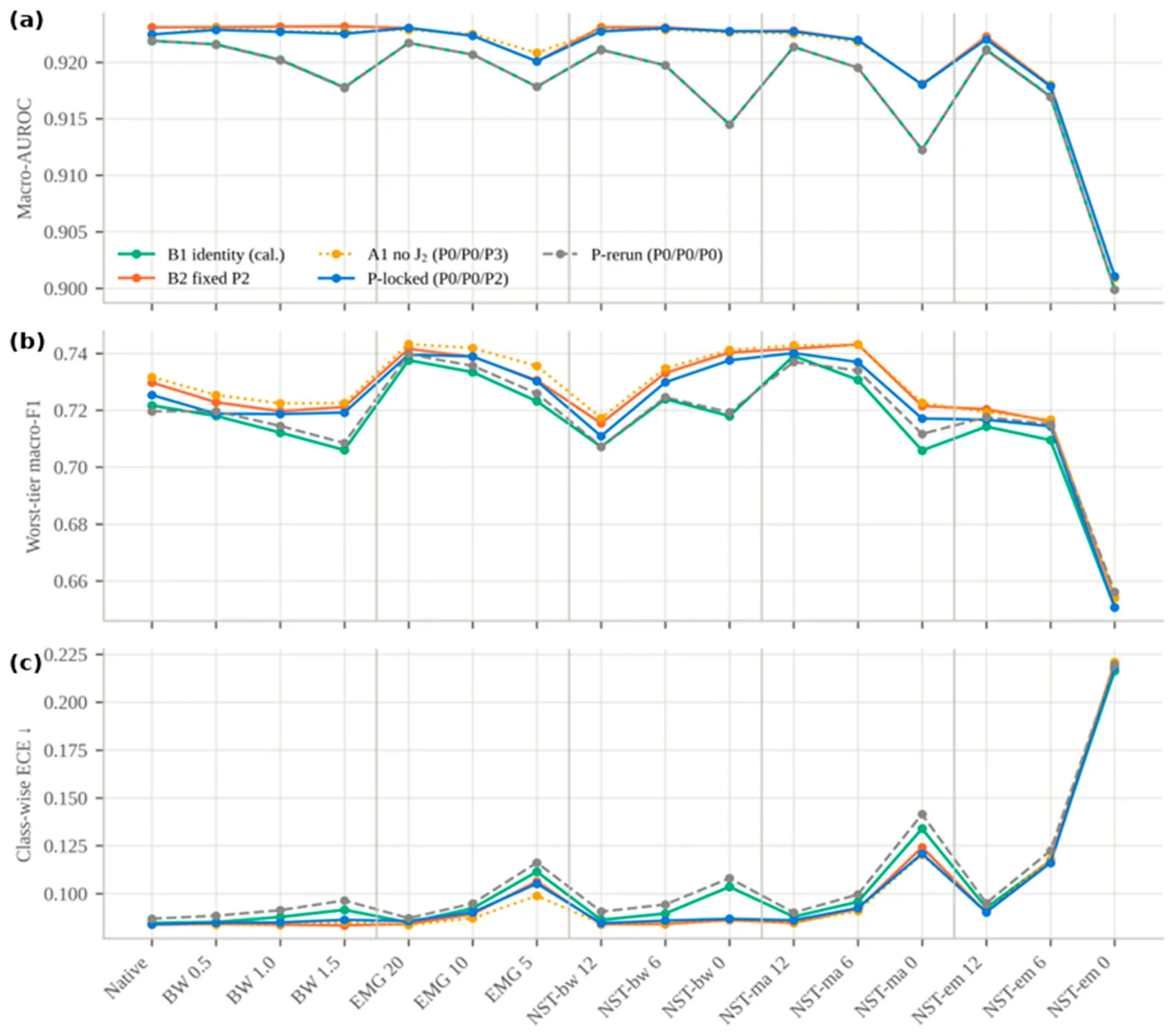
Full severity gradient on the locked test fold for the principal policies: (**a**) macro-AUROC, (**b**) worst-tier macro-F1 (tiers with <50 records not scored), (**c**) class-wise ECE. BW and EMG: synthetic baseline wander (severity) and band-limited EMG-like noise (SNR, dB); NST-bw/ma/em: real baseline wander, muscle artifact, and electrode-motion recordings from the MIT-BIH Noise Stress Test Database at 12, 6, and 0 dB.

Figure 3 shows all 16 test benches for the principal policies; every number is listed in Supplementary Table S1. Three regularities hold across the gradient. First, unfiltered processing (B1 and P-rerun, which routes identically) degrades monotonically with baseline-wander severity (macro-AUROC 0.9219 → 0.9178 at synthetic severity 1.5 and → 0.9145 for NSTDB baseline wander at 0 dB), whereas every policy that filters the disturbed records stays within 0.001 of its native value. Second, under recorded muscle artifact and electrode motion, all policies lose discrimination (NSTDB at 0 dB: 0.900–0.901 for every policy), i.e., neither filtering nor routing compensates for motion artifact, which overlaps the ECG spectrum. Third, P-locked tracks the fixed filter B2 throughout: its macro-AUROC was never higher than B2’s by more than 0.0001, and the sensor routed between 70% and 100% of records to the filtered tier in every corrupted bench, so that under heavy noise the two pipelines become operationally identical (Section 3.7).

### 3.7. Paired Bootstrap Comparisons with Holm Adjustment

Table 6 lists the pre-registered family (P-locked versus four comparators in the three pre-registered scenarios), now with Holm-adjusted *p*-values. After adjustment, P-locked was better than calibrated identity processing and the random-search knee in macro-AUROC under severe baseline wander (+0.0047, 95% CI 0.0028–0.0065) and EMG at 5 dB (+0.0023, 0.0013–0.0032), and in ECE against the random-search knee in both scenarios and against identity under EMG (all p_Holm < 0.001). Against the best fixed filter, no difference survived adjustment in any scenario or metric; the only nominally significant difference was in favor of the fixed filter (BW 1.5, ΔAUROC −0.0007, −0.0012 to −0.0001, *p* = 0.020, p_Holm = 0.44). Against the ablation without J_2_ (A1), P-locked was significantly worse under EMG at 5 dB (ΔAUROC −0.0007; ΔECE +0.0059; p_Holm ≤ 0.006), because A1 routed low-tier records to the slightly stronger low-pass P3.

In the post hoc family (13 further benches; Supplementary Table S4), P-locked was never significantly better than B2 in macro-AUROC, and better in ECE only at NSTDB electrode motion 0 dB (−0.0022, −0.0029 to −0.0014, p_Holm < 0.001); its ECE was numerically higher than B2’s in 10 of 13 benches. P-refit (refitted temperatures) was likewise indistinguishable from B2 in all 16 benches (0/32 significant AUROC/ECE differences), and P-rerun was significantly worse than B2 in AUROC in 7 and in ECE in 9 of 16 benches.

### 3.8. Sensor Saturation and Recalibration

**Figure 4:**
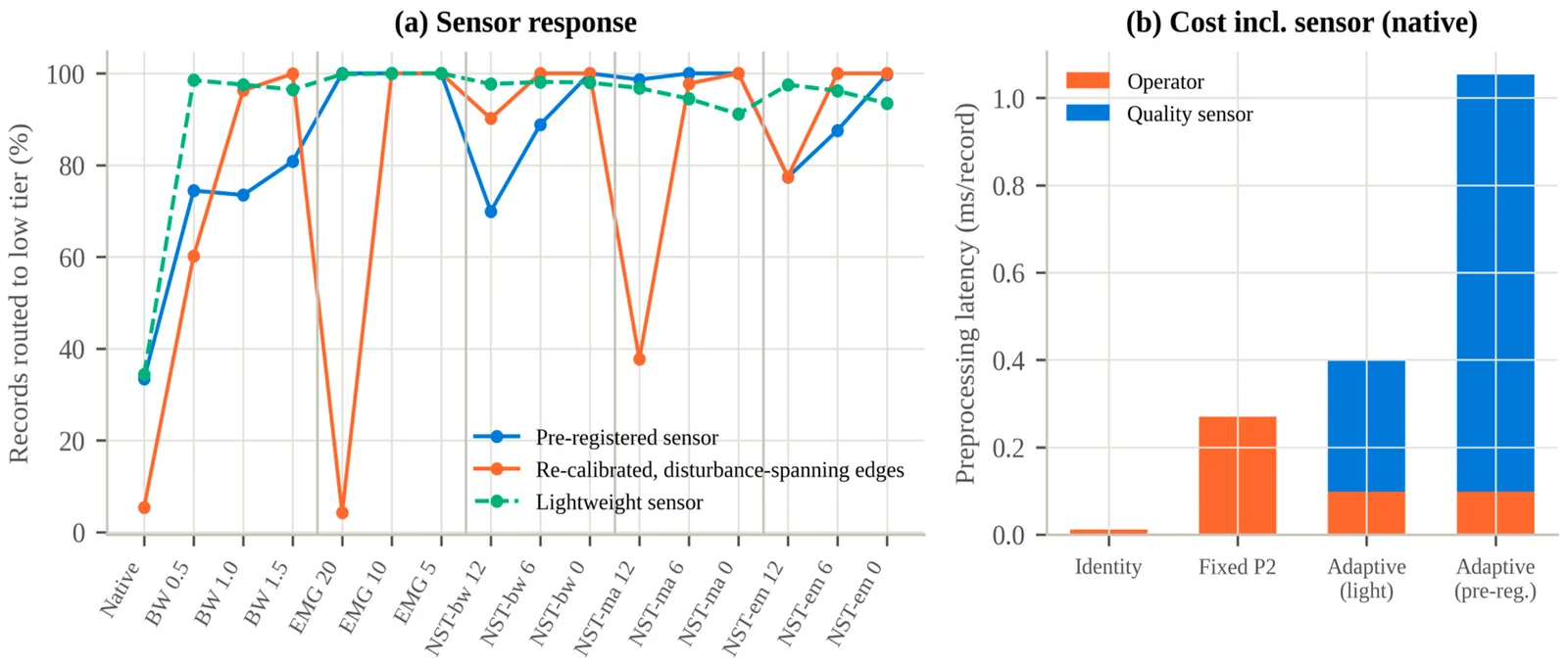
(**a**) Share of test records routed to the low-quality (filtered) tier in each of the 16 benches for the pre-registered, the re-calibrated (disturbance-spanning tier edges) and the lightweight sensor. (**b**) Preprocessing latency per record on native records (batched mode), split into operator and sensor cost.

With the pre-registered sensor (Figure 4a), the share of records routed to the low-quality tier was 33.5% on native records, 70–89% under recorded baseline wander and electrode motion at 12 and 6 dB, and 99–100% under every synthetic EMG level, under recorded muscle artifact at every SNR, and under all 0 dB benches (Supplementary Table S5). Retraining the sensor on corrupted copies of the training records (Section 2.14) changed its discrimination of annotated artifacts on native fold 9 very little (ROC-AUC 0.761 vs. 0.760). It also barely changed routing: 80% of records at EMG 20 dB and 99–100% under every baseline-wander level, because tier edges defined on native records still placed every disturbed record in the top tertile. Defining the edges on the validation disturbance mixture removed the saturation. The re-calibrated sensor then routed 5.4% of native records, 4.3% at EMG 20 dB and 100% at 10 and 5 dB, and 60%, 96% and 100% at baseline-wander severities 0.5, 1.0 and 1.5. The lightweight sensor (ROC-AUC 0.707; low-tier agreement with the pre-registered sensor 81%) saturated at 91–100% in every corrupted bench. Across sensors, test macro-AUROC of the pre-registered routing differed by at most 0.0014 in any bench (Supplementary Table S5), and the re-calibrated sensor did not improve on the pre-registered one in any bench by more than 0.0004 AUROC. Sensor precision therefore had almost no leverage on performance in this operator bank.

### 3.9. Expanded Effector Bank and the Worst-Tier Objective

In the validation profile of the expanded bank (Table 4 and Table S3), wavelet denoising (P6) was the best single operator on native fold 9 (macro-AUROC 0.9275). It was, however, fragile on test: under EMG at 5 dB, its macro-AUROC fell to 0.8962, against 0.9201 for P2. In the disturbed validation regime, the best single operator was P2. The aggressive low-pass P7 was the worst non-smoothing operator in every validation tier and regime (e.g., low-tier macro-F1 0.716 vs. 0.733 for identity in the disturbed regime). It therefore damaged clean records, as intended, but did not repay this on the disturbed ones: the EMG-like and recorded noises used here are already largely removed by the 40 Hz cut-off of P2. No knee in either regime selected P6, P7, or P8. With J_2_ the knees were P1/P3/P2 (native) and P0/P2/P2 (disturbed); without J_2_ they were P0/P2/P3 and P1/P2/P3. Removing J_2_ therefore changed the operators in both regimes. Along the fronts, J_1_ and J_2_ were weakly anti-correlated in the native regime (Spearman ρ = −0.08; pre-registered bank −0.10) and more strongly in the disturbed regime (ρ = −0.30), i.e., disturbance created some conflict between average and worst-tier performance. Two qualifications apply. First, the inclusion of the expensive P8 (9.8 ms/record) compressed the normalized cost of all other operators, so cost no longer restrained filtering, and the knees moved toward filtering the mid-quality tier as well. Second, given the knee instability documented in Section 3.10, a change of operators is weak evidence of a systematic effect. On test, the disturbed-regime knee with J_2_ was never better than always-P2 in macro-AUROC. It was worse at baseline-wander severities 0.5 and 1.5 and at recorded electrode motion at 12 and 6 dB (by 0.0004–0.0006; CIs excluding zero, unadjusted), and better in ECE only under recorded muscle artifact and electrode motion at 0 dB (−0.0024 and −0.0013).

### 3.10. Why Random Search “Failed”, and How Stable Is the Knee?

**Figure 5:**
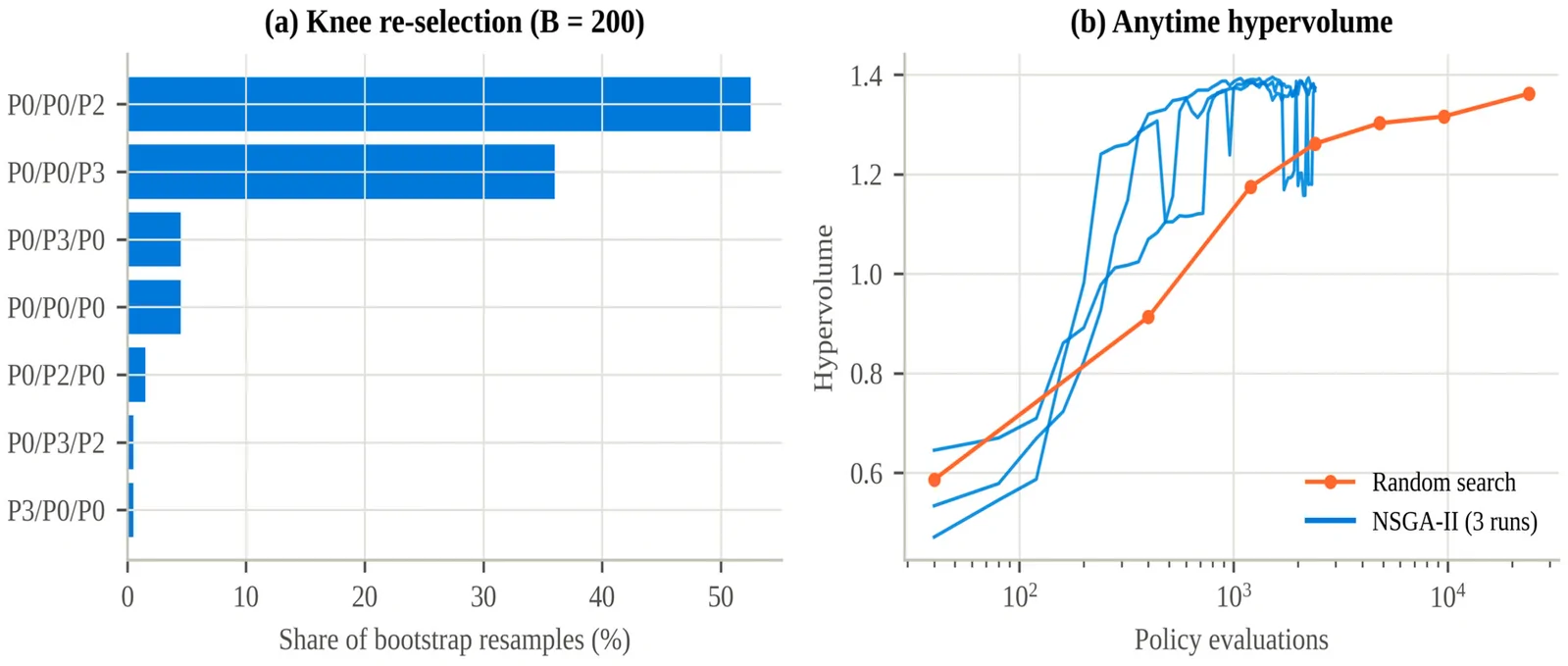
Stability of policy selection (validation fold). (**a**) Operator triple chosen by the knee rule in 200 patient-level bootstrap resamples. (**b**) Anytime hypervolume of three NSGA-II runs and of the equal-budget random search (common normalization).

The equal-budget random search, a strong baseline for configuration search [41], did not fail to explore: its 24,000 samples visited all 216 operator triples, and its non-dominated subset (47 policies) reached a hypervolume of 1.362 versus 1.403 for the pooled NSGA-II front (common normalization). NSGA-II was, however, about ten times more sample-efficient: a single NSGA-II run reached a hypervolume of 1.37 after 2400 evaluations, a value random search had not reached after 9600 (1.316) (Figure 5b). The random-search knee was identity processing for all tiers, whether the knee rule was applied to all 24,000 samples or only to their non-dominated subset, and it coincides in operators with the NSGA-II knee of the reproducibility run (Table 5). The apparent failure of random search in the primary run, whose knee was also identity processing, was therefore a property of the selection rule, not of the search: with min–max normalization, the cheapest policy is pulled toward the ideal point whenever cost spans a wide range and the accuracy objectives a narrow one, and the range seen by the rule was wider for the random sample (J_1_ range 0.046 vs. 0.012 on the NSGA-II front).

Table 7 shows that the operator choice is unstable at the level of individual operators but stable at the level of the regulatory structure. The knees of the ten individual NSGA-II runs were P0/P0/P2 in six runs, P0/P0/P0 in three, and P0/P0/P3 in one. When the fold-9 patients were bootstrap-resampled and the knee re-selected, P0/P0/P2 was chosen in 52.5% of 200 resamples, P0/P0/P3 in 36.0% and P0/P0/P0 in only 4.5%; in 88.5% of resamples the selected policy filtered the low-quality tier only, and the high-quality tier was left unfiltered in 99.5%. Temperatures varied with an interquartile range of 0.01–0.05. In patient-disjoint cross-fitting, the knee selected on one-half was P0/P0/P3 (A→B) and P0/P0/P2 (B→A); both reached test macro-AUROC 0.9224, and their held-out-half objectives were within 0.004 (J_1_) and 0.014 (J_2_) of the in-sample values. The pooled-front knee of the reproducibility run (P0/P0/P0) is thus an unlikely selection; the pre-registered P0/P0/P2 is the modal one. The practical lesson is that the qualitative homeostatic prescription (leave trusted signals unfiltered, filter the disturbed tier) is robust to sampling variation, whereas the specific operator and temperatures are not, and reported knees should be accompanied by such stability estimates.

### 3.11. Classifier Adapted to the Policy

Fine-tuning improved validation macro-AUROC by 0.001–0.003 in two of the three seed models under every condition (best epoch 1). The third model did not improve and was kept unchanged. Table 8 shows the test results. After fine-tuning, the network trained under the adaptive routing (FT-policy) reached the same discrimination as the network fine-tuned on always-filtered input (FT-P2) in 13 of 16 benches. It was worse on native records (ΔAUROC −0.0010, 95% CI −0.0019 to −0.0001) and marginally better under EMG at 5 dB and recorded muscle artifact at 6 dB (+0.0002 each). Its ECE was higher than FT-P2’s under recorded muscle artifact at 0 dB and electrode motion at 6 and 0 dB (+0.0020 to +0.0030), and not different elsewhere. All three fine-tuned pipelines had markedly lower ECE than their frozen counterparts (e.g., native: 0.068 vs. 0.085), an effect of the additional low-learning-rate epoch that was shared by the unfiltered control (FT-identity, 0.068). Training the classifier under the adaptive policy therefore did not create an advantage for the policy: the highest macro-AUROC in every bench was reached by a fine-tuned network with either always-on filtering or the adaptive routing, which were practically indistinguishable.

### 3.12. End-to-End Cost

Table 9 and Figure 4b report the latency of each pipeline component on the same 2-core CPU. In batched mode (1024 records), extracting the 88 sensor features took 0.831 ms/record and the 500-tree ensemble 0.125 ms/record. The P2 filter took 0.270 ms/record, identity 0.012 ms/record, and CNN inference 0.462 ms/record. The pre-registered sensor alone therefore costs 3.5 times the filter it is meant to save and twice the CNN. With native routing (33.5% filtered), total preprocessing was 1.054 ms/record for the adaptive pipeline versus 0.270 for always-P2, and end-to-end latency was 1.52 versus 0.73 ms/record (+107%). In streaming mode (single records), the ensemble’s per-call overhead dominated (32.4 ms/record), giving 35.4 versus 2.1 ms/record. The lightweight sensor (0.30 ms/record batched) reduced this gap but did not close it (end-to-end 0.86 vs. 0.73 ms/record batched; 2.14 vs. 2.12 ms streaming). The largest saving the adaptive routing can produce on native records is (1 − 0.335) × (0.270 − 0.012) = 0.172 ms/record batched and 0.365 ms/record streaming. Both sensors exceed this budget in both modes. The adaptive pipeline is therefore not cheaper than always filtering, and the cost saving does not hold once sensing is included.

### 3.13. Morphology-Sensitive Discrimination

Sub-class AUROCs (Supplementary Table S6) show no localized deficit of filtering that is hidden by macro-AUROC. On native records, the fixed filter P2 was at least as good as unfiltered processing for every ischemic ST-T sub-class (anterior, inferior and lateral ischemia, non-specific ST changes, ST-T changes; differences +0.0016 to +0.0032) and equal for anterior MI. The adaptive policy was 0.0011–0.0028 below the fixed filter for these sub-classes, because it leaves two-thirds of the records unfiltered. Under baseline wander at severity 1.5, unfiltered processing lost most for inferior MI (−0.0067 vs. adaptive), AV block (−0.0093), and right-ventricular hypertrophy (−0.032, n = 12). Lateral MI (n = 20) was the only sub-class for which unfiltered processing was better under baseline wander (+0.008), an estimate based on 20 positive records. Sensitivity at 90% specificity followed the same pattern (Supplementary Table S6). Combined with the fidelity analysis (Section 3.3), these results indicate that the 0.5–40 Hz filter does not degrade ST-segment or Q-wave information at the resolution available at 100 Hz, which is why selective filtering cannot gain by protecting clean records.

### 3.14. Testing the Predictions of the Homeostatic Analogy

Table 10 confronts the four predictions of Table 1 with the test data (pre-registered routing with refitted temperatures; pre-registered sensor unless stated otherwise). With the pre-registered sensor, the share of records routed to the corrective effector rose from 33.5% (native) under every disturbance, but not in a graded way: under synthetic baseline wander it was non-monotone (74.5%, 73.5% and 80.8% at severities 0.5, 1.0 and 1.5); under synthetic EMG and recorded muscle artifact it jumped to 99–100% already at the mildest level (step response, i.e., saturation); a graded rise was seen only for recorded baseline wander (69.9%, 88.9%, 100% at 12, 6, 0 dB) and electrode motion (77.4%, 87.6%, 99.8%). With disturbance-spanning tier edges (Section 3.8), recruitment became graded for baseline wander (60%, 96%, 100%) and recorded muscle artifact (38%, 98%, 100%) but was non-monotone at the mildest EMG level (5.4% native vs. 4.3% at 20 dB). H1 is therefore not supported for the pre-registered sensor and only partly supported after recalibration.

On native test records, the effect of the effector (P2 minus P0) increased from the high- to the low-quality tier for macro-AUROC (0.0000, +0.0016, +0.0021) and macro-F1 (−0.0028, −0.0018, +0.0037). The ordering held in every corrupted bench in which at least two tiers contained ≥ 50 records. H2 is supported, but the magnitudes show why routing cannot pay off: filtering does not harm the high-quality tier in AUROC.

Across each severity gradient, the range of macro-F1 and ECE was smaller for the adaptive policy than for unregulated identity processing in 5/5 noise families for macro-F1 and 4/5 for ECE (e.g., synthetic baseline wander: macro-F1 range 0.0027 vs. 0.0123; recorded baseline wander 0.0016 vs. 0.0218). However, a constant effector (always P2) was equally or more stable (synthetic baseline wander: 0.0005). H3 is supported against no regulation but provides no evidence for regulation over a permanently active effector.

The adaptive policy’s operator cost on native records was one-third of always-on P2 (normalized 0.125 vs. 0.374) at a macro-AUROC within the pre-specified 0.005 non-inferiority margin (0.9223 vs. 0.9231). H4 is supported for operator cost but refuted once the sensor is included (Section 3.12).

## 4. Discussion

### 4.1. Principal Findings

This study asked whether a homeostasis-inspired, quality-gated preprocessing policy can preserve discrimination while improving worst-tier performance and calibration on low-quality ECGs. The answer is negative on the comparison that matters most: the adaptive policy did not outperform the best fixed band-pass filter in discrimination, worst-tier performance, or calibration, on native data or on any of 15 synthetic and real-noise benches, including calibration. What the policy did achieve—clear gains over unfiltered processing under baseline wander and EMG—is equally achieved by always filtering. Its operator-level saving (57% on native records) disappears once the quality sensor is costed (Section 3.12). Stability analyses showed that the qualitative rule “filter only the disturbed tier” is robust, whereas the specific operator is not; all candidate policies lie on a front that spans only 0.012 AUROC. Therefore, this study is presented as a rigorous negative result and uses the post hoc analyses to explain it.

### 4.2. Why the Adaptive Policy Ties the Fixed Filter

Adaptive routing can only beat the best single operator if some operator helps one tier and hurts another. In the pre-registered bank it does not: the per-tier effect of P2 on macro-AUROC was 0.0000 (high), +0.0016 (mid) and +0.0021 (low) on native test records (Section 3.14), and its morphological footprint on clean records was small (ST change ≥ 50 µV in 1.9% of lead-records). With no cost to filtering clean records, “always filter” is a dominant strategy, and the best a controller can do is match it while saving operator time. When a morphology-damaging operator was added (P7), it did not create a trade-off. It harmed clean records as intended but did not repay this on disturbed ones, because the 40 Hz cut-off of P2 already removes most of the EMG-like and recorded muscle noise, and no knee selected it (Section 3.9). Fine-tuning the classifier under the policy did not change the picture either (Section 3.11). This is the first design principle (Section 4.6).

### 4.3. The Role of the Homeostatic Set-Point Term

In the primary run, removing J_2_ did not change the selected operators. In the reproducibility run, it did—the knee with J_2_ was P0/P0/P0 and without J_2_ P0/P0/P3—but this difference reflects the instability of the knee (Section 3.10) rather than a systematic effect of J_2_, and the J_2_-free policy was, if anything, slightly better on test (Table 6). In the pre-registered configuration, therefore, one of the four homeostatic components had no demonstrable function. The reason is structural: along the validation front, J_1_ and J_2_ were almost unrelated (Spearman ρ = −0.10), i.e., the policies that are best on average are also best for the worst tier, so a minimax term adds no information. In the disturbance-rich validation regime with the expanded bank, the conflict was stronger (ρ = −0.30) and removing J_2_ changed the selected operators (P0/P2/P2 with J_2_, P1/P2/P3 without), but the J_2_ knee was not better than always-P2 on any test bench (Section 3.9). J_2_ is therefore not a defining feature of the method but a safeguard that becomes active only when the average and the worst tier compete for the same effector—which the PTB-XL native distribution and a classical filter bank do not provide.

### 4.4. Sensor Saturation, Sensor Cost and the Biological Analogy

Under every EMG bench, and under most real-noise benches, the pre-registered sensor routed (almost) all records to the low-quality tier. The cause is not the classifier inside the sensor but the tier edges: they are tertiles of the native score distribution, so any disturbance outside the native range falls into the top tertile. Retraining the sensor on corrupted copies alone did not cure this (EMG 20 dB: 80% routed; baseline wander ≥ 1.0: 100%); defining the edges on a validation mixture that spans the operating disturbance range did: with these edges the re-calibrated sensor routed 4% of records at EMG 20 dB, 60%, 96% and 100% at baseline-wander severities 0.5, 1.0 and 1.5, and 38%, 98% and 100% for recorded muscle artifact at 12, 6 and 0 dB. A graded sensor did not, however, change test performance (Supplementary Table S5), confirming that in this bank routing precision has little to act on.

The pre-registered cost objective J_4_ covers the operators but not the sensor. End-to-end measurements (Table 9) show that extracting the 88 sensor features costs 0.83 ms/record and the 500-tree ensemble 0.13 ms/record in batched mode (32 ms in streaming mode), against 0.27 ms for the P2 filter itself. The adaptive pipeline is therefore more expensive than always filtering, with either sensor; even the lightweight sensor (0.30 ms/record) exceeds the maximum possible operator saving of 0.17 ms/record. The operator-level saving therefore does not translate into a pipeline-level saving; with the pre-registered sensor, preprocessing even exceeds CNN inference (0.46 ms/record). Energy was not measured.

### 4.5. Does the Biological Framing Earn Its Place?

A biomimetic framing should either generate testable predictions or be presented as a heuristic. In this study, it served both purposes (Table 10). Of the four predictions derived from homeostatic regulation, H2 (local benefit) was supported for macro-F1 and directionally for AUROC. H3 (regulated-variable stability) was supported relative to unregulated processing, but not relative to a constant effector, which was equally or more stable. H4 (economy) was supported only when sensor cost was excluded and was not supported when that cost was included. H1 (graded recruitment) was not supported for the pre-registered sensor, which showed non-monotonic behavior under synthetic baseline wander and step-like saturation under EMG; H1 held only after post hoc redefinition of the tier edges. Thus, in this study, homeostasis functioned as a design heuristic that organized the pipeline into explicit, measurable components and generated testable predictions, rather than as a mechanism that conferred an advantage. The value of this framing lies in the falsifiability of its predictions: their failures point to specific engineering causes.

### 4.6. Falsifiable Design Principles

The negative findings translate into the following principles for bio-inspired adaptive preprocessing. Each is stated with the observation that would refute it.

1. Trade-off precondition. A quality-gated policy can outperform the best fixed operator only if the bank contains an operator whose effect on the target metric changes sign between quality tiers. Refuted by: an adaptive policy that significantly beats the best fixed operator using a bank in which every operator’s per-tier effect has the same sign. Evidence here: P2’s per-tier effect was ≥0 in every tier and the policy tied; adding an operator that damages clean records without helping disturbed ones (P7) did not create a sign change, and no policy selected it.
2. Set-point binding. A worst-group (minimax) objective changes the selected policy only when it conflicts with the average objective along the Pareto front (negative or weak J_1_–J_2_ association). Refuted by: a systematic change of the selected operators by J_2_ when J_1_ and J_2_ are strongly concordant on the front. Evidence: ρ = −0.10 (pre-registered bank, native) vs. −0.30 (expanded bank, disturbed regime); operators changed with J_2_ only in the latter, without a test benefit.
3. Sensor range matching. Quality-tier boundaries must be set on a distribution that spans the disturbances expected in deployment; boundaries fitted on clean data saturate. Refuted by: graded (monotone, non-saturating) routing across an out-of-range disturbance with native-quantile edges. Evidence: EMG 20 dB routing 100% (native edges) vs. 4% (disturbance-spanning edges).
4. Sensor budget inequality. An adaptive pipeline saves computation only if c_sensor_ < (1 − π_low_) (c_effector_ − c_identity_). Refuted by: a net end-to-end saving when the inequality is violated. Evidence: sensor 0.96 ms (pre-registered) and 0.30 ms (lightweight) versus a maximal saving of 0.17 ms/record (batched)—violated by both sensors; no saving.
5. Selection robustness. When objectives differ in range by an order of magnitude more than their sampling noise, min–max-normalized knee selection is unstable; operating points should be selected with explicit non-inferiority tolerances and their stability reported. Refuted by: a stable knee (same operators in ≥90% of bootstrap resamples) under these conditions. Evidence: the modal operator triple was chosen in only 52.5% of bootstrap resamples; the knee differed between the primary and the reproducibility run.
6. Search adequacy. In a small, fully enumerable discrete policy space, an evolutionary optimizer offers no advantage over random search in the discrete choices; differences arise from the selection rule and from fine-tuning continuous parameters. Refuted by: random search failing to visit the optimal discrete configuration at equal budget. Evidence: random search visited all 216 operator triples (Section 3.10).

### 4.7. Limitations

First, the study was conducted using a single dataset. A second 12-lead diagnostic dataset (e.g., CPSC2018 or Chapman–Shaoxing) was not included because the label spaces differ from the PTB-XL super-classes and signal quality would need to be annotated anew. Instead, recorded noise from the NSTDB [24] was used, addressing disturbance realism but not population or device shift. Second, NSTDB noise is single-subject and two-channel, and was resampled and mixed lead-wise at fixed SNRs; therefore, it does not reproduce the inter-lead correlations of real-world artifacts. Third, latency was measured on a single 2-core CPU, and energy consumption was not measured on embedded hardware. Fourth, nested cross-validation across folds 1 to 9 was not performed because it would require retraining the classifier within each outer fold. The selection step was instead assessed using bootstrap and patient-disjoint cross-fitting. These methods quantify selection variability, but not training variability. Fifth, the reproducibility run differs slightly from the primary run because CPU training is not bit-reproducible; the conclusions are unchanged, but the knee is not (Section 3.4). Sixth, the quality labels are PTB-XL’s incomplete artifact annotations. Seventh, all analyses beyond the protocol are post hoc; they are labeled as such and were not used to modify the pre-registered comparison. Eighth, the compact frozen backbone (test macro-AUROC 0.922) is below larger supervised and self-supervised PTB-XL models [3,42,43]; whether a stronger backbone, which may be less sensitive to noise, would leave even less room for adaptive preprocessing was not tested.

### 4.8. Future Work

The principles in Section 4.6 suggest where adaptive preprocessing could pay off: banks containing strong, morphology-altering denoisers (learned diffusion denoisers [26]) whose benefit on noisy records is paid for on clean ones; disturbance families that overlap the diagnostic band (electrode motion), where the present bank failed for every policy; wearable or single-lead recordings with far higher noise prevalence than PTB-XL; continuous rather than tiered gating; and external, cross-device validation with expert quality labels.

## 5. Conclusions

The sensor–controller–effector–set-point structure of physiological homeostasis was translated into an explicit, testable design for ECG preprocessing and calibration. The design was optimized using an established multi-objective evolutionary algorithm under a pre-registered, leakage-controlled protocol, and evaluated on PTB-XL using patient-clustered, multiplicity-adjusted uncertainty estimates, synthetic and recorded noise, and selection-step stability analyses. The adaptive policy did not outperform the best fixed band-pass filter on any endpoint or noise bench. Its advantages over unfiltered processing were also achieved by always applying a filter, while its operator-level savings were offset by the cost of sensing. These negative results have identifiable causes: an operator bank without a tier-dependent trade-off, a worst-tier objective that was rarely binding in the native data, sensor boundaries fitted on clean data, a sensor whose cost exceeded its savings, and an unstable knee-selection rule. These findings translate into six falsifiable design principles. Thus, the homeostatic analogy was useful less as a source of performance gains than as a source of predictions that could fail. Bio-inspired signal-processing designs should be evaluated in the same way.

## Figures and Tables

**Table 1:** Operational mapping between the homeostatic loop and the pipeline, with the testable prediction implied by each element.

Biological Element	Computational Component	Measurable Quantity	Implied, Testable Prediction
Disturbance	Baseline wander, EMG, electrode motion	Corruption type and severity	—
Sensor (receptor)	Quality estimator (88 features)	Quality score; tier q	H1: recruitment of the corrective effector grows monotonically with disturbance severity
Controller	Tier-gated policy θ	Operator index *p_q_*, temperature *T_q_*	H2: the effector’s benefit is local—largest in the low-quality tier, ≤0 in the high-quality tier
Effector	Operator bank P0–P5; logit scaling *z*/*T_q_*	Preprocessed waveform; calibrated probability	—
Regulated (essential) variables	Discrimination, worst-tier performance, calibration, cost	Macro-AUROC, min_q_ macro-F1_q_, class-wise ECE, normalized latency	H3: the regulated variables vary less across disturbance severities than without regulation
Set point	Worst-tier objective J_2_ (Equation (3))	Penalty when the weakest tier falls behind	H4: regulation is economical—lower cost than always-on correction at non-inferior discrimination
Evolutionary shaping of set points	NSGA-II on validation fold; knee; locking	Pareto set; locked θ*	—

**Table 2:** Baseline classifier per seed (validation fold 9).

Seed	Best Epoch	Macro-AUROC	NORM	MI	STTC	CD	HYP
20260901	9	0.9178	0.9377	0.9021	0.9262	0.9161	0.9070
20260902	8	0.9190	0.9388	0.9068	0.9298	0.9230	0.8963
20260903	12	0.9175	0.9396	0.9062	0.9236	0.9184	0.8997
Mean ± SD	—	0.9181 ± 0.0008					

**Table 3:** Composition of the quality tiers (final sensor) and association with tier membership (Cramér’s V; test fold).

Fold	Tier	n	Artifact %	NORM %	MI %	STTC %	CD %	HYP %	Age (Median)
9	High	663	6.6	50.7	23.1	21.9	16.1	13.4	58
9	Mid	725	21.2	44.0	24.1	25.2	26.8	12.7	62
9	Low	758	43.5	39.6	28.0	26.4	25.6	11.5	66.5
10	High	699	7.3	49.8	22.6	23.6	19.7	12.6	61
10	Mid	737	19.0	42.1	26.1	25.4	25.8	12.2	63
10	Low	722	44.6	42.2	27.7	23.4	23.3	11.6	65
10	V, final sensor		0.36	0.07	0.05	0.02	0.06	0.01	
10	V, abandoned SQI		0.02	0.20	0.15	0.08	0.17	0.09	

Artifact: Super-class percentages do not sum to 100 because records may carry several labels.

**Table 4:** Operator bank: validation macro-AUROC when applied to all records (seed-averaged), CPU cost, and waveform fidelity on native test records (*n* = 2158; 12 leads each).

Code	Operator	Val. Macro-AUROC	Cost (Ms/Record)	ST	Δ	Median/p95 (µV)	ST Δ ≥ 50 µV (%)
P0	Identity	0.9263	0.045	0/0	0	0	0
P1	Robust linear detrend	0.9265	0.465	—	—	—	—
P2	Butterworth 0.5–40 Hz	0.9274	0.309	5.2/30.5	1.9	3.2	3.4
P3	Butterworth 0.5–35 Hz	0.9273	0.305	5.2/30.4	2.0	5.0	5.5
P4	Butterworth 1–40 Hz	0.9245	0.293	14.7/88.4	14.3	6.9	4.7
P5	Savitzky–Golay detrend + smoothing	0.9077	0.750	—	—	—	—
P6	P2 + wavelet denoising (post hoc)	0.9275	1.884	6.0/31.4	2.0	4.8	6.2
P7	Butterworth 0.5–20 Hz (post hoc)	0.9208	0.276	6.3/31.8	2.1	34.6	27.6
P8	Two-stage median baseline (post hoc)	0.9264	9.840	12.3/70.0	9.5	3.4	3.1

**Table 5:** Locked test fold (fold 10, n = 2158), native signals.

Policy	Operators (High/Mid/Low)	Macro-AUROC	Macro-F1	ECE	Brier	Worst-Tier F1	Op. Cost (ms/rec.)
B0 Identity, T = 1	P0/P0/P0	0.9219	0.7374	0.0862	0.1047	0.7190	0.045
B1 Identity, calibrated	P0/P0/P0	0.9219	0.7398	0.0846	0.1063	0.7217	0.045
B2 Best fixed filter	P2/P2/P2	**0.9231**	0.7419	**0.0837**	**0.1040**	0.7298	0.309
B3 Random-search knee	P0/P0/P0	0.9215	0.7385	0.0859	0.1050	0.7191	0.045
A1 NSGA-II without J_2_	P0/P0/P3	0.9224	**0.7424**	0.0841	0.1050	**0.7317**	0.132
A2 Pre-registered routing, T = 1	P0/P0/P2	0.9225	0.7411	0.0852	0.1042	0.7299	0.133
**P-locked (pre-registered)**	P0/P0/P2	0.9225	0.7404	0.0839	0.1058	0.7254	0.133
P-refit	P0/P0/P2	0.9223	0.7417	0.0847	0.1057	0.7298	0.133
P-rerun	P0/P0/P0	0.9219	0.7380	0.0869	0.1045	0.7197	0.045

Bold: best value per column. Operator cost excludes the quality sensor.

**Table 6:** Pre-registered comparisons: P-locked minus comparator, patient-clustered bootstrap (1000 resamples), mean [95% CI]; Holm adjustment over the 36 tests of this family. p from the normal approximation to the bootstrap distribution.

Scenario	Comparator	ΔMacro-AUROC	p_Holm	ΔMacro-F1	p_Holm	ΔECE	p_Holm
Native	B1 Identity, cal.	0.0005 [−0.0005, 0.0015]	1.00	0.0008 [−0.0024, 0.0042]	1.00	−0.0009 [−0.0026, 0.0010]	1.00
Native	B2 Fixed P2	−0.0007 [−0.0015, 0.0002]	1.00	−0.0015 [−0.0060, 0.0038]	1.00	0.0002 [−0.0022, 0.0025]	1.00
Native	B3 Random search	0.0009 [−0.0002, 0.0020]	1.00	0.0020 [−0.0023, 0.0067]	1.00	−0.0019 [−0.0047, 0.0011]	1.00
Native	A1 No J_2_	0.0000 [−0.0003, 0.0004]	1.00	−0.0020 [−0.0044, 0.0006]	1.00	0.0001 [−0.0016, 0.0019]	1.00
BW 1.5	B1 Identity, cal.	**0.0047 [0.0028, 0.0065]**	<0.001	0.0104 [0.0033, 0.0173]	0.10	−0.0054 [−0.0091, −0.0018]	0.10
BW 1.5	B2 Fixed P2	−0.0007 [−0.0012, −0.0001]	0.44	−0.0036 [−0.0075, 0.0001]	1.00	0.0025 [−0.0002, 0.0050]	1.00
BW 1.5	B3 Random search	**0.0047 [0.0028, 0.0065]**	<0.001	0.0089 [0.0019, 0.0157]	0.27	**−0.0095 [−0.0135, −0.0052]**	<0.001
BW 1.5	A1 No J_2_	−0.0002 [−0.0005, 0.0001]	1.00	−0.0029 [−0.0056, 0.0000]	0.88	0.0010 [−0.0015, 0.0033]	1.00
EMG 5 dB	B1 Identity, cal.	**0.0023 [0.0013, 0.0032]**	<0.001	0.0071 [0.0021, 0.0124]	0.17	**−0.0064 [−0.0091, −0.0037]**	<0.001
EMG 5 dB	B2 Fixed P2	0.0000 [0.0000, 0.0000]	1.00	−0.0003 [−0.0031, 0.0025]	1.00	−0.0010 [−0.0033, 0.0015]	1.00
EMG 5 dB	B3 Random search	**0.0023 [0.0013, 0.0032]**	<0.001	0.0063 [0.0005, 0.0122]	0.73	**−0.0097 [−0.0129, −0.0064]**	<0.001
EMG 5 dB	A1 No J_2_	*−0.0007 [−0.0011, −0.0004]*	0.006	−0.0053 [−0.0095, −0.0008]	0.39	***0.0059 [0.0035, 0.0083]***	<0.001

Bold: significant after Holm adjustment in favor of P-locked; italics: significant in favor of the comparator. Under EMG, every record is routed to P2, so P-locked and B2 differ only in temperature (ΔAUROC = 0 by construction).

**Table 7:** Stability of the selected policy (validation fold 9).

Analysis	Result
Knee of the pooled front (10 runs)	P0/P0/P0
Knees of the 10 individual runs	P0/P0/P2 × 6; P0/P0/P0 × 3; P0/P0/P3 × 1
Patient bootstrap of fold 9 (B = 200)	P0/P0/P2 52.5%; P0/P0/P3 36.0%; P0/P0/P0 4.5%; P0/P3/P0 4.5%; other 2.5%
— low tier filtered, high/mid unfiltered	88.5%
— temperatures, median [IQR width]	0.872 [0.055], 0.939 [0.035], 1.012 [0.013]
Cross-fitting A to B	P0/P0/P3; test macro-AUROC 0.9224, worst-tier F1 0.7315, ECE 0.0846
Cross-fitting B to A	P0/P0/P2; test macro-AUROC 0.9224, worst-tier F1 0.7239, ECE 0.0924
Random search (24,000 samples): triples visited	216 of 216
Hypervolume: NSGA-II pooled/random non-dominated	1.403/1.362

**Table 8:** Frozen versus policy-adapted classifiers (test fold; temperatures and thresholds refitted on fold 9 for every pipeline).

Pipeline	Native AUROC	Native ECE	Native Worst-Tier F1	BW 1.5 AUROC	EMG 5 dB AUROC	NSTDB ma 0 dB AUROC	NSTDB em 0 dB AUROC
Frozen, identity	0.9219	0.0846	0.7179	0.9178	0.9178	0.9123	0.8999
Frozen, fixed P2	0.9231	0.0837	0.7261	0.9232	0.9201	0.9180	0.9011
Frozen, adaptive	0.9223	0.0847	0.7315	0.9229	0.9201	0.9180	0.9011
FT-identity	0.9228	0.0680	0.7190	0.9186	0.9189	0.9139	0.9013
FT-P2	**0.9239**	**0.0670**	0.7330	**0.9239**	0.9208	0.9191	0.9018
FT-policy	0.9229	0.0681	**0.7342**	0.9238	**0.9211**	**0.9193**	**0.9020**

Note: Bold values indicate the best performance for each metric column.

**Table 9:** Latency per record (ms) on one 2-core CPU (Python 3.12, NumPy 2.4.4/SciPy 1.17.1 PyTorch 2.14.1); native routing (33.5% of records to the low-quality tier).

Pipeline	Sensor (Batched)	Operator (Batched)	CNN (Batched)	End-to-End, Batched	End-to-End, Streaming
Identity	—	0.012	0.462	0.47	1.57
Fixed P2	—	0.270	0.462	0.73	2.12
Adaptive, pre-registered sensor (88 features + ExtraTrees)	0.955	0.098	0.462	1.52	35.43
Adaptive, lightweight sensor (36 features + logistic regression)	0.304	0.098	0.462	0.86	2.14

**Table 10:** Predictions derived from the homeostatic analogy and their test.

Prediction	Test Statistic	Pre-Registered Sensor	Re-Calibrated Sensor, Disturbance-Spanning Edges	Verdict
H1 Graded recruitment	Low-tier share non-decreasing and not saturated at the mildest level, per noise family (5 families)	Non-decreasing in 4/5 (fails for synthetic BW); graded in 2/5 (NSTDB bw, em); step-like saturation for EMG and NSTDB ma	Non-decreasing and graded in 4/5; fails for synthetic EMG (5.4% → 4.3% → 100%)	Not supported (pre-registered); partly supported after recalibration
H2 Local benefit	Effector benefit increases high to low tier	AUROC 0.0000/+0.0016/+0.0021; F1 −0.0028/−0.0018/+0.0037	—	Supported (small)
H3 Stability of regulated variables	Range across severity, adaptive vs. identity vs. always-P2	Smaller than identity in 5/5 (F1), 4/5 (ECE); not smaller than always-P2	Same	Supported vs. no regulation only
H4 Economy	Operator cost < always-P2 at ΔAUROC ≥ −0.005	0.125 vs. 0.374; ΔAUROC − 0.0008	0.020 vs. 0.374; ΔAUROC −0.0012	Supported for operator cost; refuted with sensor cost

## Data Availability

The PTB-XL dataset (version 1.0.3) is publicly available from PhysioNet at https://doi.org/10.13026/kfzx-aw45 (accessed on 30 September 2026) under a CC BY 4.0 license, and the MIT-BIH Noise Stress Test Database at https://doi.org/10.13026/C2HS3T (accessed on 30 September 2026); neither is redistributed here. The analysis code, fixed random seeds, the pre-registered protocol, the locked policy file, all result tables behind the reported numbers, and the figure scripts are provided as Code S1.

## Supplementary Materials

The following supporting information can be downloaded at: https://www.mdpi.com/article/10.3390/biomimetics11100744/s1. Supplementary Material S1—Pre-Registered Experimental Protocol. Figure S1. Pareto fronts of the expanded-bank searches (post hoc analysis 2), projected on macro-AUROC and worst-tier macro-F1. Table S1. All policies on all 16 test benches (locked test fold, n = 2158 per bench). Table S2. Per-class AUROC, F1 and ECE (native, BW 1.5, EMG 5 dB). Table S3. Expanded operator bank (post hoc). Table S4. All patient-clustered bootstrap comparisons (1,000 resamples) with Holm adjustment. Table S5. Quality sensors: routing and performance of the pre-registered routing (P0/P0/P2, re-fitted temperatures). Table S6. Morphology-sensitive analyses. Table S7. Fine-tuning logs (post hoc analysis 5). Code S1. test_per_class_all.csv.

## Abbreviations

The following abbreviations are used in this manuscript:

AUROC	Area under the receiver operating characteristic curve
AUC	Area under the curve
BW	Baseline wander (corruption scenario; severity multiplier 0.5, 1.0, 1.5)
CD	Conduction disturbance (PTB-XL diagnostic super-class)
CI	Confidence interval
CNN	Convolutional neural network
CPU	Central processing unit
CPSC2018	China Physiological Signal Challenge 2018 dataset
dB	Decibel
ECE	Expected calibration error (class-wise, 15 equal-width bins)
ECG	Electrocardiogram/electrocardiography
EMG	Electromyographic (muscle) noise
F1	Harmonic mean of precision and recall
GA	Genetic algorithm
HYP	Hypertrophy (PTB-XL diagnostic super-class)
IQR	Interquartile range
MI	Myocardial infarction (PTB-XL diagnostic super-class)
MIT-BIH	Massachusetts Institute of Technology–Beth Israel Hospital (arrhythmia and noise stress test databases)
NORM	Normal ECG (PTB-XL diagnostic super-class)
NSGA-II	Non-dominated Sorting Genetic Algorithm II
P0–P8	Preprocessing operators (Table 4)
P-locked, P-refit, P-rerun	Versions of the proposed policy (Section 2.9)
PTB-XL	Physikalisch–Technische Bundesanstalt extra-large 12-lead ECG dataset
ROC	Receiver operating characteristic
SD	Standard deviation
SNR	Signal-to-noise ratio
SQI	Signal quality index
STTC	ST/T-wave change (PTB-XL diagnostic super-class)
Tq	Calibration temperature of quality tier q
J_1_–J_4_	Optimization objectives (1 − macro-AUROC; 1 − worst-tier macro-F1; ECE; normalized cost)
