# Supplementary material for: Homeostasis-Inspired, Quality-Adaptive Preprocessing for Multi-Label ECG Diagnosis: A Pre-Registered Multi-Objective Evolutionary Study with Negative Findings and Falsifiable Design Principles
Source: Biomimetics (Basel). 2026 Oct 15;11(10):744. doi: 10.3390/biomimetics11100744 (PMC13702186; doi:10.3390/biomimetics11100744)

Supplementary Material S1 — Pre-Registered Experimental Protocol

S1.1 Study title

Homeostasis-inspired, quality-adaptive preprocessing for robust and calibrated multi-label ECG diagnosis on PTB-XL: a multi-objective evolutionary policy study.

S1.2 Research question

On PTB-XL v1.0.3, can a homeostasis-inspired adaptive policy that selects a preprocessing operator and a calibration temperature per signal-quality tier preserve diagnostic discrimination relative to fixed preprocessing of the same classifier while improving worst-tier performance and probability calibration on low-quality signals?

S1.3 Data and task

PhysioNet PTB-XL v1.0.3, 100 Hz waveforms (records100) and official metadata. Primary task: multi-label prediction of the five diagnostic super-classes NORM, MI, STTC, CD and HYP, derived from scp_statements.csv (diagnostic_class). Only records with at least one diagnostic super-class label enter the analysis set. The official patient-wise split is retained: folds 1–8 training, fold 9 validation, fold 10 locked test. Fold 10 is not used for model, threshold, preprocessing-policy, Pareto-point or optimizer selection.

S1.4 Primary model

A compact one-dimensional residual CNN trainable on CPU (12 leads × 1000 samples → 5 sigmoid outputs); class-weighted BCEWithLogitsLoss; AdamW; early stopping on validation macro-AUROC; training-fold-only standardization. The network is trained under mild stochastic corruption augmentation and then frozen; raw, fixed and adaptive policies are compared on the same weights.

S1.5 Signal-quality index

Per record, quality features normalized with training-fold statistics: low-frequency baseline power ratio, high-frequency noise power ratio, first-difference energy, flat-line fraction, outlier fraction and inter-lead consistency. The metadata fields baseline_drift, static_noise, burst_noise and electrodes_problems serve as independent validation variables and are not an optimizer target. A composite SQI is produced with robust scaling learned from the training folds; records are assigned to low/mid/high quality tiers by training-set thresholds.

S1.6 Operator bank

P0 identity; P1 robust linear detrend; P2 zero-phase Butterworth 0.5–40 Hz; P3 zero-phase Butterworth 0.5–35 Hz; P4 zero-phase Butterworth 1–40 Hz; P5 Savitzky–Golay detrend and light smoothing. The CPU latency of each operator is measured on the same record subset and given to the optimizer as a normalized cost. No energy claim is made.

S1.7 Policy space

$\theta = [p_{high}, p_{mid}, p_{low}, T_{high}, T_{mid}, T_{low}]$, $p_q \in \{0,\dots,5\}$, $T_q \in [0.5, 3.0]$. Operator-bank predictions are pre-computed so that the optimizer selects from a cache.

S1.8 Multi-objective optimization

NSGA-II minimizing J1 = 1 − macro-AUROC, J2 = 1 − $\min_q$ macro-$F1_q$, J3 = ECE and J4 = normalized cost. J2 is the homeostatic feedback term. The primary Pareto point is the knee (minimum Euclidean distance to the normalized ideal point) in validation objective space; the rule is fixed before test evaluation. Budget: population 40, 60 generations, 10 optimizer seeds. A random search with the same evaluation budget is run as a control.

S1.9 Endpoints

Primary: test macro-AUROC, worst-tier macro-F1, class-wise calibration error. Secondary: macro-F1, class-wise AUROC/F1, Brier score, exact-match ratio, preprocessing latency, Pareto hypervolume. Class thresholds are set on fold 9 (maximum F1) and applied unchanged to fold 10; temperatures are not re-fitted on test.

S1.10 Robustness scenarios

Deterministic corruption bank on fold 10: baseline wander and EMG-like band-limited noise, three severity/SNR levels each, with fixed corruption seeds.

S1.11 Baselines and ablations

B0 P0 with global temperature; B1 each fixed operator with global temperature; B2 best global operator selected on validation; B3 random-search adaptive policy; A1 NSGA-II without quality tiers; A2 NSGA-II without J2; A3 NSGA-II with temperature fixed; P full homeostatic NSGA-II policy. (In the analysis, A1 is covered by B2, because a single-tier policy reduces to a fixed operator with a global temperature; the article's labels A1/A2 correspond to protocol A2/A3.)

S1.12 Statistics

At least three model seeds; patient-level paired bootstrap with 95% intervals; Holm correction; emphasis on effect sizes and intervals.

S1.13 Stopping and honesty rules

The test fold is accessed only after all selections are complete. Failed or neutral results are not suppressed. Any forced methodological change is appended to this file with a date and reported in the paper. No number that was not produced by an actual experiment is reported as a result.

Protocol deviation record

The planned unsupervised composite SQI separated artifact_any with AUC = 0.516, and pathology prevalence differed markedly between its tiers. Without accessing the test fold, the method was revised: an ExtraTrees model predicting artifact_any from 88 lead-wise technical features was trained on folds 1–7, tier thresholds were fixed as fold-8 tertiles, and fold 9 was used only for construct validity (ROC-AUC 0.760; average precision 0.515; artifact prevalence 6.6%, 21.2% and 43.5% across tiers; AUC 0.781 within NORM and 0.744 within non-NORM records). Fold 10 remained locked.

S1.14 Revision addendum

The following changes were made during peer review. None of them alters the pre-registered comparison, which is reported unchanged as the primary analysis.

1. **Re-execution.** The complete pipeline was re-executed from the raw files with unchanged code and seeds. The quality sensor reproduced exactly. The CNNs differ slightly because CPU training is not bit-

reproducible. The re-executed knee rule selected P0/P0/P0 instead of the locked P0/P0/P2. The locked policy, exactly as fixed on 7 September 2026, is retained as the primary policy (P-locked); the re-selected knee (P-rerun) and a temperature-refitted version (P-refit) are reported alongside it.

2. **Data audit correction.** The statement that 43,605 file checksums had been verified was incorrect; the audit verified the presence of every header/waveform pair (21,799).
3. **Statistics.** Holm correction was applied to the pre-registered family of 36 comparisons and, separately, to the post hoc family. p-values are taken from the normal approximation to the bootstrap distribution.
4. **Post hoc analyses (labeled as such):**
   – tier composition and morphology fidelity;
   – expanded operator bank (P6–P8) and disturbance-rich validation;
   – re-calibrated and lightweight sensors;
   – real NSTDB noise;
   – fine-tuning under the policy;
   – random-search diagnosis and knee stability;
   – end-to-end latency.

   None of these analyses used fold 10 for any selection.
5. **Worst-tier metric.** Tiers with fewer than 50 records are not scored in the worst-tier metric, because worst-tier F1 on a handful of records is noise. This only affects benches in which almost all records fall into one tier.

Table S1. All policies on all 16 test benches (locked test fold, n = 2,158 per bench)

B0–B3, A1, A2, P-locked, P-refit, P-rerun as defined in Section 2.9. Low-tier share: fraction of records routed to the low-quality tier by the pre-registered sensor. Operator cost excludes the sensor.

Scenario	Policy	Operators	Macro-AUROC	Macro-F1	ECE	Brier	Worst-tier F1	Low-tier share	Op. cost (ms)
Native	B0	P0/P0/P0	0.9219	0.7374	0.0862	0.1047	0.7190	0.3346	0.0450
Native	B1	P0/P0/P0	0.9219	0.7398	0.0846	0.1063	0.7217	0.3346	0.0450
Native	B2	P2/P2/P2	0.9231	0.7419	0.0837	0.1040	0.7298	0.3346	0.3086
Native	B3	P0/P0/P0	0.9215	0.7385	0.0859	0.1050	0.7191	0.3346	0.0450
Native	A1	P0/P0/P3	0.9224	0.7424	0.0841	0.1050	0.7317	0.3346	0.1319
Native	A2	P0/P0/P2	0.9225	0.7411	0.0852	0.1042	0.7299	0.3346	0.1332
Native	P-locked	P0/P0/P2	0.9225	0.7404	0.0839	0.1058	0.7254	0.3346	0.1332
Native	P-refit	P0/P0/P2	0.9223	0.7417	0.0847	0.1057	0.7298	0.3346	0.1332
Native	P-rerun	P0/P0/P0	0.9219	0.7380	0.0869	0.1045	0.7197	0.3346	0.0450
BW 0.5	B0	P0/P0/P0	0.9216	0.7368	0.0875	0.1052	0.7189	0.7447	0.0450
BW 0.5	B1	P0/P0/P0	0.9216	0.7367	0.0851	0.1067	0.7180	0.7447	0.0450
BW 0.5	B2	P2/P2/P2	0.9231	0.7416	0.0841	0.1040	0.7228	0.7447	0.3086
BW 0.5	B3	P0/P0/P0	0.9216	0.7385	0.0873	0.1055	0.7207	0.7447	0.0450
BW 0.5	A1	P0/P0/P3	0.9230	0.7418	0.0839	0.1046	0.7254	0.7447	0.2384
BW 0.5	A2	P0/P0/P2	0.9231	0.7412	0.0847	0.1040	0.7257	0.7447	0.2413
BW 0.5	P-locked	P0/P0/P2	0.9229	0.7373	0.0848	0.1058	0.7188	0.7447	0.2413
BW 0.5	P-refit	P0/P0/P2	0.9231	0.7400	0.0851	0.1050	0.7228	0.7447	0.2413
BW 0.5	P-rerun	P0/P0/P0	0.9216	0.7382	0.0884	0.1050	0.7196	0.7447	0.0450
BW 1.0	B0	P0/P0/P0	0.9202	0.7372	0.0906	0.1069	0.7159	0.7349	0.0450
BW 1.0	B1	P0/P0/P0	0.9202	0.7346	0.0877	0.1083	0.7122	0.7349	0.0450
BW 1.0	B2	P2/P2/P2	0.9231	0.7415	0.0837	0.1040	0.7197	0.7349	0.3086
BW 1.0	B3	P0/P0/P0	0.9201	0.7374	0.0897	0.1072	0.7154	0.7349	0.0450
BW 1.0	A1	P0/P0/P3	0.9228	0.7426	0.0842	0.1049	0.7225	0.7349	0.2359

Scenario	Policy	Operators	Macro-AUROC	Macro-F1	ECE	Brier	Worst-tier F1	Low-tier share	Op. cost (ms)
BW 1.0	A2	P0/P0/P2	0.9229	0.7423	0.0850	0.1042	0.7229	0.7349	0.2387
BW 1.0	P-locked	P0/P0/P2	0.9227	0.7399	0.0850	0.1060	0.7187	0.7349	0.2387
BW 1.0	P-refit	P0/P0/P2	0.9228	0.7395	0.0850	0.1053	0.7197	0.7349	0.2387
BW 1.0	P-rerun	P0/P0/P0	0.9202	0.7353	0.0913	0.1067	0.7145	0.7349	0.0450
BW 1.5	B0	P0/P0/P0	0.9178	0.7298	0.0961	0.1097	0.7074	0.8082	0.0450
BW 1.5	B1	P0/P0/P0	0.9178	0.7274	0.0915	0.1110	0.7060	0.8082	0.0450
BW 1.5	B2	P2/P2/P2	0.9232	0.7414	0.0834	0.1040	0.7212	0.8082	0.3086
BW 1.5	B3	P0/P0/P0	0.9178	0.7289	0.0960	0.1100	0.7069	0.8082	0.0450
BW 1.5	A1	P0/P0/P3	0.9227	0.7407	0.0851	0.1048	0.7225	0.8082	0.2549
BW 1.5	A2	P0/P0/P2	0.9228	0.7420	0.0861	0.1042	0.7238	0.8082	0.2580
BW 1.5	P-locked	P0/P0/P2	0.9225	0.7379	0.0863	0.1060	0.7192	0.8082	0.2580
BW 1.5	P-refit	P0/P0/P2	0.9229	0.7390	0.0852	0.1051	0.7212	0.8082	0.2580
BW 1.5	P-rerun	P0/P0/P0	0.9177	0.7299	0.0964	0.1095	0.7085	0.8082	0.0450
EMG 20 dB	B0	P0/P0/P0	0.9217	0.7384	0.0868	0.1050	0.7384	1.0000	0.0450
EMG 20 dB	B1	P0/P0/P0	0.9217	0.7376	0.0849	0.1066	0.7376	1.0000	0.0450
EMG 20 dB	B2	P2/P2/P2	0.9230	0.7416	0.0841	0.1042	0.7416	1.0000	0.3086
EMG 20 dB	B3	P0/P0/P0	0.9217	0.7385	0.0862	0.1052	0.7385	1.0000	0.0450
EMG 20 dB	A1	P0/P0/P3	0.9229	0.7432	0.0835	0.1041	0.7432	1.0000	0.3047
EMG 20 dB	A2	P0/P0/P2	0.9230	0.7432	0.0839	0.1037	0.7432	1.0000	0.3086
EMG 20 dB	P-locked	P0/P0/P2	0.9230	0.7396	0.0857	0.1059	0.7396	1.0000	0.3086
EMG 20 dB	P-refit	P0/P0/P2	0.9230	0.7416	0.0841	0.1042	0.7416	1.0000	0.3086
EMG 20 dB	P-rerun	P0/P0/P0	0.9217	0.7397	0.0871	0.1049	0.7397	1.0000	0.0450
EMG 10 dB	B0	P0/P0/P0	0.9207	0.7356	0.0946	0.1081	0.7356	1.0000	0.0450
EMG 10 dB	B1	P0/P0/P0	0.9207	0.7334	0.0923	0.1097	0.7334	1.0000	0.0450
EMG 10 dB	B2	P2/P2/P2	0.9223	0.7388	0.0892	0.1066	0.7388	1.0000	0.3086
EMG 10 dB	B3	P0/P0/P0	0.9207	0.7353	0.0945	0.1083	0.7353	1.0000	0.0450
EMG 10 dB	A1	P0/P0/P3	0.9225	0.7419	0.0871	0.1058	0.7419	1.0000	0.3047
EMG 10 dB	A2	P0/P0/P2	0.9223	0.7389	0.0901	0.1062	0.7389	1.0000	0.3086
EMG 10 dB	P-locked	P0/P0/P2	0.9223	0.7389	0.0902	0.1083	0.7389	1.0000	0.3086
EMG 10 dB	P-refit	P0/P0/P2	0.9223	0.7388	0.0892	0.1066	0.7388	1.0000	0.3086
EMG 10 dB	P-rerun	P0/P0/P0	0.9207	0.7356	0.0948	0.1080	0.7356	1.0000	0.0450
EMG 5 dB	B0	P0/P0/P0	0.9178	0.7252	0.1156	0.1173	0.7252	1.0000	0.0450
EMG 5 dB	B1	P0/P0/P0	0.9178	0.7232	0.1114	0.1188	0.7232	1.0000	0.0450
EMG 5 dB	B2	P2/P2/P2	0.9201	0.7306	0.1064	0.1142	0.7306	1.0000	0.3086
EMG 5 dB	B3	P0/P0/P0	0.9178	0.7240	0.1148	0.1174	0.7240	1.0000	0.0450
EMG 5 dB	A1	P0/P0/P3	0.9208	0.7356	0.0989	0.1111	0.7356	1.0000	0.3047
EMG 5 dB	A2	P0/P0/P2	0.9201	0.7315	0.1082	0.1137	0.7315	1.0000	0.3086
EMG 5 dB	P-locked	P0/P0/P2	0.9201	0.7303	0.1052	0.1158	0.7303	1.0000	0.3086
EMG 5 dB	P-refit	P0/P0/P2	0.9201	0.7306	0.1064	0.1142	0.7306	1.0000	0.3086
EMG 5 dB	P-rerun	P0/P0/P0	0.9178	0.7259	0.1162	0.1172	0.7259	1.0000	0.0450
NSTDB bw 12 dB	B0	P0/P0/P0	0.9211	0.7360	0.0894	0.1057	0.7076	0.6993	0.0450
NSTDB bw 12 dB	B1	P0/P0/P0	0.9211	0.7351	0.0862	0.1072	0.7072	0.6993	0.0450
NSTDB bw 12 dB	B2	P2/P2/P2	0.9231	0.7418	0.0839	0.1040	0.7154	0.6993	0.3086
NSTDB bw 12 dB	B3	P0/P0/P0	0.9211	0.7357	0.0890	0.1061	0.7080	0.6993	0.0450

Scenario	Policy	Operators	Macro-AUROC	Macro-F1	ECE	Brier	Worst-tier F1	Low-tier share	Op. cost (ms)
NSTDB bw 12 dB	A1	P0/P0/P3	0.9229	0.7414	0.0843	0.1048	0.7173	0.6993	0.2266
NSTDB bw 12 dB	A2	P0/P0/P2	0.9229	0.7414	0.0850	0.1041	0.7164	0.6993	0.2293
NSTDB bw 12 dB	P-locked	P0/P0/P2	0.9227	0.7378	0.0846	0.1059	0.7109	0.6993	0.2293
NSTDB bw 12 dB	P-refit	P0/P0/P2	0.9228	0.7407	0.0850	0.1053	0.7154	0.6993	0.2293
NSTDB bw 12 dB	P-rerun	P0/P0/P0	0.9211	0.7365	0.0906	0.1054	0.7073	0.6993	0.0450
NSTDB bw 6 dB	B0	P0/P0/P0	0.9197	0.7343	0.0935	0.1083	0.7247	0.8888	0.0450
NSTDB bw 6 dB	B1	P0/P0/P0	0.9197	0.7321	0.0896	0.1097	0.7240	0.8888	0.0450
NSTDB bw 6 dB	B2	P2/P2/P2	0.9231	0.7408	0.0841	0.1042	0.7331	0.8888	0.3086
NSTDB bw 6 dB	B3	P0/P0/P0	0.9196	0.7330	0.0927	0.1085	0.7243	0.8888	0.0450
NSTDB bw 6 dB	A1	P0/P0/P3	0.9229	0.7420	0.0844	0.1047	0.7348	0.8888	0.2759
NSTDB bw 6 dB	A2	P0/P0/P2	0.9230	0.7431	0.0852	0.1042	0.7349	0.8888	0.2793
NSTDB bw 6 dB	P-locked	P0/P0/P2	0.9230	0.7377	0.0859	0.1061	0.7299	0.8888	0.2793
NSTDB bw 6 dB	P-refit	P0/P0/P2	0.9229	0.7402	0.0848	0.1050	0.7331	0.8888	0.2793
NSTDB bw 6 dB	P-rerun	P0/P0/P0	0.9197	0.7341	0.0943	0.1081	0.7246	0.8888	0.0450
NSTDB bw 0 dB	B0	P0/P0/P0	0.9145	0.7197	0.1078	0.1162	0.7197	1.0000	0.0450
NSTDB bw 0 dB	B1	P0/P0/P0	0.9145	0.7180	0.1035	0.1174	0.7180	1.0000	0.0450
NSTDB bw 0 dB	B2	P2/P2/P2	0.9227	0.7403	0.0861	0.1053	0.7403	1.0000	0.3086
NSTDB bw 0 dB	B3	P0/P0/P0	0.9145	0.7195	0.1074	0.1163	0.7195	1.0000	0.0450
NSTDB bw 0 dB	A1	P0/P0/P3	0.9226	0.7412	0.0865	0.1052	0.7412	1.0000	0.3047
NSTDB bw 0 dB	A2	P0/P0/P2	0.9227	0.7426	0.0865	0.1048	0.7426	1.0000	0.3086
NSTDB bw 0 dB	P-locked	P0/P0/P2	0.9227	0.7376	0.0868	0.1069	0.7376	1.0000	0.3086
NSTDB bw 0 dB	P-refit	P0/P0/P2	0.9227	0.7403	0.0861	0.1053	0.7403	1.0000	0.3086
NSTDB bw 0 dB	P-rerun	P0/P0/P0	0.9145	0.7193	0.1080	0.1161	0.7193	1.0000	0.0450
NSTDB ma 12 dB	B0	P0/P0/P0	0.9214	0.7393	0.0897	0.1062	0.7385	0.9866	0.0450
NSTDB ma 12 dB	B1	P0/P0/P0	0.9214	0.7397	0.0879	0.1077	0.7391	0.9866	0.0450
NSTDB ma 12 dB	B2	P2/P2/P2	0.9228	0.7425	0.0847	0.1049	0.7416	0.9866	0.3086
NSTDB ma 12 dB	B3	P0/P0/P0	0.9213	0.7387	0.0891	0.1063	0.7381	0.9866	0.0450
NSTDB ma 12 dB	A1	P0/P0/P3	0.9226	0.7434	0.0848	0.1049	0.7428	0.9866	0.3013

Scenario	Policy	Operators	Macro-AUROC	Macro-F1	ECE	Brier	Worst-tier F1	Low-tier share	Op. cost (ms)
NSTDB ma 12 dB	A2	P0/P0/P2	0.9227	0.7428	0.0859	0.1044	0.7421	0.9866	0.3050
NSTDB ma 12 dB	P-locked	P0/P0/P2	0.9227	0.7407	0.0861	0.1065	0.7401	0.9866	0.3050
NSTDB ma 12 dB	P-refit	P0/P0/P2	0.9227	0.7422	0.0848	0.1050	0.7416	0.9866	0.3050
NSTDB ma 12 dB	P-rerun	P0/P0/P0	0.9214	0.7378	0.0901	0.1061	0.7370	0.9866	0.0450
NSTDB ma 6 dB	B0	P0/P0/P0	0.9195	0.7345	0.0992	0.1108	0.7345	1.0000	0.0450
NSTDB ma 6 dB	B1	P0/P0/P0	0.9195	0.7307	0.0956	0.1122	0.7307	1.0000	0.0450
NSTDB ma 6 dB	B2	P2/P2/P2	0.9220	0.7432	0.0920	0.1077	0.7432	1.0000	0.3086
NSTDB ma 6 dB	B3	P0/P0/P0	0.9195	0.7345	0.0987	0.1109	0.7345	1.0000	0.0450
NSTDB ma 6 dB	A1	P0/P0/P3	0.9218	0.7431	0.0909	0.1075	0.7431	1.0000	0.3047
NSTDB ma 6 dB	A2	P0/P0/P2	0.9220	0.7423	0.0925	0.1073	0.7423	1.0000	0.3086
NSTDB ma 6 dB	P-locked	P0/P0/P2	0.9220	0.7369	0.0925	0.1093	0.7369	1.0000	0.3086
NSTDB ma 6 dB	P-refit	P0/P0/P2	0.9220	0.7432	0.0920	0.1077	0.7432	1.0000	0.3086
NSTDB ma 6 dB	P-rerun	P0/P0/P0	0.9195	0.7340	0.0995	0.1107	0.7340	1.0000	0.0450
NSTDB ma 0 dB	B0	P0/P0/P0	0.9123	0.7118	0.1407	0.1286	0.7118	1.0000	0.0450
NSTDB ma 0 dB	B1	P0/P0/P0	0.9123	0.7059	0.1339	0.1298	0.7059	1.0000	0.0450
NSTDB ma 0 dB	B2	P2/P2/P2	0.9180	0.7215	0.1242	0.1215	0.7215	1.0000	0.3086
NSTDB ma 0 dB	B3	P0/P0/P0	0.9123	0.7107	0.1397	0.1287	0.7107	1.0000	0.0450
NSTDB ma 0 dB	A1	P0/P0/P3	0.9181	0.7226	0.1202	0.1201	0.7226	1.0000	0.3047
NSTDB ma 0 dB	A2	P0/P0/P2	0.9180	0.7228	0.1259	0.1211	0.7228	1.0000	0.3086
NSTDB ma 0 dB	P-locked	P0/P0/P2	0.9180	0.7171	0.1209	0.1232	0.7171	1.0000	0.3086
NSTDB ma 0 dB	P-refit	P0/P0/P2	0.9180	0.7215	0.1242	0.1215	0.7215	1.0000	0.3086
NSTDB ma 0 dB	P-rerun	P0/P0/P0	0.9123	0.7117	0.1415	0.1285	0.7117	1.0000	0.0450
NSTDB em 12 dB	B0	P0/P0/P0	0.9211	0.7393	0.0950	0.1077	0.7185	0.7739	0.0450
NSTDB em 12 dB	B1	P0/P0/P0	0.9211	0.7348	0.0925	0.1091	0.7142	0.7739	0.0450
NSTDB em 12 dB	B2	P2/P2/P2	0.9223	0.7411	0.0901	0.1067	0.7204	0.7739	0.3086
NSTDB em 12 dB	B3	P0/P0/P0	0.9211	0.7383	0.0944	0.1080	0.7178	0.7739	0.0450
NSTDB em 12 dB	A1	P0/P0/P3	0.9221	0.7390	0.0908	0.1073	0.7193	0.7739	0.2460
NSTDB em 12 dB	A2	P0/P0/P2	0.9222	0.7410	0.0920	0.1067	0.7212	0.7739	0.2490

Scenario	Policy	Operators	Macro-AUROC	Macro-F1	ECE	Brier	Worst-tier F1	Low-tier share	Op. cost (ms)
NSTDB em 12 dB	P-locked	P0/P0/P2	0.9220	0.7369	0.0902	0.1085	0.7167	0.7739	0.2490
NSTDB em 12 dB	P-refit	P0/P0/P2	0.9222	0.7390	0.0907	0.1077	0.7204	0.7739	0.2490
NSTDB em 12 dB	P-rerun	P0/P0/P0	0.9211	0.7377	0.0949	0.1074	0.7176	0.7739	0.0450
NSTDB em 6 dB	B0	P0/P0/P0	0.9170	0.7255	0.1218	0.1186	0.7150	0.8758	0.0450
NSTDB em 6 dB	B1	P0/P0/P0	0.9170	0.7192	0.1172	0.1199	0.7095	0.8758	0.0450
NSTDB em 6 dB	B2	P2/P2/P2	0.9180	0.7258	0.1171	0.1182	0.7161	0.8758	0.3086
NSTDB em 6 dB	B3	P0/P0/P0	0.9170	0.7234	0.1208	0.1188	0.7138	0.8758	0.0450
NSTDB em 6 dB	A1	P0/P0/P3	0.9180	0.7259	0.1179	0.1184	0.7168	0.8758	0.2725
NSTDB em 6 dB	A2	P0/P0/P2	0.9181	0.7286	0.1197	0.1179	0.7187	0.8758	0.2759
NSTDB em 6 dB	P-locked	P0/P0/P2	0.9179	0.7234	0.1161	0.1197	0.7144	0.8758	0.2759
NSTDB em 6 dB	P-refit	P0/P0/P2	0.9182	0.7258	0.1173	0.1186	0.7161	0.8758	0.2759
NSTDB em 6 dB	P-rerun	P0/P0/P0	0.9169	0.7248	0.1224	0.1184	0.7151	0.8758	0.0450
NSTDB em 0 dB	B0	P0/P0/P0	0.8999	0.6564	0.2193	0.1662	0.6560	0.9977	0.0450
NSTDB em 0 dB	B1	P0/P0/P0	0.8999	0.6511	0.2164	0.1686	0.6507	0.9977	0.0450
NSTDB em 0 dB	B2	P2/P2/P2	0.9011	0.6546	0.2206	0.1706	0.6544	0.9977	0.3086
NSTDB em 0 dB	B3	P0/P0/P0	0.8999	0.6553	0.2190	0.1664	0.6549	0.9977	0.0450
NSTDB em 0 dB	A1	P0/P0/P3	0.9009	0.6542	0.2210	0.1708	0.6538	0.9977	0.3041
NSTDB em 0 dB	A2	P0/P0/P2	0.9011	0.6570	0.2214	0.1696	0.6566	0.9977	0.3080
NSTDB em 0 dB	P-locked	P0/P0/P2	0.9011	0.6510	0.2184	0.1737	0.6507	0.9977	0.3080
NSTDB em 0 dB	P-refit	P0/P0/P2	0.9011	0.6548	0.2206	0.1706	0.6544	0.9977	0.3080
NSTDB em 0 dB	P-rerun	P0/P0/P0	0.8999	0.6565	0.2195	0.1660	0.6561	0.9977	0.0450

Table S2. Per-class AUROC, F1 and ECE (native, BW 1.5, EMG 5 dB)

Per-class results for all 16 benches are in Code S1 (test_per_class_all.csv).

Scenario	Policy	Class	AUROC	F1	ECE
Native	B0	NORM	0.9421	0.8560	0.0444
Native	B0	MI	0.9116	0.7204	0.1131
Native	B0	STTC	0.9330	0.7522	0.0926
Native	B0	CD	0.9190	0.7589	0.0420
Native	B0	HYP	0.9038	0.5996	0.1389
Native	B1	NORM	0.9421	0.8564	0.0497
Native	B1	MI	0.9116	0.7268	0.1085

Scenario	Policy	Class	AUROC	F1	ECE
Native	B1	STTC	0.9330	0.7526	0.0896
Native	B1	CD	0.9190	0.7612	0.0418
Native	B1	HYP	0.9038	0.6017	0.1336
Native	B2	NORM	0.9429	0.8553	0.0457
Native	B2	MI	0.9125	0.7279	0.1339
Native	B2	STTC	0.9353	0.7547	0.0764
Native	B2	CD	0.9197	0.7625	0.0396
Native	B2	HYP	0.9051	0.6093	0.1230
Native	B3	NORM	0.9416	0.8560	0.0439
Native	B3	MI	0.9111	0.7204	0.1128
Native	B3	STTC	0.9327	0.7526	0.0923
Native	B3	CD	0.9188	0.7581	0.0423
Native	B3	HYP	0.9034	0.6053	0.1383
Native	A1	NORM	0.9429	0.8562	0.0471
Native	A1	MI	0.9112	0.7279	0.1194
Native	A1	STTC	0.9342	0.7513	0.0842
Native	A1	CD	0.9196	0.7625	0.0400
Native	A1	HYP	0.9044	0.6140	0.1300
Native	A2	NORM	0.9428	0.8562	0.0452
Native	A2	MI	0.9112	0.7265	0.1215
Native	A2	STTC	0.9343	0.7522	0.0865
Native	A2	CD	0.9195	0.7628	0.0389
Native	A2	HYP	0.9045	0.6079	0.1341
Native	P-locked	NORM	0.9428	0.8562	0.0480
Native	P-locked	MI	0.9112	0.7237	0.1175
Native	P-locked	STTC	0.9344	0.7511	0.0829
Native	P-locked	CD	0.9193	0.7651	0.0410
Native	P-locked	HYP	0.9046	0.6062	0.1302
Native	P-refit	NORM	0.9428	0.8562	0.0478
Native	P-refit	MI	0.9110	0.7277	0.1171
Native	P-refit	STTC	0.9337	0.7511	0.0848
Native	P-refit	CD	0.9194	0.7630	0.0432
Native	P-refit	HYP	0.9043	0.6106	0.1305
Native	P-rerun	NORM	0.9422	0.8560	0.0434
Native	P-rerun	MI	0.9117	0.7212	0.1140
Native	P-rerun	STTC	0.9330	0.7522	0.0933
Native	P-rerun	CD	0.9190	0.7589	0.0437
Native	P-rerun	HYP	0.9037	0.6014	0.1400
BW 1.5	B0	NORM	0.9387	0.8473	0.0394
BW 1.5	B0	MI	0.9079	0.7142	0.1012
BW 1.5	B0	STTC	0.9308	0.7431	0.1257
BW 1.5	B0	CD	0.9154	0.7542	0.0505
BW 1.5	B0	HYP	0.8961	0.5903	0.1637
BW 1.5	B1	NORM	0.9387	0.8481	0.0416
BW 1.5	B1	MI	0.9079	0.7136	0.0957
BW 1.5	B1	STTC	0.9308	0.7431	0.1224
BW 1.5	B1	CD	0.9154	0.7532	0.0409
BW 1.5	B1	HYP	0.8961	0.5790	0.1569
BW 1.5	B2	NORM	0.9430	0.8556	0.0450
BW 1.5	B2	MI	0.9126	0.7310	0.1324
BW 1.5	B2	STTC	0.9352	0.7538	0.0779
BW 1.5	B2	CD	0.9197	0.7586	0.0372

Scenario	Policy	Class	AUROC	F1	ECE
BW 1.5	B2	HYP	0.9053	0.6082	0.1245
BW 1.5	B3	NORM	0.9387	0.8473	0.0419
BW 1.5	B3	MI	0.9078	0.7119	0.1003
BW 1.5	B3	STTC	0.9309	0.7425	0.1253
BW 1.5	B3	CD	0.9153	0.7542	0.0494
BW 1.5	B3	HYP	0.8963	0.5886	0.1629
BW 1.5	A1	NORM	0.9426	0.8526	0.0461
BW 1.5	A1	MI	0.9116	0.7304	0.1295
BW 1.5	A1	STTC	0.9351	0.7533	0.0824
BW 1.5	A1	CD	0.9191	0.7581	0.0397
BW 1.5	A1	HYP	0.9050	0.6092	0.1277
BW 1.5	A2	NORM	0.9428	0.8534	0.0451
BW 1.5	A2	MI	0.9119	0.7328	0.1295
BW 1.5	A2	STTC	0.9352	0.7535	0.0843
BW 1.5	A2	CD	0.9191	0.7568	0.0399
BW 1.5	A2	HYP	0.9049	0.6132	0.1316
BW 1.5	P-locked	NORM	0.9427	0.8545	0.0519
BW 1.5	P-locked	MI	0.9117	0.7219	0.1250
BW 1.5	P-locked	STTC	0.9349	0.7540	0.0834
BW 1.5	P-locked	CD	0.9188	0.7607	0.0433
BW 1.5	P-locked	HYP	0.9045	0.5983	0.1278
BW 1.5	P-refit	NORM	0.9428	0.8546	0.0457
BW 1.5	P-refit	MI	0.9119	0.7287	0.1274
BW 1.5	P-refit	STTC	0.9353	0.7518	0.0829
BW 1.5	P-refit	CD	0.9193	0.7568	0.0394
BW 1.5	P-refit	HYP	0.9054	0.6031	0.1305
BW 1.5	P-rerun	NORM	0.9388	0.8473	0.0376
BW 1.5	P-rerun	MI	0.9079	0.7131	0.1020
BW 1.5	P-rerun	STTC	0.9306	0.7437	0.1261
BW 1.5	P-rerun	CD	0.9154	0.7537	0.0518
BW 1.5	P-rerun	HYP	0.8960	0.5919	0.1645
EMG 5 dB	B0	NORM	0.9366	0.8394	0.0344
EMG 5 dB	B0	MI	0.9102	0.7206	0.1621
EMG 5 dB	B0	STTC	0.9303	0.7273	0.1666
EMG 5 dB	B0	CD	0.9145	0.7424	0.0478
EMG 5 dB	B0	HYP	0.8976	0.5965	0.1670
EMG 5 dB	B1	NORM	0.9366	0.8385	0.0386
EMG 5 dB	B1	MI	0.9102	0.7183	0.1577
EMG 5 dB	B1	STTC	0.9303	0.7230	0.1633
EMG 5 dB	B1	CD	0.9145	0.7430	0.0377
EMG 5 dB	B1	HYP	0.8976	0.5931	0.1598
EMG 5 dB	B2	NORM	0.9384	0.8403	0.0364
EMG 5 dB	B2	MI	0.9111	0.7150	0.1671
EMG 5 dB	B2	STTC	0.9333	0.7397	0.1440
EMG 5 dB	B2	CD	0.9172	0.7527	0.0349
EMG 5 dB	B2	HYP	0.9004	0.6051	0.1499
EMG 5 dB	B3	NORM	0.9366	0.8382	0.0336
EMG 5 dB	B3	MI	0.9102	0.7200	0.1616
EMG 5 dB	B3	STTC	0.9303	0.7261	0.1662
EMG 5 dB	B3	CD	0.9145	0.7424	0.0464
EMG 5 dB	B3	HYP	0.8976	0.5934	0.1662
EMG 5 dB	A1	NORM	0.9393	0.8445	0.0296

Scenario	Policy	Class	AUROC	F1	ECE
EMG 5 dB	A1	MI	0.9113	0.7247	0.1611
EMG 5 dB	A1	STTC	0.9340	0.7477	0.1260
EMG 5 dB	A1	CD	0.9177	0.7558	0.0366
EMG 5 dB	A1	HYP	0.9018	0.6052	0.1409
EMG 5 dB	A2	NORM	0.9384	0.8403	0.0363
EMG 5 dB	A2	MI	0.9111	0.7159	0.1684
EMG 5 dB	A2	STTC	0.9333	0.7417	0.1453
EMG 5 dB	A2	CD	0.9172	0.7527	0.0388
EMG 5 dB	A2	HYP	0.9004	0.6070	0.1524
EMG 5 dB	P-locked	NORM	0.9384	0.8407	0.0426
EMG 5 dB	P-locked	MI	0.9111	0.7166	0.1640
EMG 5 dB	P-locked	STTC	0.9333	0.7382	0.1409
EMG 5 dB	P-locked	CD	0.9172	0.7537	0.0342
EMG 5 dB	P-locked	HYP	0.9004	0.6020	0.1442
EMG 5 dB	P-refit	NORM	0.9384	0.8403	0.0364
EMG 5 dB	P-refit	MI	0.9111	0.7150	0.1671
EMG 5 dB	P-refit	STTC	0.9333	0.7397	0.1440
EMG 5 dB	P-refit	CD	0.9172	0.7527	0.0349
EMG 5 dB	P-refit	HYP	0.9004	0.6051	0.1499
EMG 5 dB	P-rerun	NORM	0.9366	0.8394	0.0338
EMG 5 dB	P-rerun	MI	0.9102	0.7220	0.1625
EMG 5 dB	P-rerun	STTC	0.9303	0.7284	0.1669
EMG 5 dB	P-rerun	CD	0.9145	0.7411	0.0500
EMG 5 dB	P-rerun	HYP	0.8976	0.5986	0.1678

Table S3. Expanded operator bank (post hoc)

(a) Single operators applied to all validation records (seed-averaged logits), native and disturbed regimes.

Regime	Operator	T	Macro-AUROC	Macro-F1	ECE	Worst-tier F1	tier F1 (high/mid/low)
native	0	0.8600	0.9263	0.7452	0.0856	0.7229	0.761/0.753/0.723
native	1	0.9200	0.9265	0.7464	0.0849	0.7268	0.763/0.751/0.727
native	2	0.9500	0.9274	0.7450	0.0850	0.7297	0.758/0.748/0.730
native	3	0.9300	0.9273	0.7440	0.0843	0.7271	0.757/0.749/0.727
native	4	0.8700	0.9245	0.7347	0.0911	0.7167	0.747/0.740/0.717
native	5	0.7900	0.9077	0.6784	0.1419	0.6692	0.669/0.684/0.676
native	6	0.9000	0.9275	0.7440	0.0860	0.7297	0.756/0.747/0.730
native	7	0.7800	0.9208	0.7235	0.1021	0.7171	0.728/0.723/0.717
native	8	0.9500	0.9264	0.7456	0.0772	0.7278	0.756/0.753/0.728
disturbed	0	0.8400	0.9260	0.7428	0.0868	0.7326	0.763/0.764/0.733
disturbed	1	0.9200	0.9262	0.7442	0.0865	0.7345	0.763/0.764/0.735
disturbed	2	0.9500	0.9273	0.7448	0.0856	0.7373	0.758/0.761/0.737
disturbed	3	0.9400	0.9273	0.7431	0.0849	0.7348	0.757/0.762/0.735
disturbed	4	0.9300	0.9245	0.7344	0.0914	0.7266	0.746/0.752/0.727
disturbed	5	0.6700	0.9077	0.6772	0.1420	0.6702	0.670/0.694/0.672
disturbed	6	0.8900	0.9245	0.7367	0.0884	0.7266	0.755/0.759/0.727
disturbed	7	0.7900	0.9208	0.7228	0.1022	0.7161	0.728/0.739/0.716
disturbed	8	0.9500	0.9262	0.7453	0.0773	0.7372	0.756/0.764/0.737

(b) Knees of the four NSGA-II searches (5 seeds × 40 × 60 each).

Regime/objectives	Operators	Temperatures	J1	J2	J3	J4	Front size	Spearman J1–J2 on front
native_J2	P1/P3/P2	0.825/1.014/0.998	0.0724	0.2699	0.0844	0.0265	107	-0.0803
native_noJ2	P0/P2/P3	0.789/0.953/0.951	0.0724	0.2751	0.0851	0.0192	119	0.3008
disturbed_J2	P0/P2/P2	0.949/1.034/0.881	0.0729	0.2633	0.0858	0.0257	100	-0.2960

Regime/objectives	Operators	Temperatures	J1	J2	J3	J4	Front size	Spearman J1–J2 on front
disturbed_noJ2	P1/P2/P3	0.845/0.930/0.931	0.0726	0.2643	0.0851	0.0270	102	0.3888

(c) Test macro-AUROC and ECE of the knees and comparators.

Scenario	identity_calibrated	best_fixed_native(P6)	best_fixed_disturbed(P2)	P-refit (P0/P0/P2)	P-rerun	knee_native_J2	knee_native_noJ2	knee_disturbed_J2	knee_disturbed_noJ2
Native	0.9219	0.9228	0.9231	0.9223	0.9219	0.9228	0.9229	0.9229	0.9231
BW 0.5	0.9216	0.9228	0.9231	0.9231	0.9216	0.9230	0.9230	0.9227	0.9230
BW 1.0	0.9202	0.9229	0.9231	0.9228	0.9202	0.9231	0.9230	0.9227	0.9230
BW 1.5	0.9178	0.9229	0.9232	0.9229	0.9177	0.9231	0.9231	0.9226	0.9231
EMG 20 dB	0.9217	0.9204	0.9230	0.9230	0.9217	0.9230	0.9229	0.9230	0.9229
EMG 10 dB	0.9207	0.9088	0.9223	0.9223	0.9207	0.9223	0.9225	0.9223	0.9225
EMG 5 dB	0.9178	0.8962	0.9201	0.9201	0.9178	0.9201	0.9208	0.9201	0.9208
NSTDB bw 12 dB	0.9211	0.9228	0.9231	0.9228	0.9211	0.9230	0.9230	0.9227	0.9230
NSTDB bw 6 dB	0.9197	0.9227	0.9231	0.9229	0.9197	0.9231	0.9230	0.9231	0.9230
NSTDB bw 0 dB	0.9145	0.9220	0.9227	0.9227	0.9145	0.9227	0.9226	0.9227	0.9226
NSTDB ma 12 dB	0.9214	0.9217	0.9228	0.9227	0.9214	0.9228	0.9226	0.9228	0.9226
NSTDB ma 6 dB	0.9195	0.9194	0.9220	0.9220	0.9195	0.9220	0.9218	0.9220	0.9218
NSTDB ma 0 dB	0.9123	0.9128	0.9180	0.9180	0.9123	0.9180	0.9181	0.9180	0.9181
NSTDB em 12 dB	0.9211	0.9219	0.9223	0.9222	0.9211	0.9222	0.9221	0.9218	0.9221
NSTDB em 6 dB	0.9170	0.9174	0.9180	0.9182	0.9169	0.9179	0.9179	0.9176	0.9179
NSTDB	0.8999	0.8989	0.9011	0.9011	0.8999	0.9011	0.9009	0.9011	0.9009

Scenario	identity_calibrated	best_fixed_native(P6)	best_fixed_disturbed(P2)	P-refit (P0/P0/P2)	P-rerun	knee_native_J2	knee_native_noJ2	knee_disturbed_J2	knee_disturbed_noJ2
em 0 dB									

Scenario	identity_calibrated	best_fixed_native(P6)	best_fixed_disturbed(P2)	P-refit (P0/P0/P2)	P-rerun	knee_native_J2	knee_native_noJ2	knee_disturbed_J2	knee_disturbed_noJ2
Native	0.0846	0.0840	0.0837	0.0847	0.0869	0.0837	0.0837	0.0830	0.0838
BW 0.5	0.0851	0.0840	0.0841	0.0851	0.0884	0.0844	0.0837	0.0831	0.0840
BW 1.0	0.0877	0.0845	0.0837	0.0850	0.0913	0.0843	0.0836	0.0838	0.0835
BW 1.5	0.0915	0.0846	0.0834	0.0852	0.0964	0.0844	0.0834	0.0828	0.0832
EMG 20 dB	0.0849	0.0870	0.0841	0.0841	0.0871	0.0839	0.0834	0.0845	0.0841
EMG 10 dB	0.0923	0.1151	0.0892	0.0892	0.0948	0.0901	0.0872	0.0888	0.0869
EMG 5 dB	0.1114	0.1535	0.1064	0.1064	0.1162	0.1081	0.0988	0.1056	0.0988
NSTDB bw 12 dB	0.0862	0.0841	0.0839	0.0850	0.0906	0.0839	0.0836	0.0837	0.0839
NSTDB bw 6 dB	0.0896	0.0841	0.0841	0.0848	0.0943	0.0843	0.0835	0.0831	0.0839
NSTDB bw 0 dB	0.1035	0.0867	0.0861	0.0861	0.1080	0.0864	0.0865	0.0856	0.0863
NSTDB ma 12 dB	0.0879	0.0859	0.0847	0.0848	0.0901	0.0856	0.0846	0.0858	0.0848
NSTDB ma 6 dB	0.0956	0.0926	0.0920	0.0920	0.0995	0.0925	0.0910	0.0921	0.0909
NSTDB ma 0 dB	0.1339	0.1257	0.1242	0.1242	0.1415	0.1256	0.1203	0.1214	0.1200
NSTDB em 12 dB	0.0925	0.0917	0.0901	0.0907	0.0949	0.0912	0.0898	0.0895	0.0892
NSTDB em 6 dB	0.1172	0.1208	0.1171	0.1173	0.1224	0.1192	0.1173	0.1162	0.1170

Scenario	identity_calibrated	best_fixed_native(P6)	best_fixed_disturbed(P2)	P-refit (P0/P0/P2)	P-rerun	knee_native_J2	knee_native_noJ2	knee_disturbed_J2	knee_disturbed_noJ2
NSTDB em 0 dB	0.2164	0.2285	0.2206	0.2206	0.2195	0.2214	0.2210	0.2194	0.2207

Table S4. All patient-clustered bootstrap comparisons (1,000 resamples) with Holm adjustment

Family “preregistered”: P-locked vs. B1, B2, B3, A1 in native, BW 1.5 and EMG 5 dB (36 tests); family “post hoc”: all other comparisons. p: normal approximation to the bootstrap distribution; p (percentile): two-sided percentile bootstrap p-value.

Family	Scenario	Policy	Comparator	Metric	Mean Δ	CI low	CI high	p (percentile)	p	p (Holm)
preregistered	Native	P-locked	B1	Δmacro_auc	0.0005	-0.0005	0.0015	0.2620	0.2742	1.0000
preregistered	Native	P-locked	B1	Δmacro_f1	0.0008	-0.0024	0.0042	0.6600	0.6379	1.0000
preregistered	Native	P-locked	B1	Δece	-0.0009	-0.0026	0.0010	0.3860	0.3492	1.0000
preregistered	Native	P-locked	B2	Δmacro_auc	-0.0007	-0.0015	0.0002	0.1520	0.1291	1.0000
preregistered	Native	P-locked	B2	Δmacro_f1	-0.0015	-0.0060	0.0038	0.5220	0.5532	1.0000
preregistered	Native	P-locked	B2	Δece	0.0002	-0.0022	0.0025	0.8480	0.8790	1.0000
preregistered	Native	P-locked	B3	Δmacro_auc	0.0009	-0.0002	0.0020	0.1040	0.1004	1.0000
preregistered	Native	P-locked	B3	Δmacro_f1	0.0020	-0.0023	0.0067	0.4020	0.3820	1.0000
preregistered	Native	P-locked	B3	Δece	-0.0019	-0.0047	0.0011	0.2080	0.2052	1.0000
preregistered	Native	P-locked	A1	Δmacro_auc	0.0000	-0.0003	0.0004	0.9980	0.9875	1.0000
preregistered	Native	P-locked	A1	Δmacro_f1	-0.0020	-0.0044	0.0006	0.1400	0.1266	1.0000
preregistered	Native	P-locked	A1	Δece	0.0001	-0.0016	0.0019	0.9380	0.9445	1.0000
post_hoc	Native	P-refit	B1	Δmacro_auc	0.0004	-0.0005	0.0012	0.3620	0.4053	1.0000
post_hoc	Native	P-refit	B1	Δmacro_f1	0.0021	-0.0010	0.0053	0.2120	0.2004	1.0000
post_hoc	Native	P-refit	B1	Δece	-0.0006	-0.0026	0.0015	0.5600	0.5703	1.0000
post_hoc	Native	P-refit	B2	Δmacro_auc	-0.0008	-0.0017	0.0000	0.0540	0.0578	1.0000
post_hoc	Native	P-refit	B2	Δmacro_f1	-0.0002	-0.0045	0.0048	0.9020	0.9336	1.0000
post_hoc	Native	P-refit	B2	Δece	0.0005	-0.0019	0.0026	0.6660	0.6864	1.0000
post_hoc	Native	P-rerun	B2	Δmacro_auc	-0.0012	-0.0022	-0.0000	0.0440	0.0339	1.0000
post_hoc	Native	P-rerun	B2	Δmacro_f1	-0.0041	-0.0092	0.0010	0.1080	0.1119	1.0000
post_hoc	Native	P-	B2	Δece	0.0025	-	0.0053	0.1160	0.1131	1.0000

Family	Scenario	Policy	Comparator	Metric	Mean Δ	CI low	CI high	p (percentile)	p	p (Holm)
		rerun				0.0007				
post_hoc	BW 0.5	P-locked	B1	Δmacro_auc	0.0013	0.0001	0.0024	0.0380	0.0350	1.0000
post_hoc	BW 0.5	P-locked	B1	Δmacro_f1	0.0006	-0.0045	0.0055	0.8100	0.8137	1.0000
post_hoc	BW 0.5	P-locked	B1	Δece	-0.0004	-0.0031	0.0024	0.7720	0.7928	1.0000
post_hoc	BW 0.5	P-locked	B2	Δmacro_auc	-0.0003	-0.0008	0.0003	0.3740	0.3566	1.0000
post_hoc	BW 0.5	P-locked	B2	Δmacro_f1	-0.0043	-0.0080	-0.0005	0.0320	0.0260	1.0000
post_hoc	BW 0.5	P-locked	B2	Δece	0.0008	-0.0016	0.0033	0.5400	0.5461	1.0000
post_hoc	BW 0.5	P-locked	B3	Δmacro_auc	0.0013	0.0000	0.0025	0.0440	0.0403	1.0000
post_hoc	BW 0.5	P-locked	B3	Δmacro_f1	-0.0012	-0.0061	0.0038	0.6340	0.6466	1.0000
post_hoc	BW 0.5	P-locked	B3	Δece	-0.0019	-0.0054	0.0014	0.2680	0.2662	1.0000
post_hoc	BW 0.5	P-locked	A1	Δmacro_auc	-0.0001	-0.0005	0.0002	0.3780	0.3848	1.0000
post_hoc	BW 0.5	P-locked	A1	Δmacro_f1	-0.0045	-0.0073	-0.0018	0.0000	0.0011	0.2640
post_hoc	BW 0.5	P-locked	A1	Δece	0.0009	-0.0013	0.0030	0.4500	0.4306	1.0000
post_hoc	BW 0.5	P-refit	B1	Δmacro_auc	0.0015	0.0004	0.0026	0.0080	0.0065	1.0000
post_hoc	BW 0.5	P-refit	B1	Δmacro_f1	0.0033	-0.0017	0.0081	0.2040	0.1896	1.0000
post_hoc	BW 0.5	P-refit	B1	Δece	-0.0003	-0.0027	0.0025	0.8200	0.8301	1.0000
post_hoc	BW 0.5	P-refit	B2	Δmacro_auc	0.0000	-0.0005	0.0005	0.9300	0.9490	1.0000
post_hoc	BW 0.5	P-refit	B2	Δmacro_f1	-0.0016	-0.0050	0.0019	0.3400	0.3621	1.0000
post_hoc	BW 0.5	P-refit	B2	Δece	0.0008	-0.0006	0.0022	0.2700	0.2480	1.0000
post_hoc	BW 0.5	P-rerun	B2	Δmacro_auc	-0.0015	-0.0026	-0.0004	0.0120	0.0077	1.0000
post_hoc	BW 0.5	P-rerun	B2	Δmacro_f1	-0.0034	-0.0086	0.0019	0.1960	0.2037	1.0000
post_hoc	BW 0.5	P-rerun	B2	Δece	0.0035	0.0002	0.0067	0.0400	0.0316	1.0000
post_hoc	BW 1.0	P-locked	B1	Δmacro_auc	0.0024	0.0010	0.0038	0.0020	0.0007	0.1836
post_hoc	BW 1.0	P-locked	B1	Δmacro_f1	0.0053	-0.0006	0.0110	0.0720	0.0732	1.0000
post_hoc	BW 1.0	P-locked	B1	Δece	-0.0023	-0.0053	0.0006	0.1380	0.1288	1.0000
post_hoc	BW 1.0	P-locked	B2	Δmacro_auc	-0.0005	-0.0011	0.0001	0.1400	0.1305	1.0000
post_hoc	BW 1.0	P-locked	B2	Δmacro_f1	-0.0017	-0.0054	0.0023	0.3700	0.3944	1.0000
post_hoc	BW 1.0	P-locked	B2	Δece	0.0013	-0.0012	0.0038	0.2780	0.3026	1.0000
post_hoc	BW 1.0	P-locked	B3	Δmacro_auc	0.0025	0.0010	0.0040	0.0020	0.0008	0.1978

Family	Scenario	Policy	Comparator	Metric	Mean Δ	CI low	CI high	p (percentile)	p	p (Holm)
post_hoc	BW 1.0	P-locked	B3	Δmacro_f1	0.0025	-0.0033	0.0082	0.4080	0.3884	1.0000
post_hoc	BW 1.0	P-locked	B3	Δece	-0.0043	-0.0079	-0.0007	0.0220	0.0175	1.0000
post_hoc	BW 1.0	P-locked	A1	Δmacro_auc	-0.0001	-0.0004	0.0003	0.6160	0.6537	1.0000
post_hoc	BW 1.0	P-locked	A1	Δmacro_f1	-0.0028	-0.0054	-0.0001	0.0420	0.0393	1.0000
post_hoc	BW 1.0	P-locked	A1	Δece	0.0008	-0.0014	0.0031	0.4660	0.4612	1.0000
post_hoc	BW 1.0	P-refit	B1	Δmacro_auc	0.0026	0.0012	0.0039	0.0000	0.0002	0.0408
post_hoc	BW 1.0	P-refit	B1	Δmacro_f1	0.0050	-0.0010	0.0108	0.1080	0.0998	1.0000
post_hoc	BW 1.0	P-refit	B1	Δece	-0.0025	-0.0050	0.0002	0.0680	0.0615	1.0000
post_hoc	BW 1.0	P-refit	B2	Δmacro_auc	-0.0003	-0.0009	0.0003	0.2940	0.3105	1.0000
post_hoc	BW 1.0	P-refit	B2	Δmacro_f1	-0.0020	-0.0051	0.0012	0.2120	0.2145	1.0000
post_hoc	BW 1.0	P-refit	B2	Δece	0.0011	-0.0003	0.0027	0.1300	0.1434	1.0000
post_hoc	BW 1.0	P-rerun	B2	Δmacro_auc	-0.0030	-0.0043	-0.0015	0.0000	0.0000	0.0083
post_hoc	BW 1.0	P-rerun	B2	Δmacro_f1	-0.0063	-0.0129	-0.0005	0.0360	0.0462	1.0000
post_hoc	BW 1.0	P-rerun	B2	Δece	0.0071	0.0036	0.0102	0.0000	0.0000	0.0060
preregistered	BW 1.5	P-locked	B1	Δmacro_auc	0.0047	0.0028	0.0065	0.0000	0.0000	0.0000
preregistered	BW 1.5	P-locked	B1	Δmacro_f1	0.0104	0.0033	0.0173	0.0040	0.0037	0.0968
preregistered	BW 1.5	P-locked	B1	Δece	-0.0054	-0.0091	-0.0018	0.0020	0.0036	0.0968
preregistered	BW 1.5	P-locked	B2	Δmacro_auc	-0.0007	-0.0012	-0.0001	0.0240	0.0198	0.4362
preregistered	BW 1.5	P-locked	B2	Δmacro_f1	-0.0036	-0.0075	0.0001	0.0600	0.0644	1.0000
preregistered	BW 1.5	P-locked	B2	Δece	0.0025	-0.0002	0.0050	0.0600	0.0543	1.0000
preregistered	BW 1.5	P-locked	B3	Δmacro_auc	0.0047	0.0028	0.0065	0.0000	0.0000	0.0000
preregistered	BW 1.5	P-locked	B3	Δmacro_f1	0.0089	0.0019	0.0157	0.0120	0.0111	0.2665
preregistered	BW 1.5	P-locked	B3	Δece	-0.0095	-0.0135	-0.0052	0.0000	0.0000	0.0003
preregistered	BW 1.5	P-locked	A1	Δmacro_auc	-0.0002	-0.0005	0.0001	0.2720	0.2534	1.0000
preregistered	BW 1.5	P-locked	A1	Δmacro_f1	-0.0029	-0.0056	-0.0000	0.0480	0.0438	0.8755
preregistered	BW 1.5	P-locked	A1	Δece	0.0010	-0.0015	0.0033	0.4140	0.4174	1.0000
post_hoc	BW 1.5	P-refit	B1	Δmacro_auc	0.0051	0.0033	0.0068	0.0000	0.0000	0.0000
post_hoc	BW 1.5	P-refit	B1	Δmacro_f1	0.0116	0.0044	0.0185	0.0020	0.0013	0.3020
post_hoc	BW 1.5	P-refit	B1	Δece	-0.0063	-0.0093	-0.0033	0.0000	0.0000	0.0102
post_hoc	BW 1.5	P-refit	B2	Δmacro_auc	-	-	0.0003	0.3740	0.3695	1.0000

Family	Scenario	Policy	Comparator	Metric	Mean Δ	CI low	CI high	p (percentile)	p	p (Holm)
					0.0003	0.0008				
post_hoc	BW 1.5	P-refit	B2	Δmacro_f1	-0.0024	-0.0057	0.0008	0.1680	0.1422	1.0000
post_hoc	BW 1.5	P-refit	B2	Δece	0.0016	0.0003	0.0030	0.0140	0.0171	1.0000
post_hoc	BW 1.5	P-rerun	B2	Δmacro_auc	-0.0054	-0.0071	-0.0036	0.0000	0.0000	0.0000
post_hoc	BW 1.5	P-rerun	B2	Δmacro_f1	-0.0115	-0.0190	-0.0039	0.0020	0.0027	0.6277
post_hoc	BW 1.5	P-rerun	B2	Δece	0.0126	0.0090	0.0160	0.0000	0.0000	0.0000
post_hoc	EMG 20 dB	P-locked	B1	Δmacro_auc	0.0013	0.0003	0.0023	0.0120	0.0106	1.0000
post_hoc	EMG 20 dB	P-locked	B1	Δmacro_f1	0.0020	-0.0031	0.0071	0.4380	0.4457	1.0000
post_hoc	EMG 20 dB	P-locked	B1	Δece	0.0000	-0.0028	0.0026	0.9960	0.9952	1.0000
post_hoc	EMG 20 dB	P-locked	B2	Δmacro_auc	-0.0000	0.0000	0.0000	1.0000	1.0000	1.0000
post_hoc	EMG 20 dB	P-locked	B2	Δmacro_f1	-0.0021	-0.0053	0.0013	0.2260	0.2153	1.0000
post_hoc	EMG 20 dB	P-locked	B2	Δece	0.0013	-0.0011	0.0040	0.3000	0.3163	1.0000
post_hoc	EMG 20 dB	P-locked	B3	Δmacro_auc	0.0013	0.0003	0.0023	0.0120	0.0106	1.0000
post_hoc	EMG 20 dB	P-locked	B3	Δmacro_f1	0.0012	-0.0039	0.0065	0.6620	0.6518	1.0000
post_hoc	EMG 20 dB	P-locked	B3	Δece	-0.0007	-0.0044	0.0032	0.6900	0.6994	1.0000
post_hoc	EMG 20 dB	P-locked	A1	Δmacro_auc	0.0002	0.0000	0.0003	0.0200	0.0238	1.0000
post_hoc	EMG 20 dB	P-locked	A1	Δmacro_f1	-0.0037	-0.0069	-0.0005	0.0180	0.0212	1.0000
post_hoc	EMG 20 dB	P-locked	A1	Δece	0.0015	-0.0010	0.0040	0.2140	0.2272	1.0000
post_hoc	EMG 20 dB	P-refit	B1	Δmacro_auc	0.0013	0.0003	0.0023	0.0120	0.0106	1.0000
post_hoc	EMG 20 dB	P-refit	B1	Δmacro_f1	0.0041	-0.0015	0.0095	0.1280	0.1441	1.0000
post_hoc	EMG 20 dB	P-refit	B1	Δece	-0.0013	-0.0037	0.0010	0.2940	0.2816	1.0000
post_hoc	EMG 20 dB	P-refit	B2	Δmacro_auc	0.0000	0.0000	0.0000	1.0000	1.0000	1.0000
post_hoc	EMG 20 dB	P-refit	B2	Δmacro_f1	0.0000	0.0000	0.0000	1.0000	1.0000	1.0000
post_hoc	EMG 20 dB	P-refit	B2	Δece	0.0000	0.0000	0.0000	1.0000	1.0000	1.0000
post_hoc	EMG 20 dB	P-rerun	B2	Δmacro_auc	-0.0013	-0.0023	-0.0003	0.0120	0.0106	1.0000
post_hoc	EMG 20 dB	P-rerun	B2	Δmacro_f1	-0.0021	-0.0070	0.0027	0.3800	0.3966	1.0000
post_hoc	EMG 20 dB	P-rerun	B2	Δece	0.0027	-0.0002	0.0055	0.0680	0.0601	1.0000
post_hoc	EMG 10 dB	P-locked	B1	Δmacro_auc	0.0017	0.0007	0.0027	0.0000	0.0011	0.2554
post_hoc	EMG 10 dB	P-locked	B1	Δmacro_f1	0.0057	0.0010	0.0108	0.0220	0.0233	1.0000

Family	Scenario	Policy	Comparator	Metric	Mean Δ	CI low	CI high	p (percentile)	p	p (Holm)
post_hoc	EMG 10 dB	P-locked	B1	Δece	-0.0022	-0.0050	0.0004	0.0980	0.1022	1.0000
post_hoc	EMG 10 dB	P-locked	B2	Δmacro_auc	0.0000	0.0000	0.0000	1.0000	1.0000	1.0000
post_hoc	EMG 10 dB	P-locked	B2	Δmacro_f1	0.0001	-0.0030	0.0033	0.9700	0.9343	1.0000
post_hoc	EMG 10 dB	P-locked	B2	Δece	0.0005	-0.0019	0.0029	0.6820	0.6643	1.0000
post_hoc	EMG 10 dB	P-locked	B3	Δmacro_auc	0.0017	0.0007	0.0027	0.0000	0.0011	0.2554
post_hoc	EMG 10 dB	P-locked	B3	Δmacro_f1	0.0038	-0.0014	0.0096	0.1720	0.1728	1.0000
post_hoc	EMG 10 dB	P-locked	B3	Δece	-0.0043	-0.0080	-0.0005	0.0200	0.0244	1.0000
post_hoc	EMG 10 dB	P-locked	A1	Δmacro_auc	-0.0001	-0.0004	0.0001	0.2140	0.2433	1.0000
post_hoc	EMG 10 dB	P-locked	A1	Δmacro_f1	-0.0029	-0.0066	0.0007	0.1400	0.1165	1.0000
post_hoc	EMG 10 dB	P-locked	A1	Δece	0.0025	-0.0000	0.0050	0.0520	0.0525	1.0000
post_hoc	EMG 10 dB	P-refit	B1	Δmacro_auc	0.0017	0.0007	0.0027	0.0000	0.0011	0.2554
post_hoc	EMG 10 dB	P-refit	B1	Δmacro_f1	0.0055	-0.0001	0.0109	0.0540	0.0471	1.0000
post_hoc	EMG 10 dB	P-refit	B1	Δece	-0.0028	-0.0051	-0.0005	0.0160	0.0169	1.0000
post_hoc	EMG 10 dB	P-refit	B2	Δmacro_auc	0.0000	0.0000	0.0000	1.0000	1.0000	1.0000
post_hoc	EMG 10 dB	P-refit	B2	Δmacro_f1	0.0000	0.0000	0.0000	1.0000	1.0000	1.0000
post_hoc	EMG 10 dB	P-refit	B2	Δece	0.0000	0.0000	0.0000	1.0000	1.0000	1.0000
post_hoc	EMG 10 dB	P-rerun	B2	Δmacro_auc	-0.0017	-0.0027	-0.0007	0.0000	0.0011	0.2554
post_hoc	EMG 10 dB	P-rerun	B2	Δmacro_f1	-0.0033	-0.0083	0.0014	0.1980	0.1770	1.0000
post_hoc	EMG 10 dB	P-rerun	B2	Δece	0.0055	0.0028	0.0083	0.0000	0.0001	0.0219
preregistered	EMG 5 dB	P-locked	B1	Δmacro_auc	0.0023	0.0013	0.0032	0.0000	0.0000	0.0003
preregistered	EMG 5 dB	P-locked	B1	Δmacro_f1	0.0071	0.0021	0.0124	0.0060	0.0069	0.1726
preregistered	EMG 5 dB	P-locked	B1	Δece	-0.0064	-0.0091	-0.0037	0.0000	0.0000	0.0001
preregistered	EMG 5 dB	P-locked	B2	Δmacro_auc	0.0000	0.0000	0.0000	1.0000	1.0000	1.0000
preregistered	EMG 5 dB	P-locked	B2	Δmacro_f1	-0.0003	-0.0031	0.0025	0.8240	0.8232	1.0000
preregistered	EMG 5 dB	P-locked	B2	Δece	-0.0010	-0.0033	0.0015	0.4240	0.4011	1.0000
preregistered	EMG 5 dB	P-locked	B3	Δmacro_auc	0.0023	0.0013	0.0032	0.0000	0.0000	0.0003
preregistered	EMG 5 dB	P-locked	B3	Δmacro_f1	0.0063	0.0005	0.0122	0.0400	0.0348	0.7298
preregistered	EMG 5 dB	P-locked	B3	Δece	-0.0097	-0.0129	-0.0064	0.0000	0.0000	0.0000

Family	Scenario	Policy	Comparator	Metric	Mean Δ	CI low	CI high	p (percentile)	p	p (Holm)
preregistered	EMG 5 dB	P-locked	A1	Δmacro_auc	-0.0007	-0.0011	-0.0004	0.0000	0.0002	0.0057
preregistered	EMG 5 dB	P-locked	A1	Δmacro_f1	-0.0053	-0.0095	-0.0008	0.0140	0.0168	0.3865
preregistered	EMG 5 dB	P-locked	A1	Δece	0.0059	0.0035	0.0083	0.0000	0.0000	0.0000
post_hoc	EMG 5 dB	P-refit	B1	Δmacro_auc	0.0023	0.0013	0.0032	0.0000	0.0000	0.0023
post_hoc	EMG 5 dB	P-refit	B1	Δmacro_f1	0.0075	0.0021	0.0127	0.0060	0.0055	1.0000
post_hoc	EMG 5 dB	P-refit	B1	Δece	-0.0054	-0.0081	-0.0030	0.0000	0.0000	0.0072
post_hoc	EMG 5 dB	P-refit	B2	Δmacro_auc	0.0000	0.0000	0.0000	1.0000	1.0000	1.0000
post_hoc	EMG 5 dB	P-refit	B2	Δmacro_f1	0.0000	0.0000	0.0000	1.0000	1.0000	1.0000
post_hoc	EMG 5 dB	P-refit	B2	Δece	0.0000	0.0000	0.0000	1.0000	1.0000	1.0000
post_hoc	EMG 5 dB	P-rerun	B2	Δmacro_auc	-0.0023	-0.0032	-0.0013	0.0000	0.0000	0.0023
post_hoc	EMG 5 dB	P-rerun	B2	Δmacro_f1	-0.0048	-0.0102	0.0006	0.0840	0.0823	1.0000
post_hoc	EMG 5 dB	P-rerun	B2	Δece	0.0099	0.0073	0.0124	0.0000	0.0000	0.0000
post_hoc	NSTDB bw 12 dB	P-locked	B1	Δmacro_auc	0.0016	0.0005	0.0027	0.0020	0.0049	1.0000
post_hoc	NSTDB bw 12 dB	P-locked	B1	Δmacro_f1	0.0027	-0.0024	0.0079	0.3080	0.3057	1.0000
post_hoc	NSTDB bw 12 dB	P-locked	B1	Δece	-0.0018	-0.0044	0.0007	0.1680	0.1652	1.0000
post_hoc	NSTDB bw 12 dB	P-locked	B2	Δmacro_auc	-0.0004	-0.0009	0.0003	0.2080	0.2098	1.0000
post_hoc	NSTDB bw 12 dB	P-locked	B2	Δmacro_f1	-0.0040	-0.0081	0.0002	0.0640	0.0587	1.0000
post_hoc	NSTDB bw 12 dB	P-locked	B2	Δece	0.0006	-0.0017	0.0030	0.5880	0.5879	1.0000
post_hoc	NSTDB bw 12 dB	P-locked	B3	Δmacro_auc	0.0016	0.0004	0.0028	0.0060	0.0065	1.0000
post_hoc	NSTDB bw 12 dB	P-locked	B3	Δmacro_f1	0.0022	-0.0034	0.0075	0.4160	0.4361	1.0000
post_hoc	NSTDB bw 12 dB	P-locked	B3	Δece	-0.0036	-0.0070	-0.0003	0.0300	0.0360	1.0000
post_hoc	NSTDB bw 12 dB	P-locked	A1	Δmacro_auc	-0.0001	-0.0005	0.0002	0.4060	0.4198	1.0000
post_hoc	NSTDB bw 12 dB	P-locked	A1	Δmacro_f1	-0.0036	-0.0060	-0.0013	0.0080	0.0032	0.7329

Family	Scenario	Policy	Comparator	Metric	Mean Δ	CI low	CI high	p (percentile)	p	p (Holm)
post_hoc	NSTDB bw 12 dB	P-locked	A1	Δece	0.0005	-0.0015	0.0028	0.6540	0.6449	1.0000
post_hoc	NSTDB bw 12 dB	P-refit	B1	Δmacro_auc	0.0017	0.0007	0.0028	0.0020	0.0016	0.3743
post_hoc	NSTDB bw 12 dB	P-refit	B1	Δmacro_f1	0.0056	0.0001	0.0110	0.0360	0.0441	1.0000
post_hoc	NSTDB bw 12 dB	P-refit	B1	Δece	-0.0015	-0.0038	0.0011	0.2700	0.2436	1.0000
post_hoc	NSTDB bw 12 dB	P-refit	B2	Δmacro_auc	-0.0003	-0.0009	0.0004	0.4100	0.4431	1.0000
post_hoc	NSTDB bw 12 dB	P-refit	B2	Δmacro_f1	-0.0011	-0.0047	0.0029	0.5500	0.5543	1.0000
post_hoc	NSTDB bw 12 dB	P-refit	B2	Δece	0.0010	-0.0008	0.0026	0.2180	0.2495	1.0000
post_hoc	NSTDB bw 12 dB	P-rerun	B2	Δmacro_auc	-0.0020	-0.0031	-0.0009	0.0000	0.0004	0.1113
post_hoc	NSTDB bw 12 dB	P-rerun	B2	Δmacro_f1	-0.0053	-0.0111	0.0007	0.0900	0.0790	1.0000
post_hoc	NSTDB bw 12 dB	P-rerun	B2	Δece	0.0059	0.0027	0.0090	0.0000	0.0002	0.0605
post_hoc	NSTDB bw 6 dB	P-locked	B1	Δmacro_auc	0.0033	0.0019	0.0048	0.0000	0.0000	0.0027
post_hoc	NSTDB bw 6 dB	P-locked	B1	Δmacro_f1	0.0056	-0.0007	0.0117	0.0960	0.0793	1.0000
post_hoc	NSTDB bw 6 dB	P-locked	B1	Δece	-0.0042	-0.0075	-0.0010	0.0120	0.0107	1.0000
post_hoc	NSTDB bw 6 dB	P-locked	B2	Δmacro_auc	-0.0001	-0.0006	0.0004	0.7120	0.7178	1.0000
post_hoc	NSTDB bw 6 dB	P-locked	B2	Δmacro_f1	-0.0032	-0.0066	0.0004	0.0820	0.0700	1.0000
post_hoc	NSTDB bw 6 dB	P-locked	B2	Δece	0.0012	-0.0014	0.0038	0.3560	0.3693	1.0000
post_hoc	NSTDB bw 6 dB	P-locked	B3	Δmacro_auc	0.0034	0.0020	0.0049	0.0000	0.0000	0.0022
post_hoc	NSTDB bw 6 dB	P-locked	B3	Δmacro_f1	0.0047	-0.0015	0.0109	0.1360	0.1384	1.0000
post_hoc	NSTDB bw 6 dB	P-locked	B3	Δece	-0.0064	-0.0104	-0.0026	0.0000	0.0011	0.2581
post_hoc	NSTDB bw 6 dB	P-locked	A1	Δmacro_auc	0.0001	-0.0001	0.0004	0.3880	0.3832	1.0000
post_hoc	NSTDB bw 6 dB	P-locked	A1	Δmacro_f1	-0.0044	-0.0071	-0.0017	0.0000	0.0014	0.3319
post_hoc	NSTDB bw 6 dB	P-locked	A1	Δece	0.0010	-0.0018	0.0035	0.4640	0.4795	1.0000
post_hoc	NSTDB bw 6 dB	P-refit	B1	Δmacro_auc	0.0032	0.0018	0.0046	0.0000	0.0000	0.0023
post_hoc	NSTDB	P-refit	B1	Δmacro_f1	0.0081	0.0014	0.0148	0.0220	0.0183	1.0000

Family	Scenario	Policy	Comparator	Metric	Mean Δ	CI low	CI high	p (percentile)	p	p (Holm)
	bw 6 dB									
post_hoc	NSTDB bw 6 dB	P-refit	B1	Δece	-0.0048	-0.0073	-0.0022	0.0000	0.0002	0.0524
post_hoc	NSTDB bw 6 dB	P-refit	B2	Δmacro_auc	-0.0002	-0.0007	0.0003	0.3560	0.3671	1.0000
post_hoc	NSTDB bw 6 dB	P-refit	B2	Δmacro_f1	-0.0007	-0.0033	0.0023	0.6100	0.6434	1.0000
post_hoc	NSTDB bw 6 dB	P-refit	B2	Δece	0.0006	-0.0005	0.0017	0.2780	0.2850	1.0000
post_hoc	NSTDB bw 6 dB	P-rerun	B2	Δmacro_auc	-0.0034	-0.0048	-0.0020	0.0000	0.0000	0.0007
post_hoc	NSTDB bw 6 dB	P-rerun	B2	Δmacro_f1	-0.0068	-0.0136	0.0004	0.0600	0.0571	1.0000
post_hoc	NSTDB bw 6 dB	P-rerun	B2	Δece	0.0092	0.0059	0.0123	0.0000	0.0000	0.0000
post_hoc	NSTDB bw 0 dB	P-locked	B1	Δmacro_auc	0.0083	0.0061	0.0107	0.0000	0.0000	0.0000
post_hoc	NSTDB bw 0 dB	P-locked	B1	Δmacro_f1	0.0195	0.0113	0.0276	0.0000	0.0000	0.0008
post_hoc	NSTDB bw 0 dB	P-locked	B1	Δece	-0.0159	-0.0203	-0.0118	0.0000	0.0000	0.0000
post_hoc	NSTDB bw 0 dB	P-locked	B2	Δmacro_auc	0.0000	0.0000	0.0000	1.0000	1.0000	1.0000
post_hoc	NSTDB bw 0 dB	P-locked	B2	Δmacro_f1	-0.0027	-0.0055	0.0003	0.0800	0.0668	1.0000
post_hoc	NSTDB bw 0 dB	P-locked	B2	Δece	0.0005	-0.0020	0.0032	0.6580	0.6776	1.0000
post_hoc	NSTDB bw 0 dB	P-locked	B3	Δmacro_auc	0.0083	0.0061	0.0107	0.0000	0.0000	0.0000
post_hoc	NSTDB bw 0 dB	P-locked	B3	Δmacro_f1	0.0180	0.0092	0.0264	0.0000	0.0000	0.0108
post_hoc	NSTDB bw 0 dB	P-locked	B3	Δece	-0.0200	-0.0246	-0.0155	0.0000	0.0000	0.0000
post_hoc	NSTDB bw 0 dB	P-locked	A1	Δmacro_auc	0.0001	-0.0000	0.0003	0.0960	0.1007	1.0000
post_hoc	NSTDB bw 0 dB	P-locked	A1	Δmacro_f1	-0.0036	-0.0064	-0.0003	0.0320	0.0188	1.0000
post_hoc	NSTDB bw 0 dB	P-locked	A1	Δece	0.0004	-0.0020	0.0029	0.7340	0.7278	1.0000
post_hoc	NSTDB bw 0 dB	P-refit	B1	Δmacro_auc	0.0083	0.0061	0.0107	0.0000	0.0000	0.0000
post_hoc	NSTDB bw 0 dB	P-refit	B1	Δmacro_f1	0.0222	0.0135	0.0309	0.0000	0.0000	0.0001
post_hoc	NSTDB bw 0 dB	P-refit	B1	Δece	-0.0165	-0.0201	-0.0126	0.0000	0.0000	0.0000
post_hoc	NSTDB bw 0 dB	P-refit	B2	Δmacro_auc	0.0000	0.0000	0.0000	1.0000	1.0000	1.0000
post_hoc	NSTDB bw 0 dB	P-refit	B2	Δmacro_f1	0.0000	0.0000	0.0000	1.0000	1.0000	1.0000
post_hoc	NSTDB bw 0 dB	P-refit	B2	Δece	0.0000	0.0000	0.0000	1.0000	1.0000	1.0000
post_hoc	NSTDB bw 0 dB	P-rerun	B2	Δmacro_auc	-0.0083	-0.0107	-0.0061	0.0000	0.0000	0.0000
post_hoc	NSTDB bw 0 dB	P-rerun	B2	Δmacro_f1	-0.0209	-0.0299	-0.0118	0.0000	0.0000	0.0016
post_hoc	NSTDB	P-	B2	Δece	0.0213	0.0174	0.0252	0.0000	0.0000	0.0000

Family	Scenario	Policy	Comparator	Metric	Mean Δ	CI low	CI high	p (percentile)	p	p (Holm)
	bw 0 dB	rerun								
post_hoc	NSTDB ma 12 dB	P-locked	B1	Δmacro_auc	0.0014	0.0003	0.0024	0.0200	0.0137	1.0000
post_hoc	NSTDB ma 12 dB	P-locked	B1	Δmacro_f1	0.0009	-0.0043	0.0063	0.7520	0.7367	1.0000
post_hoc	NSTDB ma 12 dB	P-locked	B1	Δece	-0.0017	-0.0046	0.0012	0.2800	0.2547	1.0000
post_hoc	NSTDB ma 12 dB	P-locked	B2	Δmacro_auc	-0.0001	-0.0001	-0.0000	0.0420	0.0583	1.0000
post_hoc	NSTDB ma 12 dB	P-locked	B2	Δmacro_f1	-0.0018	-0.0048	0.0012	0.2320	0.2286	1.0000
post_hoc	NSTDB ma 12 dB	P-locked	B2	Δece	0.0016	-0.0010	0.0042	0.2300	0.2298	1.0000
post_hoc	NSTDB ma 12 dB	P-locked	B3	Δmacro_auc	0.0014	0.0003	0.0024	0.0180	0.0134	1.0000
post_hoc	NSTDB ma 12 dB	P-locked	B3	Δmacro_f1	0.0020	-0.0033	0.0074	0.4780	0.4698	1.0000
post_hoc	NSTDB ma 12 dB	P-locked	B3	Δece	-0.0026	-0.0064	0.0011	0.1880	0.1659	1.0000
post_hoc	NSTDB ma 12 dB	P-locked	A1	Δmacro_auc	0.0002	0.0000	0.0003	0.0320	0.0237	1.0000
post_hoc	NSTDB ma 12 dB	P-locked	A1	Δmacro_f1	-0.0027	-0.0057	0.0004	0.0840	0.0847	1.0000
post_hoc	NSTDB ma 12 dB	P-locked	A1	Δece	0.0014	-0.0013	0.0041	0.3160	0.3002	1.0000
post_hoc	NSTDB ma 12 dB	P-refit	B1	Δmacro_auc	0.0013	0.0002	0.0024	0.0200	0.0150	1.0000
post_hoc	NSTDB ma 12 dB	P-refit	B1	Δmacro_f1	0.0024	-0.0034	0.0080	0.4440	0.4059	1.0000
post_hoc	NSTDB ma 12 dB	P-refit	B1	Δece	-0.0032	-0.0055	-0.0008	0.0080	0.0082	1.0000
post_hoc	NSTDB ma 12 dB	P-refit	B2	Δmacro_auc	-0.0001	-0.0002	0.0000	0.1400	0.1581	1.0000
post_hoc	NSTDB ma 12 dB	P-refit	B2	Δmacro_f1	-0.0003	-0.0012	0.0004	0.4280	0.4280	1.0000
post_hoc	NSTDB ma 12 dB	P-refit	B2	Δece	0.0001	-0.0001	0.0002	0.1760	0.2311	1.0000
post_hoc	NSTDB ma 12 dB	P-rerun	B2	Δmacro_auc	-0.0014	-0.0025	-0.0003	0.0160	0.0092	1.0000

Family	Scenario	Policy	Comparator	Metric	Mean Δ	CI low	CI high	p (percentile)	p	p (Holm)
post_hoc	NSTDB ma 12 dB	P-rerun	B2	Δmacro_f1	-0.0047	-0.0102	0.0010	0.0920	0.0967	1.0000
post_hoc	NSTDB ma 12 dB	P-rerun	B2	Δece	0.0050	0.0021	0.0077	0.0000	0.0005	0.1206
post_hoc	NSTDB ma 6 dB	P-locked	B1	Δmacro_auc	0.0024	0.0012	0.0036	0.0000	0.0001	0.0190
post_hoc	NSTDB ma 6 dB	P-locked	B1	Δmacro_f1	0.0062	0.0005	0.0124	0.0340	0.0407	1.0000
post_hoc	NSTDB ma 6 dB	P-locked	B1	Δece	-0.0033	-0.0061	-0.0002	0.0400	0.0266	1.0000
post_hoc	NSTDB ma 6 dB	P-locked	B2	Δmacro_auc	-0.0000	0.0000	0.0000	1.0000	1.0000	1.0000
post_hoc	NSTDB ma 6 dB	P-locked	B2	Δmacro_f1	-0.0062	-0.0089	-0.0034	0.0000	0.0000	0.0031
post_hoc	NSTDB ma 6 dB	P-locked	B2	Δece	0.0006	-0.0018	0.0032	0.6400	0.6599	1.0000
post_hoc	NSTDB ma 6 dB	P-locked	B3	Δmacro_auc	0.0024	0.0012	0.0036	0.0000	0.0001	0.0190
post_hoc	NSTDB ma 6 dB	P-locked	B3	Δmacro_f1	0.0025	-0.0032	0.0086	0.4260	0.4070	1.0000
post_hoc	NSTDB ma 6 dB	P-locked	B3	Δece	-0.0063	-0.0099	-0.0025	0.0000	0.0008	0.1863
post_hoc	NSTDB ma 6 dB	P-locked	A1	Δmacro_auc	0.0002	0.0000	0.0004	0.0340	0.0359	1.0000
post_hoc	NSTDB ma 6 dB	P-locked	A1	Δmacro_f1	-0.0060	-0.0091	-0.0028	0.0000	0.0002	0.0419
post_hoc	NSTDB ma 6 dB	P-locked	A1	Δece	0.0013	-0.0011	0.0039	0.3600	0.3036	1.0000
post_hoc	NSTDB ma 6 dB	P-refit	B1	Δmacro_auc	0.0024	0.0012	0.0036	0.0000	0.0001	0.0190
post_hoc	NSTDB ma 6 dB	P-refit	B1	Δmacro_f1	0.0124	0.0062	0.0187	0.0000	0.0001	0.0251
post_hoc	NSTDB ma 6 dB	P-refit	B1	Δece	-0.0039	-0.0062	-0.0014	0.0020	0.0014	0.3240
post_hoc	NSTDB ma 6 dB	P-refit	B2	Δmacro_auc	0.0000	0.0000	0.0000	1.0000	1.0000	1.0000
post_hoc	NSTDB ma 6 dB	P-refit	B2	Δmacro_f1	0.0000	0.0000	0.0000	1.0000	1.0000	1.0000
post_hoc	NSTDB ma 6 dB	P-refit	B2	Δece	0.0000	0.0000	0.0000	1.0000	1.0000	1.0000
post_hoc	NSTDB ma 6 dB	P-rerun	B2	Δmacro_auc	-0.0024	-0.0036	-0.0012	0.0000	0.0001	0.0190
post_hoc	NSTDB ma 6 dB	P-rerun	B2	Δmacro_f1	-0.0092	-0.0152	-0.0035	0.0000	0.0020	0.4661
post_hoc	NSTDB ma 6 dB	P-rerun	B2	Δece	0.0078	0.0048	0.0106	0.0000	0.0000	0.0000
post_hoc	NSTDB ma 0 dB	P-locked	B1	Δmacro_auc	0.0057	0.0039	0.0077	0.0000	0.0000	0.0000
post_hoc	NSTDB ma 0 dB	P-locked	B1	Δmacro_f1	0.0112	0.0043	0.0183	0.0060	0.0018	0.4144
post_hoc	NSTDB ma 0 dB	P-locked	B1	Δece	-0.0126	-0.0158	-0.0094	0.0000	0.0000	0.0000
post_hoc	NSTDB ma 0 dB	P-locked	B2	Δmacro_auc	0.0000	0.0000	0.0000	1.0000	1.0000	1.0000

Family	Scenario	Policy	Comparator	Metric	Mean Δ	CI low	CI high	p (percentile)	p	p (Holm)
post_hoc	NSTDB ma 0 dB	P-locked	B2	Δmacro_f1	-0.0043	-0.0073	-0.0011	0.0040	0.0069	1.0000
post_hoc	NSTDB ma 0 dB	P-locked	B2	Δece	-0.0029	-0.0051	-0.0005	0.0220	0.0142	1.0000
post_hoc	NSTDB ma 0 dB	P-locked	B3	Δmacro_auc	0.0057	0.0039	0.0077	0.0000	0.0000	0.0000
post_hoc	NSTDB ma 0 dB	P-locked	B3	Δmacro_f1	0.0063	-0.0007	0.0139	0.0800	0.0889	1.0000
post_hoc	NSTDB ma 0 dB	P-locked	B3	Δece	-0.0187	-0.0224	-0.0150	0.0000	0.0000	0.0000
post_hoc	NSTDB ma 0 dB	P-locked	A1	Δmacro_auc	-0.0001	-0.0003	0.0002	0.5480	0.5476	1.0000
post_hoc	NSTDB ma 0 dB	P-locked	A1	Δmacro_f1	-0.0055	-0.0093	-0.0016	0.0040	0.0056	1.0000
post_hoc	NSTDB ma 0 dB	P-locked	A1	Δece	0.0007	-0.0016	0.0031	0.6020	0.5804	1.0000
post_hoc	NSTDB ma 0 dB	P-refit	B1	Δmacro_auc	0.0057	0.0039	0.0077	0.0000	0.0000	0.0000
post_hoc	NSTDB ma 0 dB	P-refit	B1	Δmacro_f1	0.0155	0.0082	0.0227	0.0020	0.0000	0.0071
post_hoc	NSTDB ma 0 dB	P-refit	B1	Δece	-0.0097	-0.0123	-0.0072	0.0000	0.0000	0.0000
post_hoc	NSTDB ma 0 dB	P-refit	B2	Δmacro_auc	0.0000	0.0000	0.0000	1.0000	1.0000	1.0000
post_hoc	NSTDB ma 0 dB	P-refit	B2	Δmacro_f1	0.0000	0.0000	0.0000	1.0000	1.0000	1.0000
post_hoc	NSTDB ma 0 dB	P-refit	B2	Δece	0.0000	0.0000	0.0000	1.0000	1.0000	1.0000
post_hoc	NSTDB ma 0 dB	P-rerun	B2	Δmacro_auc	-0.0057	-0.0077	-0.0039	0.0000	0.0000	0.0000
post_hoc	NSTDB ma 0 dB	P-rerun	B2	Δmacro_f1	-0.0096	-0.0167	-0.0026	0.0080	0.0077	1.0000
post_hoc	NSTDB ma 0 dB	P-rerun	B2	Δece	0.0172	0.0141	0.0200	0.0000	0.0000	0.0000
post_hoc	NSTDB em 12 dB	P-locked	B1	Δmacro_auc	0.0009	-0.0002	0.0019	0.1060	0.0934	1.0000
post_hoc	NSTDB em 12 dB	P-locked	B1	Δmacro_f1	0.0021	-0.0026	0.0070	0.3860	0.3893	1.0000
post_hoc	NSTDB em 12 dB	P-locked	B1	Δece	-0.0020	-0.0044	0.0004	0.1080	0.1038	1.0000
post_hoc	NSTDB em 12 dB	P-locked	B2	Δmacro_auc	-0.0003	-0.0007	0.0003	0.3340	0.3291	1.0000
post_hoc	NSTDB em 12 dB	P-locked	B2	Δmacro_f1	-0.0042	-0.0077	-0.0004	0.0340	0.0252	1.0000
post_hoc	NSTDB em 12 dB	P-locked	B2	Δece	0.0002	-0.0020	0.0025	0.8560	0.8562	1.0000
post_hoc	NSTDB em 12 dB	P-locked	B3	Δmacro_auc	0.0009	-0.0002	0.0020	0.1160	0.1028	1.0000
post_hoc	NSTDB em 12	P-locked	B3	Δmacro_f1	-0.0013	-0.0064	0.0037	0.5880	0.6067	1.0000

Family	Scenario	Policy	Comparator	Metric	Mean Δ	CI low	CI high	p (percentile)	p	p (Holm)
	dB									
post_hoc	NSTDB em 12 dB	P-locked	B3	Δece	-0.0039	-0.0069	-0.0009	0.0140	0.0096	1.0000
post_hoc	NSTDB em 12 dB	P-locked	A1	Δmacro_auc	-0.0001	-0.0004	0.0002	0.6840	0.6858	1.0000
post_hoc	NSTDB em 12 dB	P-locked	A1	Δmacro_f1	-0.0021	-0.0047	0.0006	0.1160	0.1276	1.0000
post_hoc	NSTDB em 12 dB	P-locked	A1	Δece	-0.0005	-0.0026	0.0018	0.6440	0.6427	1.0000
post_hoc	NSTDB em 12 dB	P-refit	B1	Δmacro_auc	0.0011	0.0001	0.0021	0.0400	0.0347	1.0000
post_hoc	NSTDB em 12 dB	P-refit	B1	Δmacro_f1	0.0042	-0.0008	0.0088	0.0940	0.0890	1.0000
post_hoc	NSTDB em 12 dB	P-refit	B1	Δece	-0.0018	-0.0041	0.0006	0.1120	0.1236	1.0000
post_hoc	NSTDB em 12 dB	P-refit	B2	Δmacro_auc	-0.0001	-0.0005	0.0004	0.7940	0.7619	1.0000
post_hoc	NSTDB em 12 dB	P-refit	B2	Δmacro_f1	-0.0021	-0.0050	0.0012	0.1840	0.1868	1.0000
post_hoc	NSTDB em 12 dB	P-refit	B2	Δece	0.0004	-0.0009	0.0016	0.5520	0.5419	1.0000
post_hoc	NSTDB em 12 dB	P-rerun	B2	Δmacro_auc	-0.0012	-0.0022	-0.0001	0.0280	0.0222	1.0000
post_hoc	NSTDB em 12 dB	P-rerun	B2	Δmacro_f1	-0.0034	-0.0090	0.0021	0.2460	0.2315	1.0000
post_hoc	NSTDB em 12 dB	P-rerun	B2	Δece	0.0046	0.0021	0.0073	0.0020	0.0005	0.1222
post_hoc	NSTDB em 6 dB	P-locked	B1	Δmacro_auc	0.0009	-0.0003	0.0021	0.1660	0.1573	1.0000
post_hoc	NSTDB em 6 dB	P-locked	B1	Δmacro_f1	0.0041	-0.0013	0.0094	0.1200	0.1386	1.0000
post_hoc	NSTDB em 6 dB	P-locked	B1	Δece	-0.0015	-0.0040	0.0011	0.2620	0.2552	1.0000
post_hoc	NSTDB em 6 dB	P-locked	B2	Δmacro_auc	-0.0001	-0.0006	0.0004	0.6440	0.6873	1.0000
post_hoc	NSTDB em 6 dB	P-locked	B2	Δmacro_f1	-0.0025	-0.0065	0.0015	0.2600	0.2278	1.0000
post_hoc	NSTDB em 6 dB	P-locked	B2	Δece	-0.0012	-0.0034	0.0009	0.2340	0.2597	1.0000
post_hoc	NSTDB em 6 dB	P-locked	B3	Δmacro_auc	0.0009	-0.0004	0.0021	0.1800	0.1747	1.0000
post_hoc	NSTDB em 6 dB	P-locked	B3	Δmacro_f1	-0.0000	-0.0055	0.0054	0.9680	0.9898	1.0000
post_hoc	NSTDB	P-	B3	Δece	-	-	-	0.0000	0.0017	0.4035

Family	Scenario	Policy	Comparator	Metric	Mean Δ	CI low	CI high	p (percentile)	p	p (Holm)
	em 6 dB	locked			0.0052	0.0083	0.0019			
post_hoc	NSTDB em 6 dB	P-locked	A1	Δmacro_auc	-0.0001	-0.0004	0.0001	0.2940	0.2788	1.0000
post_hoc	NSTDB em 6 dB	P-locked	A1	Δmacro_f1	-0.0025	-0.0058	0.0010	0.1720	0.1506	1.0000
post_hoc	NSTDB em 6 dB	P-locked	A1	Δece	-0.0020	-0.0042	0.0003	0.0940	0.0897	1.0000
post_hoc	NSTDB em 6 dB	P-refit	B1	Δmacro_auc	0.0012	0.0000	0.0024	0.0480	0.0557	1.0000
post_hoc	NSTDB em 6 dB	P-refit	B1	Δmacro_f1	0.0065	0.0011	0.0117	0.0180	0.0161	1.0000
post_hoc	NSTDB em 6 dB	P-refit	B1	Δece	0.0001	-0.0024	0.0025	0.9120	0.9246	1.0000
post_hoc	NSTDB em 6 dB	P-refit	B2	Δmacro_auc	0.0002	-0.0003	0.0007	0.4220	0.4357	1.0000
post_hoc	NSTDB em 6 dB	P-refit	B2	Δmacro_f1	-0.0000	-0.0027	0.0030	0.9380	0.9868	1.0000
post_hoc	NSTDB em 6 dB	P-refit	B2	Δece	0.0003	-0.0004	0.0011	0.3760	0.3852	1.0000
post_hoc	NSTDB em 6 dB	P-rerun	B2	Δmacro_auc	-0.0010	-0.0022	0.0001	0.0820	0.0851	1.0000
post_hoc	NSTDB em 6 dB	P-rerun	B2	Δmacro_f1	-0.0010	-0.0064	0.0047	0.7540	0.7254	1.0000
post_hoc	NSTDB em 6 dB	P-rerun	B2	Δece	0.0054	0.0030	0.0078	0.0000	0.0000	0.0024
post_hoc	NSTDB em 0 dB	P-locked	B1	Δmacro_auc	0.0012	-0.0009	0.0033	0.2540	0.2646	1.0000
post_hoc	NSTDB em 0 dB	P-locked	B1	Δmacro_f1	-0.0000	-0.0070	0.0075	0.9720	0.9952	1.0000
post_hoc	NSTDB em 0 dB	P-locked	B1	Δece	0.0020	-0.0001	0.0039	0.0600	0.0528	1.0000
post_hoc	NSTDB em 0 dB	P-locked	B2	Δmacro_auc	0.0000	-0.0000	0.0000	0.8920	0.9012	1.0000
post_hoc	NSTDB em 0 dB	P-locked	B2	Δmacro_f1	-0.0035	-0.0068	0.0001	0.0620	0.0480	1.0000
post_hoc	NSTDB em 0 dB	P-locked	B2	Δece	-0.0022	-0.0029	-0.0014	0.0000	0.0000	0.0000
post_hoc	NSTDB em 0 dB	P-locked	B3	Δmacro_auc	0.0012	-0.0009	0.0033	0.2540	0.2607	1.0000
post_hoc	NSTDB em 0 dB	P-locked	B3	Δmacro_f1	-0.0042	-0.0115	0.0030	0.2320	0.2531	1.0000
post_hoc	NSTDB em 0 dB	P-locked	B3	Δece	-0.0007	-0.0028	0.0014	0.5080	0.5218	1.0000
post_hoc	NSTDB em 0 dB	P-locked	A1	Δmacro_auc	0.0001	-0.0000	0.0003	0.1080	0.0954	1.0000
post_hoc	NSTDB em 0 dB	P-locked	A1	Δmacro_f1	-0.0031	-0.0065	0.0006	0.0960	0.0834	1.0000
post_hoc	NSTDB em 0 dB	P-locked	A1	Δece	-0.0025	-0.0033	-0.0018	0.0000	0.0000	0.0000
post_hoc	NSTDB em 0 dB	P-refit	B1	Δmacro_auc	0.0012	-0.0009	0.0033	0.2500	0.2676	1.0000
post_hoc	NSTDB em 0 dB	P-refit	B1	Δmacro_f1	0.0036	-0.0038	0.0108	0.3060	0.3258	1.0000
post_hoc	NSTDB em 0 dB	P-refit	B1	Δece	0.0042	0.0022	0.0060	0.0000	0.0000	0.0037
post_hoc	NSTDB	P-refit	B2	Δmacro_auc	-	-	0.0000	0.7120	0.6175	1.0000

Family	Scenario	Policy	Comparator	Metric	Mean Δ	CI low	CI high	p (percentile)	p	p (Holm)
	em 0 dB				0.0000	0.0001				
post_hoc	NSTDB em 0 dB	P-refit	B2	Δmacro_f1	0.0002	0.0000	0.0007	0.7700	0.3133	1.0000
post_hoc	NSTDB em 0 dB	P-refit	B2	Δece	- 0.0000	- 0.0001	0.0000	0.4760	0.4817	1.0000
post_hoc	NSTDB em 0 dB	P-rerun	B2	Δmacro_auc	- 0.0012	- 0.0033	0.0009	0.2540	0.2639	1.0000
post_hoc	NSTDB em 0 dB	P-rerun	B2	Δmacro_f1	0.0020	- 0.0051	0.0091	0.5720	0.5900	1.0000
post_hoc	NSTDB em 0 dB	P-rerun	B2	Δece	- 0.0009	- 0.0027	0.0010	0.3420	0.3529	1.0000

Table S5. Quality sensors: routing and performance of the pre-registered routing (P0/P0/P2, re-fitted temperatures)

Re-calibrated sensor: validation ROC-AUC (native fold 9) 0.761; lightweight sensor: 0.707. Disturbance-spanning tier edges: tertiles of the re-calibrated score on the fold-9 mixture (native + BW 0.75 + EMG 15 dB).

Scenario	Pipeline	Low-tier share	Macro-AUROC	Macro-F1	ECE	Worst-tier F1
Native	Identity, calibrated	—	0.9219	0.7398	0.0846	0.7217
Native	Fixed P2	—	0.9231	0.7419	0.0837	0.7298
Native	Adaptive, pre-registered sensor	0.3346	0.9223	0.7417	0.0847	0.7298
Native	Adaptive, re-calibrated sensor (native edges)	0.3276	0.9224	0.7415	0.0845	0.7274
Native	Adaptive, re-calibrated sensor (disturbance-spanning edges)	0.0542	0.9219	0.7392	0.0844	0.6917
Native	Adaptive, lightweight sensor	0.3438	0.9221	0.7408	0.0846	0.7296
BW 0.5	Identity, calibrated	—	0.9216	0.7367	0.0851	0.7180
BW 0.5	Fixed P2	—	0.9231	0.7416	0.0841	0.7228
BW 0.5	Adaptive, pre-registered sensor	0.7447	0.9231	0.7400	0.0851	0.7228
BW 0.5	Adaptive, re-calibrated sensor (native edges)	0.9870	0.9230	0.7415	0.0841	0.6444
BW 0.5	Adaptive, re-calibrated sensor (disturbance-spanning edges)	0.6019	0.9229	0.7397	0.0841	0.7058
BW 0.5	Adaptive, lightweight sensor	0.9852	0.9230	0.7413	0.0841	0.4000
BW 1.0	Identity, calibrated	—	0.9202	0.7346	0.0877	0.7122
BW 1.0	Fixed P2	—	0.9231	0.7415	0.0837	0.7197
BW 1.0	Adaptive, pre-registered sensor	0.7349	0.9228	0.7395	0.0850	0.7197
BW 1.0	Adaptive, re-calibrated sensor (native edges)	1.0000	0.9231	0.7415	0.0837	0.7415
BW 1.0	Adaptive, re-calibrated sensor (disturbance-spanning edges)	0.9629	0.9230	0.7410	0.0839	0.7164
BW 1.0	Adaptive, lightweight sensor	0.9754	0.9229	0.7409	0.0838	0.2000
BW 1.5	Identity, calibrated	—	0.9178	0.7274	0.0915	0.7060
BW 1.5	Fixed P2	—	0.9232	0.7414	0.0834	0.7212
BW 1.5	Adaptive, pre-registered sensor	0.8082	0.9229	0.7390	0.0852	0.7212
BW 1.5	Adaptive, re-calibrated sensor (native edges)	1.0000	0.9232	0.7414	0.0834	0.7414
BW 1.5	Adaptive, re-calibrated sensor (disturbance-spanning edges)	0.9991	0.9232	0.7414	0.0834	0.2000
BW 1.5	Adaptive, lightweight sensor	0.9648	0.9226	0.7395	0.0837	0.0000
EMG 20 dB	Identity, calibrated	—	0.9217	0.7376	0.0849	0.7376

Scenario	Pipeline	Low-tier share	Macro-AUROC	Macro-F1	ECE	Worst-tier F1
EMG 20 dB	Fixed P2	—	0.9230	0.7416	0.0841	0.7416
EMG 20 dB	Adaptive, pre-registered sensor	1.0000	0.9230	0.7416	0.0841	0.7416
EMG 20 dB	Adaptive, re-calibrated sensor (native edges)	0.8035	0.9227	0.7405	0.0844	0.7141
EMG 20 dB	Adaptive, re-calibrated sensor (disturbance-spanning edges)	0.0426	0.9216	0.7391	0.0852	0.6845
EMG 20 dB	Adaptive, lightweight sensor	0.9981	0.9229	0.7415	0.0841	0.3333
EMG 10 dB	Identity, calibrated	—	0.9207	0.7334	0.0923	0.7334
EMG 10 dB	Fixed P2	—	0.9223	0.7388	0.0892	0.7388
EMG 10 dB	Adaptive, pre-registered sensor	1.0000	0.9223	0.7388	0.0892	0.7388
EMG 10 dB	Adaptive, re-calibrated sensor (native edges)	1.0000	0.9223	0.7388	0.0892	0.7388
EMG 10 dB	Adaptive, re-calibrated sensor (disturbance-spanning edges)	1.0000	0.9223	0.7388	0.0892	0.7388
EMG 10 dB	Adaptive, lightweight sensor	1.0000	0.9223	0.7388	0.0892	0.7388
EMG 5 dB	Identity, calibrated	—	0.9178	0.7232	0.1114	0.7232
EMG 5 dB	Fixed P2	—	0.9201	0.7306	0.1064	0.7306
EMG 5 dB	Adaptive, pre-registered sensor	1.0000	0.9201	0.7306	0.1064	0.7306
EMG 5 dB	Adaptive, re-calibrated sensor (native edges)	1.0000	0.9201	0.7306	0.1064	0.7306
EMG 5 dB	Adaptive, re-calibrated sensor (disturbance-spanning edges)	1.0000	0.9201	0.7306	0.1064	0.7306
EMG 5 dB	Adaptive, lightweight sensor	1.0000	0.9201	0.7306	0.1064	0.7306
NSTDB bw 12 dB	Identity, calibrated	—	0.9211	0.7351	0.0862	0.7072
NSTDB bw 12 dB	Fixed P2	—	0.9231	0.7418	0.0839	0.7154
NSTDB bw 12 dB	Adaptive, pre-registered sensor	0.6993	0.9228	0.7407	0.0850	0.7154
NSTDB bw 12 dB	Adaptive, re-calibrated sensor (native edges)	1.0000	0.9231	0.7418	0.0839	0.7418
NSTDB bw 12 dB	Adaptive, re-calibrated sensor (disturbance-spanning edges)	0.9018	0.9231	0.7421	0.0840	0.7358
NSTDB bw 12 dB	Adaptive, lightweight sensor	0.9768	0.9228	0.7412	0.0840	0.6450
NSTDB bw 6 dB	Identity, calibrated	—	0.9197	0.7321	0.0896	0.7240
NSTDB bw 6 dB	Fixed P2	—	0.9231	0.7408	0.0841	0.7331
NSTDB bw 6 dB	Adaptive, pre-registered sensor	0.8888	0.9229	0.7402	0.0848	0.7331
NSTDB bw 6 dB	Adaptive, re-calibrated sensor (native edges)	1.0000	0.9231	0.7408	0.0841	0.7408
NSTDB bw 6 dB	Adaptive, re-calibrated sensor (disturbance-spanning edges)	1.0000	0.9231	0.7408	0.0841	0.7408
NSTDB bw 6 dB	Adaptive, lightweight sensor	0.9815	0.9228	0.7400	0.0842	0.6566
NSTDB bw 0 dB	Identity, calibrated	—	0.9145	0.7180	0.1035	0.7180
NSTDB bw 0 dB	Fixed P2	—	0.9227	0.7403	0.0861	0.7403
NSTDB bw 0 dB	Adaptive, pre-registered sensor	1.0000	0.9227	0.7403	0.0861	0.7403
NSTDB bw 0 dB	Adaptive, re-calibrated sensor (native edges)	1.0000	0.9227	0.7403	0.0861	0.7403

Scenario	Pipeline	Low-tier share	Macro-AUROC	Macro-F1	ECE	Worst-tier F1
NSTDB bw 0 dB	Adaptive, re-calibrated sensor (disturbance-spanning edges)	1.0000	0.9227	0.7403	0.0861	0.7403
NSTDB bw 0 dB	Adaptive, lightweight sensor	0.9801	0.9223	0.7392	0.0862	0.6588
NSTDB ma 12 dB	Identity, calibrated	—	0.9214	0.7397	0.0879	0.7391
NSTDB ma 12 dB	Fixed P2	—	0.9228	0.7425	0.0847	0.7416
NSTDB ma 12 dB	Adaptive, pre-registered sensor	0.9866	0.9227	0.7422	0.0848	0.7416
NSTDB ma 12 dB	Adaptive, re-calibrated sensor (native edges)	0.9991	0.9228	0.7425	0.0847	0.4000
NSTDB ma 12 dB	Adaptive, re-calibrated sensor (disturbance-spanning edges)	0.3772	0.9221	0.7397	0.0865	0.4814
NSTDB ma 12 dB	Adaptive, lightweight sensor	0.9680	0.9227	0.7428	0.0850	0.7398
NSTDB ma 6 dB	Identity, calibrated	—	0.9195	0.7307	0.0956	0.7307
NSTDB ma 6 dB	Fixed P2	—	0.9220	0.7432	0.0920	0.7432
NSTDB ma 6 dB	Adaptive, pre-registered sensor	1.0000	0.9220	0.7432	0.0920	0.7432
NSTDB ma 6 dB	Adaptive, re-calibrated sensor (native edges)	1.0000	0.9220	0.7432	0.0920	0.7432
NSTDB ma 6 dB	Adaptive, re-calibrated sensor (disturbance-spanning edges)	0.9778	0.9220	0.7434	0.0922	0.7415
NSTDB ma 6 dB	Adaptive, lightweight sensor	0.9449	0.9216	0.7427	0.0924	0.6933
NSTDB ma 0 dB	Identity, calibrated	—	0.9123	0.7059	0.1339	0.7059
NSTDB ma 0 dB	Fixed P2	—	0.9180	0.7215	0.1242	0.7215
NSTDB ma 0 dB	Adaptive, pre-registered sensor	1.0000	0.9180	0.7215	0.1242	0.7215
NSTDB ma 0 dB	Adaptive, re-calibrated sensor (native edges)	1.0000	0.9180	0.7215	0.1242	0.7215
NSTDB ma 0 dB	Adaptive, re-calibrated sensor (disturbance-spanning edges)	1.0000	0.9180	0.7215	0.1242	0.7215
NSTDB ma 0 dB	Adaptive, lightweight sensor	0.9115	0.9170	0.7201	0.1245	0.7181
NSTDB em 12 dB	Identity, calibrated	—	0.9211	0.7348	0.0925	0.7142
NSTDB em 12 dB	Fixed P2	—	0.9223	0.7411	0.0901	0.7204
NSTDB em 12 dB	Adaptive, pre-registered sensor	0.7739	0.9222	0.7390	0.0907	0.7204
NSTDB em 12 dB	Adaptive, re-calibrated sensor (native edges)	1.0000	0.9223	0.7411	0.0901	0.7411
NSTDB em 12 dB	Adaptive, re-calibrated sensor (disturbance-spanning edges)	0.7753	0.9221	0.7393	0.0902	0.7297
NSTDB em 12 dB	Adaptive, lightweight sensor	0.9754	0.9221	0.7417	0.0903	0.2000
NSTDB em 6 dB	Identity, calibrated	—	0.9170	0.7192	0.1172	0.7095
NSTDB em 6 dB	Fixed P2	—	0.9180	0.7258	0.1171	0.7161

Scenario	Pipeline	Low-tier share	Macro-AUROC	Macro-F1	ECE	Worst-tier F1
NSTDB em 6 dB	Adaptive, pre-registered sensor	0.8758	0.9182	0.7258	0.1173	0.7161
NSTDB em 6 dB	Adaptive, re-calibrated sensor (native edges)	1.0000	0.9180	0.7258	0.1171	0.7258
NSTDB em 6 dB	Adaptive, re-calibrated sensor (disturbance-spanning edges)	1.0000	0.9180	0.7258	0.1171	0.7258
NSTDB em 6 dB	Adaptive, lightweight sensor	0.9620	0.9179	0.7268	0.1170	0.7148
NSTDB em 0 dB	Identity, calibrated	—	0.8999	0.6511	0.2164	0.5200
NSTDB em 0 dB	Fixed P2	—	0.9011	0.6546	0.2206	0.4600
NSTDB em 0 dB	Adaptive, pre-registered sensor	0.9977	0.9011	0.6548	0.2206	0.5200
NSTDB em 0 dB	Adaptive, re-calibrated sensor (native edges)	1.0000	0.9011	0.6546	0.2206	0.6546
NSTDB em 0 dB	Adaptive, re-calibrated sensor (disturbance-spanning edges)	1.0000	0.9011	0.6546	0.2206	0.6546
NSTDB em 0 dB	Adaptive, lightweight sensor	0.9347	0.9011	0.6553	0.2204	0.6432

Table S6. Morphology-sensitive analyses

(a) Sub-class AUROC: parent super-class score, records with the sub-class vs. records without the parent super-class (sub-classes with ≥ 10 positive test records).

Scenario	superclass	subclass	n_pos	identity_calibrated	fixed_P2	adaptive_P0P0P2_refit
Native	MI	AMI	306	0.9303	0.9303	0.9304
Native	MI	IMI	327	0.9195	0.9209	0.9182
Native	MI	LMI	20	0.7885	0.7882	0.7863
Native	STTC	ISCA	93	0.9483	0.9515	0.9494
Native	STTC	ISCI	40	0.9306	0.9326	0.9311
Native	STTC	ISC_	128	0.9742	0.9768	0.9748
Native	STTC	NST_	77	0.9229	0.9245	0.9231
Native	STTC	STTC	222	0.9146	0.9165	0.9154
Native	CD	CLBBB	54	0.9987	0.9988	0.9986
Native	CD	CRBBB	54	0.9986	0.9987	0.9988
Native	CD	IRBBB	112	0.9300	0.9301	0.9316
Native	CD	IVCD	79	0.7538	0.7522	0.7526
Native	CD	LAFB/LPFB	179	0.9600	0.9599	0.9598
Native	CD	_AVB	82	0.9436	0.9489	0.9460
Native	HYP	LAO/LAE	42	0.7630	0.7648	0.7635
Native	HYP	LVH	214	0.9448	0.9451	0.9450
Native	HYP	RAO/RAE	10	0.7885	0.8091	0.7985
Native	HYP	RVH	12	0.8019	0.8143	0.8036
BW 1.5	MI	AMI	306	0.9286	0.9304	0.9300
BW 1.5	MI	IMI	327	0.9133	0.9210	0.9199
BW 1.5	MI	LMI	20	0.7979	0.7890	0.7899
BW 1.5	STTC	ISCA	93	0.9432	0.9510	0.9497
BW 1.5	STTC	ISCI	40	0.9265	0.9317	0.9334
BW 1.5	STTC	ISC_	128	0.9714	0.9769	0.9773
BW 1.5	STTC	NST_	77	0.9201	0.9242	0.9254
BW 1.5	STTC	STTC	222	0.9151	0.9167	0.9162
BW 1.5	CD	CLBBB	54	0.9985	0.9988	0.9987
BW 1.5	CD	CRBBB	54	0.9978	0.9987	0.9986

Scenario	superclass	subclass	n_pos	identity_calibrated	fixed_P2	adaptive_P0P0P2_refit
BW 1.5	CD	IRBBB	112	0.9243	0.9303	0.9292
BW 1.5	CD	IVCD	79	0.7498	0.7519	0.7515
BW 1.5	CD	LAFB/LPFB	179	0.9587	0.9600	0.9603
BW 1.5	CD	_AVB	82	0.9381	0.9488	0.9474
BW 1.5	HYP	LAO/LAE	42	0.7496	0.7644	0.7665
BW 1.5	HYP	LVH	214	0.9387	0.9457	0.9457
BW 1.5	HYP	RAO/RAE	10	0.7802	0.8088	0.8041
BW 1.5	HYP	RVH	12	0.7765	0.8126	0.8086

(b) Sensitivity at 90% specificity per super-class.

Scenario	class	identity_calibrated	fixed_P2	adaptive_P0P0P2_refit
Native	NORM	0.794	0.794	0.800
Native	MI	0.725	0.749	0.736
Native	STTC	0.793	0.795	0.802
Native	CD	0.786	0.786	0.786
Native	HYP	0.695	0.706	0.710
BW 1.5	NORM	0.805	0.792	0.794
BW 1.5	MI	0.713	0.749	0.751
BW 1.5	STTC	0.797	0.800	0.791
BW 1.5	CD	0.782	0.788	0.790
BW 1.5	HYP	0.676	0.710	0.698

(c) Waveform fidelity per operator (native test records; lead-records).

operator	records	n_records	ST_abs_change_median_uV	ST_abs_change_p95_uV	ST_change_ge_50uV_%	Q_abs_change_median_uV	Q_change_ge_50uV_%	R_rel_change_median_%
P2	all	2158	5.16	30.46	1.93	7.88	3.24	3.36
P2	MI records	550	5.00	33.47	2.35	8.47	4.70	4.18
P2	STTC records	521	5.31	35.56	2.40	8.11	4.25	3.48
P3	all	2158	5.23	30.42	1.99	9.66	5.00	5.47
P3	MI records	550	5.05	33.25	2.35	10.45	7.47	6.29
P3	STTC records	521	5.40	35.64	2.54	9.71	6.37	5.41
P4	all	2158	14.70	88.42	14.30	12.28	6.89	4.71
P4	MI records	550	11.00	70.54	9.42	11.48	7.05	4.95
P4	STTC records	521	10.45	66.40	8.05	10.35	5.58	4.01
P6	all	2158	6.01	31.35	1.99	9.30	4.80	6.23
P6	MI records	550	6.07	34.33	2.35	10.55	6.68	7.71
P6	STTC records	521	6.23	36.11	2.62	9.77	5.68	6.45

operator	records	n_records	ST_abs_change_median_uV	ST_abs_change_p95_uV	ST_change_ge_50uV_%	Q_abs_change_median_uV	Q_change_ge_50uV_%	R_rel_change_median_%
P7	all	2158	6.31	31.79	2.06	34.31	34.62	27.60
P7	MI records	550	5.93	34.51	2.47	32.22	32.12	27.57
P7	STTC records	521	6.51	35.84	2.62	35.14	36.95	26.55
P8	all	2158	12.33	69.98	9.49	7.62	3.39	3.12
P8	MI records	550	11.21	66.04	8.76	8.56	4.77	4.10
P8	STTC records	521	11.80	73.96	10.01	8.25	4.09	3.32

Table S7. Fine-tuning logs (post hoc analysis 5)

Up to five epochs, AdamW, learning rate 10^{-4}; the best epoch (or the unchanged model if no epoch improved by > 10^{-4}) was kept.

Condition	Seed	Val. AUROC before	Best val. AUROC	Per-epoch val. AUROC
ft_policy	20260901	0.9188	0.9203	0.9203 / 0.9200 / 0.9199 / 0.9202 / 0.9192
ft_policy	20260902	0.9206	0.9230	0.9230 / 0.9226 / 0.9202 / 0.9199 / 0.9213
ft_policy	20260903	0.9187	0.9187	0.9182 / 0.9172 / 0.9177 / 0.9164 / 0.9157
ft_p2	20260901	0.9191	0.9204	0.9204 / 0.9204 / 0.9197 / 0.9200 / 0.9194
ft_p2	20260902	0.9209	0.9226	0.9226 / 0.9223 / 0.9202 / 0.9200 / 0.9214
ft_p2	20260903	0.9188	0.9188	0.9186 / 0.9173 / 0.9182 / 0.9169 / 0.9163
ft_identity	20260901	0.9178	0.9189	0.9189 / 0.9186 / 0.9183 / 0.9180 / 0.9174
ft_identity	20260902	0.9190	0.9217	0.9217 / 0.9213 / 0.9187 / 0.9181 / 0.9198
ft_identity	20260903	0.9175	0.9175	0.9173 / 0.9162 / 0.9166 / 0.9148 / 0.9147

Figure S1. Pareto fronts of the expanded-bank searches (post hoc analysis 2), projected on macro-AUROC and worst-tier macro-F1.

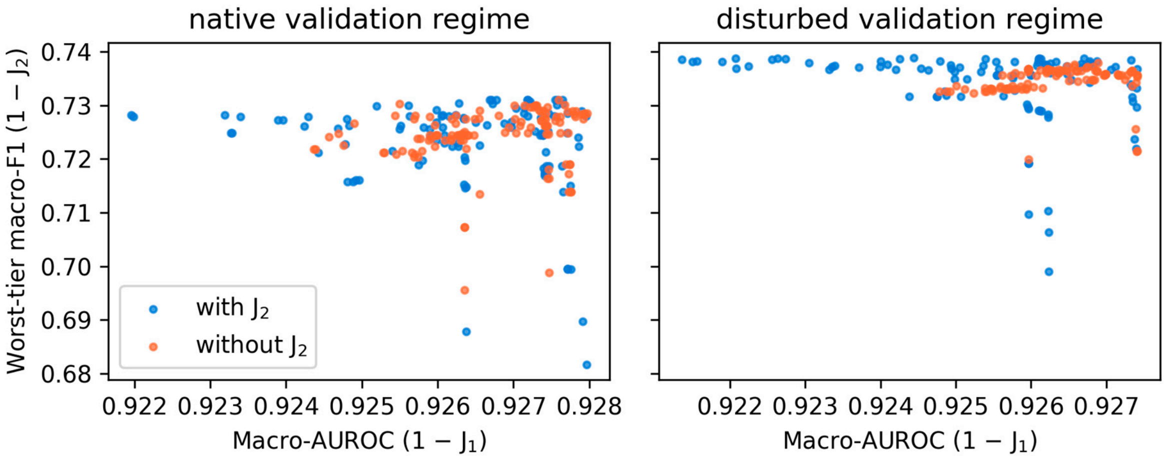

Supplement: Supplementary file 1 [file biomimetics-11-00744-s001.zip › biomimetics-4592874-supplementary.pdf]
